# Individualized portal pressure gradient threshold based on liver function categories in preventing rebleeding after TIPS

**DOI:** 10.1007/s12072-023-10489-x

**Published:** 2023-02-17

**Authors:** Yifu Xia, Jun Tie, Guangchuan Wang, Yuzheng Zhuge, Hao Wu, Hui Xue, Jiao Xu, Feng Zhang, Lianhui Zhao, Guangjun Huang, Mingyan Zhang, Bo Wei, Peijie Li, Wei Wu, Chao Chen, Chengwei Tang, Chunqing Zhang

**Affiliations:** 1 Department of Gastroenterology, Shandong Provincial Hospital, Shandong University, Jinan, Shandong China; 2 National Clinical Research Center for Digestive Diseases and Xijing Hospital of Digestive Diseases, Air Force Medical University, Xi’an, Shaanxi China; 3 Department of Gastroenterology, Shandong Provincial Hospital Affiliated to Shandong First Medical University, Jinan, Shandong China; 4 Department of Biostatistics, School of Public Health, Shandong University, Jinan, Shandong China; 5 Department of Gastroenterology, Affiliated Drum Tower Hospital of Nanjing University Medical School, Nanjing, Jiangsu China; 6 Department of Gastroenterology and Hepatology, West China Hospital, Chengdu, Sichuan China; 7 Gastroenterology of the First Affiliated Hospital of Xi’an Jiao Tong University, Xi’an, Shaanxi China; 8 Department of Gastroenterology, The First Affiliated Hospital of Wenzhou Medical University, Wenzhou, Zhejiang China

**Keywords:** Portal pressure gradient, Variceal rebleeding, Transjugular intrahepatic portosystemic shunt, Individualized therapy, Portal hypertension, Decompensated liver cirrhosis, Hepatic encephalopathy, Mortality, Covered stent, Child–Pugh class

## Abstract

**Background:**

The evidence in Portal pressure gradient (PPG) < 12 mmHg after transjugular intrahepatic portosystemic shunt (TIPS) for preventing rebleeding mostly comes from observations in uncovered stents era. Moreover, association between Child–Pugh classes and post-TIPS hepatic encephalopathy (HE) has indicated that tolerance of PPG reduction depends on liver function. This study aimed to investigate the optimal PPG for covered TIPS and explore the optimal threshold tailored to the Child–Pugh classes to find individualized PPG to balance rebleeding and overt HE.

**Methods:**

This multicenter retrospective study analyzed rebleeding, OHE, and mortality of patients associated with post-TIPS PPGs (8, 10, 12, and 14 mmHg) in the entire cohort and among different Child–Pugh classes. Propensity score matching (PSM) and competing risk analyses were performed for sensitivity analyses.

**Results:**

We included 2100 consecutively screened patients undergoing TIPS. In all patients, PPG < 12 mmHg reduced rebleeding after TIPS (*p* = 0.022). In Child–Pugh class A, none of the PPG thresholds were discriminative of clinical outcomes. In Child–Pugh class B, 12 mmHg (*p* = 0.022) and 14 mmHg (*p* = 0.037) discriminated rebleeding, but 12 mmHg showed a higher net benefit. In Child–Pugh class C, PPG < 14 mmHg had a lower rebleeding incidence (*p* = 0.017), and exhibited more net benefit than 12 mmHg.

**Conclusion:**

Different PPG standards may be required for patients with different liver function categories. A PPG threshold < 12 mmHg might be suitable for patients in Child–Pugh class B, while < 14 mmHg might be optimal for patients in Child–Pugh class C.

**Graphical Abstract:**

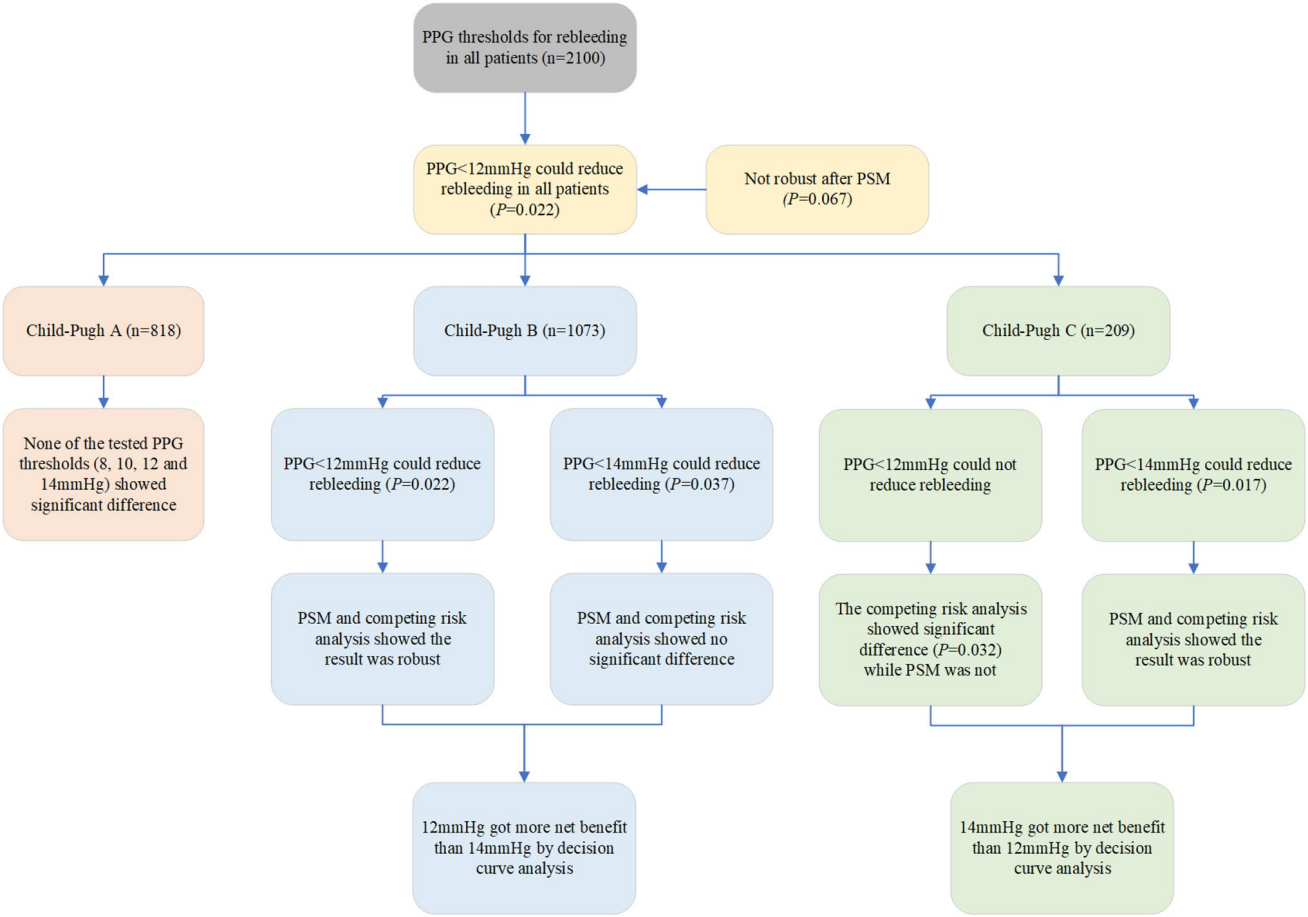

**Supplementary Information:**

The online version contains supplementary material available at 10.1007/s12072-023-10489-x.

## Introduction

Transjugular intrahepatic portosystemic shunt (TIPS) is commonly used to treat portal hypertension and its complications. The role of TIPS in the management of portal hypertension complications has been studied. It is recommended as the first-line treatment for patients with acute variceal bleeding and as the treatment choice for patients who fail standard β-blocker/endoscopy to prevent variceal rebleeding [1, 2]. The reduction in the portal pressure gradient (PPG) because of TIPS causes the following two major concerns: how to achieve efficient prevention of rebleeding and how to avoid excessive hepatic encephalopathy (HE) caused by over-shunt.

According to past practices and the Baveno VII recommendation [1, 3], a target PPG of < 12 mmHg or > 50% of the baseline is expected to prevent rebleeding [4–9]. However, most studies on hemodynamic targets were performed before the introduction of covered stents and have not been adequately updated [4, 5]. HE risk is still high with covered stents for TIPS, even when using the current PPG standard [10–12], limiting their widespread clinical application. Therefore, it is important to explore the PPG threshold suitable for covered stents for TIPS to prevent rebleeding and reduce overt HE (OHE) risk [13].

There is accumulating evidence that Child–Pugh classes /scores are one of the best predictors of post-TIPS HE [14–16]. High Child–Pugh classes /scores have shown the greatest potential for predicting post-TIPS HE, indicating different tolerances to PPG reduction in patients with different liver function classes. Therefore, we hypothesized that the optimal PPG threshold should be adjusted according to the Child–Pugh classes.

In this multicenter retrospective study, we investigated the effect of PPG reduction in patient clinical outcomes after TIPS to examine the beneficial effect of PPG after covered TIPS and to explore whether an individualized PPG threshold is needed in patients with different Child–Pugh classes.

## Methods

### Patients

This retrospective study enrolled 3633 patients who received treatment using TIPS for variceal bleeding at six academic hospitals in China between January 2010 and June 2020. The disposition of patients is shown as a flow chart in Supplementary Fig. 1. All hospitals had a gastroenterology/liver unit, and experienced clinicians that perform TIPS regularly. All patients received etiological treatment with continued antiviral therapy, alcohol abstinence, or ursodeoxycholic acid depending on the cause of cirrhosis. And all patients were treated with medication or endoscopic therapy before TIPS according to the guidelines of portal hypertension [1]. Lactulose or rifaximin was used to prevent hepatic encephalopathy for 3 months after TIPS.

The study inclusion criteria were as follows: (1) cirrhosis diagnosis (based on clinical signs, laboratory and imaging tests, or liver biopsy) and (2) TIPS performed for secondary prophylaxis of variceal bleeding. The exclusion criteria were as follows: (1) age < 14 or > 74 years; (2) Child–Pugh score > 13; (3) TIPS with uncovered stents, hepatocellular carcinoma, or another advanced tumor; (4) previous TIPS implantation; (5) portocaval surgery, surgical shunting, or splenectomy; and (6) unrelieved bile duct obstruction; (7) heart failure ≥ NYHA III, coronary heart disease, or arrhythmia requiring treatment; (8) partially or completely occlusive portal vein; (9) cavernous transformation; (10) loss of essential data such as PPG, biochemical and hematological tests, or imaging tests.

### Study design

The primary endpoint of this study was all-cause rebleeding. The secondary endpoints were all-cause mortality and development of OHE. Physical examination, biochemical and hematological tests, and abdominal ultrasound data were obtained. Follow-up time was defined as the interval from the date of admission to death, liver transplantation, last visit, or study end (September 6, 2021). OHE was defined as HE grade ≥ 2 according to the West Haven-modified criteria [17, 18].

### TIPS procedure and PPG measurement

Participating centers are fully experienced in TIPS procedure and conformed to the TIPS Clinical Practice Guidelines by Chinese Medical Association [19]. An expanded polytetrafluoroethylene-covered stent with a suitable diameter (6, 8, or 10 mm), Viatorr TIPS endoprosthesis (Gore, Inc.; Flagstaff, AZ, USA), or Fluency stent (Bard, Inc., Tempe, AZ, USA) was deployed using the transjugular approach and was initially dilated to 6, 8, or 10 mm. The selection of stent diameter is mainly based on the operator's experience and patient’s characteristics of pre-TIPS PPG, pre-TIPS HE, liver function and the availability of stents. PPG was obtained preoperatively (baseline PPG) and immediately after stent placement (immediate PPG). PPG was measured as the difference between the pressure measured in the portal vein (PV) and inferior vena cava (rather than the right atrium) with a 5.5F Fogarty Thru-Lumen Embolectomy Catheter (Edwards Lifesciences LLC, Irvine, USA). All TIPS procedures were performed under local anesthesia (In China, it is rare for TIPS to be performed under general anesthesia, except for emergency TIPS; it avoids the influence of deep sedation on PPG). Permanent tracings from all pressure measurements were obtained using a recalibrated Mac-Lab electromechanical transducer and a polygraph (GE Healthcare, Freiburg, Germany).

In addition, all patients enrolled in this study underwent TIPS for secondary prophylaxis of variceal bleeding, which avoided the influence of emergency vasoconstrictive drugs on PPG measurement. Therefore, the immediate PPG in this study could represent the actual PV pressure in these patients. PPG was measured according to uniform procedures at each medical center, and all PPG measurements that did not follow the established procedures were excluded.

### Statistical analysis

Quantitative variables were reported as medians (quartiles) and compared using the Mann–Whitney U test because they were non-normally distributed. Qualitative variables were reported as frequencies (percentages) and compared using the χ^2^ test. Multivariate Cox proportional hazards analysis was used to identify independent risk factors, and hazard ratios (HR) with 95% confidence intervals (95% CI) were reported. The cumulative incidences of OHE and rebleeding were estimated in a competing risk setting, where death or liver transplantation competed with the event of interest. When mortality was estimated, liver transplantation and non-cirrhotic death were considered as competing risks. Decision curve analysis (DCA) was performed to determine which of the two potential PPGs was better.

Propensity score matching (PSM) was performed between the different post-PPG groups to provide a balanced baseline. We took a 1: n PSM match, where n was as large as possible to reduce data loss. The nearest neighbor matching method with a 0.4 caliper was used to construct the control group. The standard mean difference (SMD) was used to assess whether the groups were comparable after PSM. SMD < 0.2 indicated a small difference between groups [20]. The PSM details for each group are presented in Supplementary Tables 2–17.

All results with a two-sided *p* < 0.05 were considered statistically significant. All analyses were performed using SPSS v.25 (IBM; Armonk, NY, USA), GraphPad Prism v.8.0.2 (San Diego, CA, USA), and R v.4.1.0 (http://www.R-project.org/) with the Matchit, survival, and cmprsk packages.

## Results

### Patient characteristics

We screened 3663 consecutive patients with cirrhosis who underwent TIPS for secondary prophylaxis of variceal bleeding and were admitted to the six participating hospitals between January 2010 and June 2020; finally, 2100 patients were included in the study. All patients with non-standard PPG measurements, incomplete basic information, advanced liver cancer, and cardiopulmonary diseases affecting survival were excluded. The median follow-up period was 32.38 (19.03–56.37) months. The baseline characteristics of the patients are summarized in Table 1. Before TIPS, 714 (34.0%) patients were treated by endoscopy, including 510 (24.3%) patients with EVL and 693 (33.0%) patients with ECI. The frequency of EVL and ECI was 1.7 and 1.9 for one patient on average. There were 1413 (67.29%) patients with ascites before TIPS, and 163 (7.8%) of them were refractory ascites which was treated by large volume paracentesis and diuretic. Of all patients, 47.6% had a portal vein thrombosis with stenosis < 50%, including the main trunk (38.8%) or branch (38.8%). A total of 818 (38.95%), 1073 (51.10%), and 209 (9.95%) patients were graded as Child–Pugh A, B, and C, respectively. Stent diameters of 6, 8, and 10 mm were used in 95 (4.52%), 1904 (90.67%), and 101 (4.81%) patients, respectively. The median PPG decreased from 24 to 8.8 mmHg in the entire cohort. Difference of the portal pressure according to stent diameters was shown in Supplementary Table 18. Demographic and baseline characteristics are shown in Table 1.

**Table 1:** Demographic and baseline characteristics

Characteristic	Count
Age (years)	52 (44–60)
Sex	
Male	1351 (64.33%)
Female	749 (35.67%)
Cirrhosis etiology	
Hepatic virus	1414 (67.33%)
Other	686 (32.67%)
Platelet (10^9^/L)	59 (42–88)
Hemoglobin (g/L)	81 (70–95)
Albumin (g/L)	33.7 (30.2–37.4)
Total bilirubin (μmol/L)	20.9 (14.4–30.1)
Creatinine (μmol/L)	73 (59–88)
Sodium (mmol/L)	139.7 (137.2–142)
PT length (s)	1.6 (0–3.3)
International normalized ratio	1.31 (1.19–1.47)
Child–Pugh class	
A	818 (38.95%)
B	1073 (51.10%)
C	209 (9.95%)
Child–Pugh score (points)	7 (6–8)
MELD score (points)	10 (9–13)
MELD-Na score (points)	11 (9–13)
Portal vein thrombosis	999 (47.6%)
Main trunk	815 (38.8%)
Branch	815 (38.8%)
Ascites before TIPS	
Present	1413 (67.29%)
Absent	687 (32.71%)
OHE before TIPS	
Present	22 (1.00%)
Absent	2078 (99.00%)
Red-color sign^a^	
Present	1333 (63.48%)
Absent	364 (17.33%)
Unknown	403 (19.19%)
Pre-TIPS PPG (mmHg)	24 (20–27.2)
Post-TIPS PPG (mmHg)	8.8 (6–11)
PPG decrease rates (%)	63.73 (52.94–73.01)
Stent diameter	
6 mm	95 (4.52%)
8 mm	1904 (90.67%)
10 mm	101 (4.81%)

*PT* prothrombin time, *MELD* model for end-stage liver disease, *PPG* portal pressure gradient, *TIPS* transjugular intrahepatic portosystemic shunt

^a^only 1697 patients underwent endoscopy before TIPS

### Clinical outcomes

**Fig. 1:**
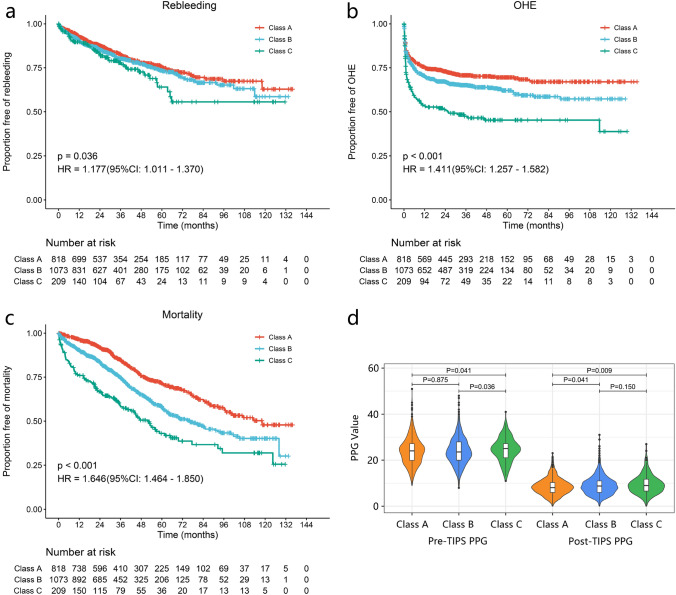
Outcomes and PPG of patients in different Child–Pugh classes. The proportion of patients without **a** rebleeding, **b** OHE, and **c** mortality in the different Child–Pugh classes. **d** Pre- and post-TIPS PPG of patients in different Child–Pugh classes

Rebleeding occurred in 409 patients during the follow-up period, resulting in a cumulative incidence of rebleeding of 19.48%. In all rebleeding patients, 184 (45.0%) patients underwent shunt dysfunction. Among shunt dysfunction patients, 102 (55.4%) received stent recanalization. All other 307 (75.1%) out of these 409 rebleeding patients were treated with medication and endoscopy. OHE occurred in 704 patients during the follow-up period, with a cumulative incidence of 30.52%. Twenty-two patients had pre-TIPS HE, and all of them had OHE 1 year after TIPS. Twenty (1.0%) patients had TIPS reduction for refractory HE, and there were 298 (14.2%) patients with stent stenosis after TIPS. Overall, 640 patients died, resulting in a cumulative mortality incidence of 28.76%. During follow-up, 110 (5.2%) patients died of liver failure. Clinical outcomes differed significantly among patients with different Child–Pugh classes (Fig. 1). The results of multivariate Cox regression analysis are shown in Supplementary Table 1.

### The effect of PPG reduction on clinical outcomes in all patients

#### *PPG* < *12 mmHg in all patients*

**Fig. 2:**
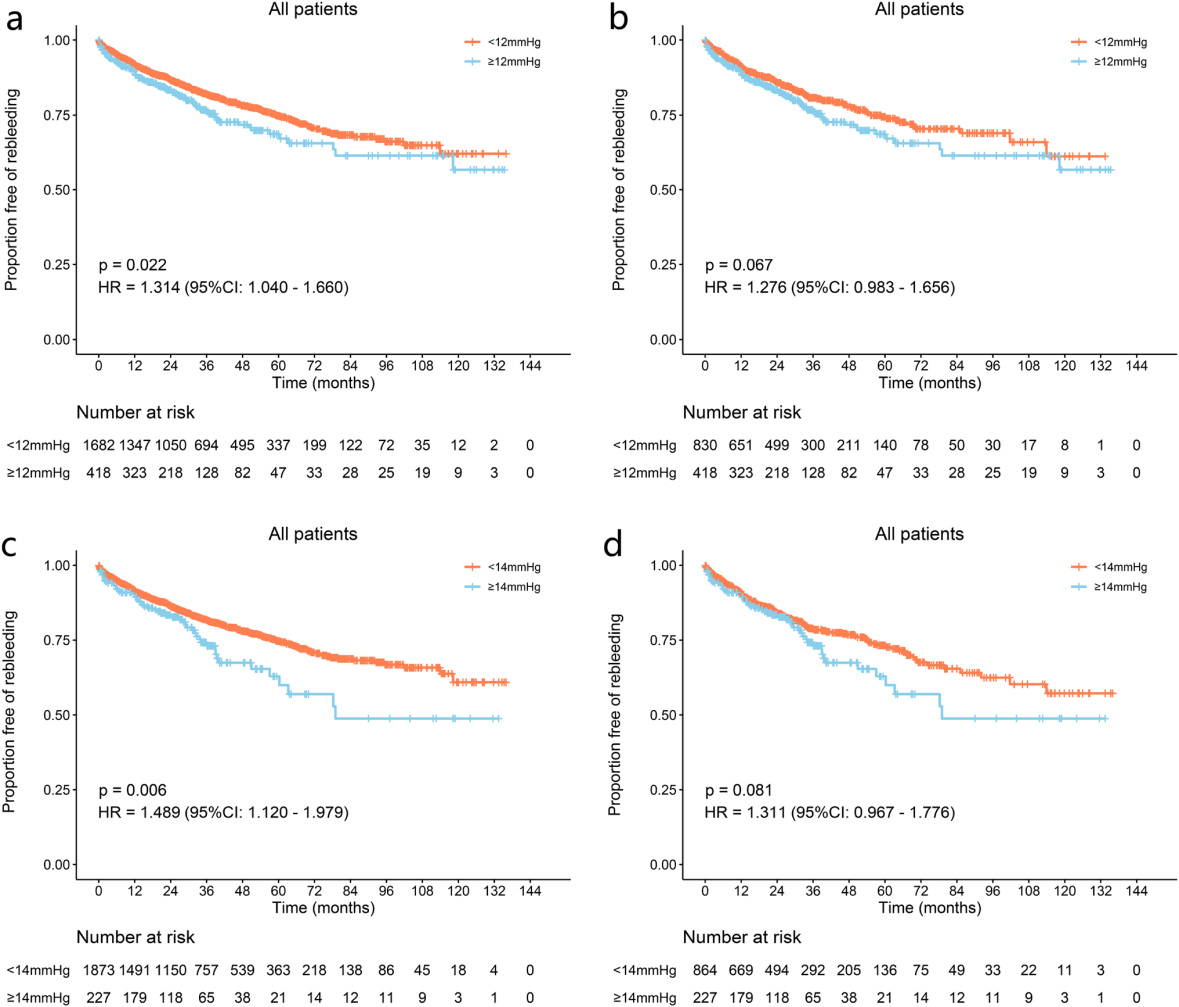
Proportion of patients without rebleeding with a 12 or 14 mmHg post-TIPS PPG threshold. The proportion of patients without rebleeding **a** before and **b** after PSM with a 12 mmHg PPG threshold. The proportion of patients without rebleeding **c** before and **d** after PSM with a 14 mmHg PPG threshold

To validate different PPG values as the target to prevent bleeding, we divided all patients into two groups according to post-TIPS PPG < 12 mmHg or ≥ 12 mmHg. In the < 12 mmHg group, 1682 patients had a post-TIPS cumulative rebleeding incidence of 37.95%. Meanwhile, in the ≥ 12 mmHg group, 418 patients had a post-TIPS cumulative rebleeding incidence 43.29%. Significant differences between the two groups were observed for rebleeding (*p* = 0.022, HR = 1.314, 95% CI 1.040–1.660), but not for OHE and mortality (*p* = 0.066, HR = 0.833, 95% CI 0.686–1.012 and *p* = 0.909, HR = 0.988, 95% CI 0.806–1.212, respectively) (Fig. 2 and Supplementary Fig. 2). After 1:2 PSM, the significance of between-group differences in rebleeding was not robust (38.81 vs. 43.29%, *p* = 0.067, HR = 1.267, 95% CI 0.983–1.656), and no significant difference was detected in terms of OHE and mortality (*p* = 0.192, HR = 0.868, 95% CI 0.701–1.074 and *p* = 0.897, HR = 0.985, 95% CI 0.788–1.233, respectively) (Fig. 2 and Supplementary Fig. 2). Competing risk analysis (Fine-Gray test) showed similar results (Supplementary Fig. 3).

#### Other PPG thresholds of 8, 10, and 14 mmHg in all patients

We also explored other PPG values (8, 10, and 14 mmHg) as potential thresholds. A post-TIPS PPG < 8 or 10 mmHg could not achieve less rebleeding without increasing the OHE (Supplementary 4–7). When stratified by 14 mmHg, a significant difference was observed between the two groups for rebleeding (39.12 vs. 51.22%, *p* = 0.006, HR = 1.489, 95% CI 1.120–1.979) before PSM, but not in OHE and mortality (Fig. 2 and Supplementary Fig. 8–9). Decision curve analysis (DCA) showed that using 12 mmHg as a cut-off in terms of rebleeding could achieve more net benefit than using 14 mmHg (Fig. 5).

### The effect of PPG reduction on clinical outcomes in patients with different liver function categories

#### *PPG* < *12 mmHg in different liver function categories*

As shown in Fig. 1, the outcomes of different Child–Pugh class patients were significantly different. Therefore, we further analyzed the PPG thresholds in different Child–Pugh classes.

In patients graded Child–Pugh A, setting 12 mmHg as the cutoff value for post-TIPS PPG was not discriminative of rebleeding, OHE, or mortality before or after PSM (Supplementary Fig. 10). Competing risk analysis also showed consistent results (Supplementary Fig. 11).

**Fig. 3:**
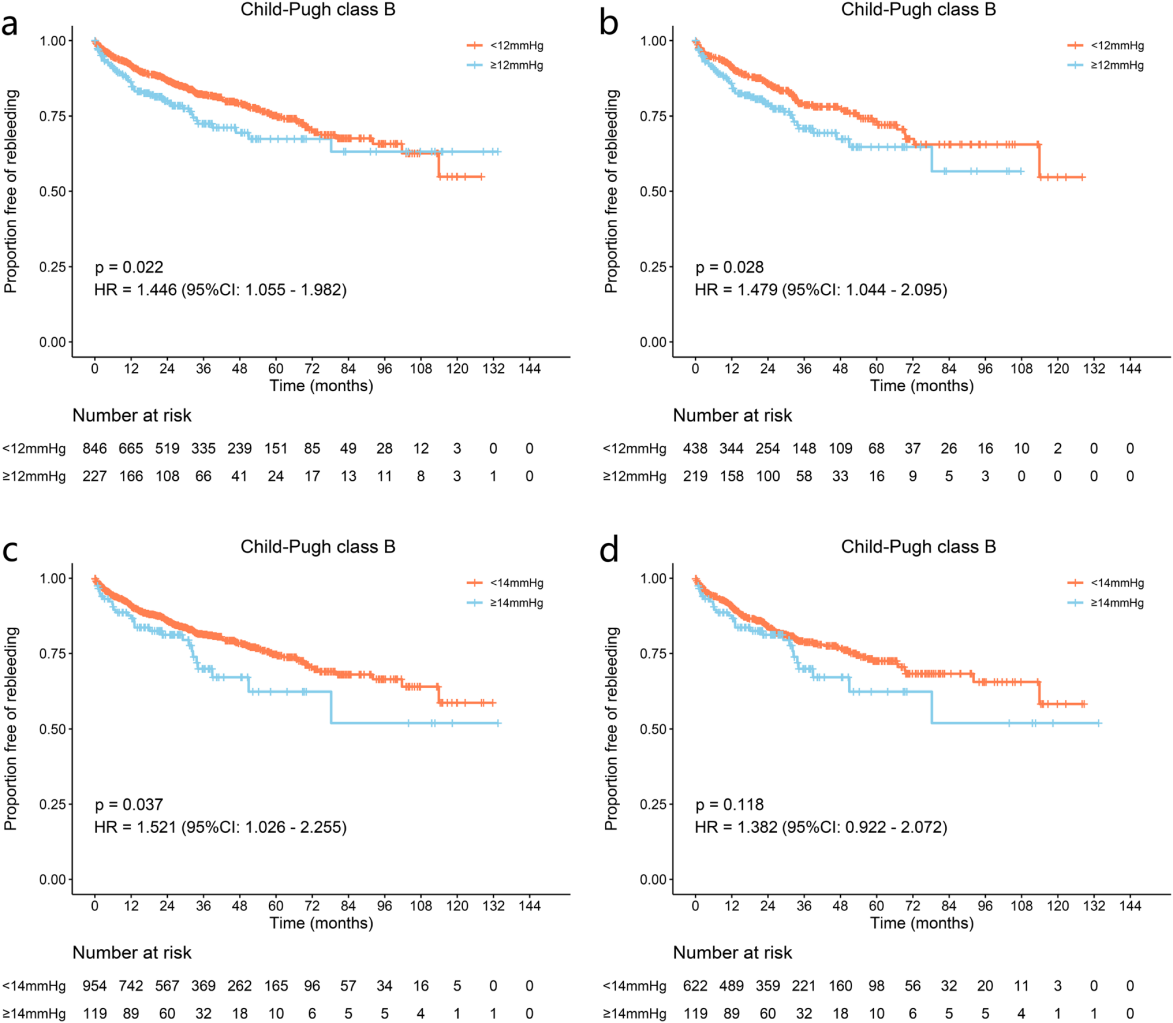
Proportion of Child–Pugh class B patients without rebleeding with a 12 or 14 mmHg PPG threshold. The proportion of Child–Pugh class B patients without rebleeding **a** before and **b** after PSM with a 12 mmHg PPG threshold. The proportion of Child–Pugh class B patients without rebleeding **c** before and **d** after PSM with a 14 mmHg PPG threshold

In patients with Child–Pugh B, significant differences were observed between the PPG < 12 mmHg and PPG ≥ 12 mmHg groups for rebleeding (45.18 vs. 36.79%, *p* = 0.022, HR = 1.446, 95% CI 1.055–1.982), but not for OHE and mortality (*p* = 0.183, HR = 0.836, 95% CI 0.642–1.088 and *p* = 0.925, HR = 1.013, 95% CI 0.774–1.326, respectively). After 1:2 PSM, PPG < 12 mmHg group showed reduced rebleeding compared with PPG ≥ 12 mmHg group (45.33 vs. 43.32%, *p* = 0.028, HR = 1.479, 95% CI 1.044–2.095), but presented similar OHE incidence and mortality (*p* = 0.089, HR = 0.779, 95% CI: 0.584–1.039 and *p* = 0.146, HR = 1.247, 95% CI 0.926–1.679, respectively) (Fig. 3 and Supplementary Fig. 12). The competing risk analysis confirmed these results (Supplementary Fig. 13).

**Fig. 4:**
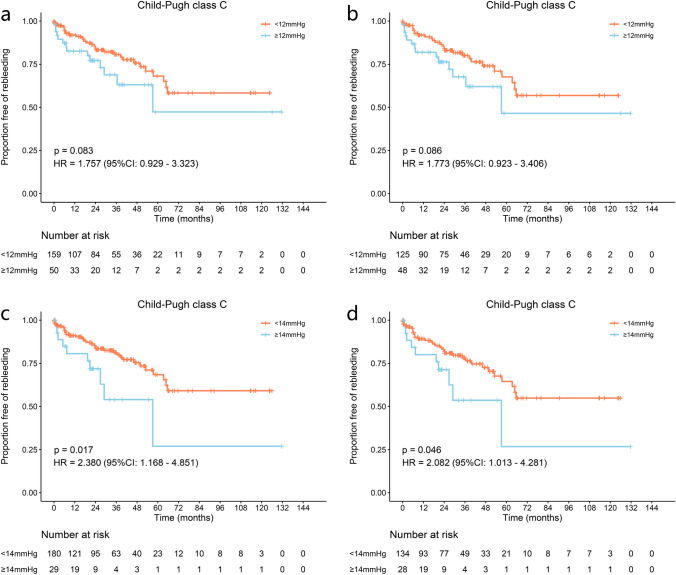
Proportion of Child–Pugh class C patients without rebleeding with a 12 or 14 mmHg PPG threshold. **a** Proportion free of rebleeding in all patients with 12 mmHg threshold before PSM. **b** Proportion free of rebleeding in all patients with 12 mmHg threshold after PSM. **c** Proportion free of rebleeding in all patients with 14 mmHg threshold before PSM. **d** Proportion free of rebleeding in all patients with 14 mmHg threshold after PSM

In class C patients, no significant difference was detected in rebleeding, OHE, and mortality either before or after 1:3 PSM between the two groups (Fig. 4 and Supplementary Fig. 14). Competing risk analysis, however, showed significant differences in rebleeding before PSM (43.14 vs. 53.50%, *p* = 0.032, HR = 1.913, 95% CI = 1.018–3.595), even though OHE incidence and mortality were similar (*p* = 0.280, HR = 0.768, 95% CI 0.479–1.231 and *p* = 0.062, HR = 0.610, 95% CI 0.363–1.023, respectively; Supplementary Fig. 15).

#### Other PPG thresholds of 8, 10, and 14 mmHg in different liver function categories

None of the tested PPG thresholds (8, 10, and 14 mmHg) showed significant differences in rebleeding, OHE, or mortality in patients graded as Child–Pugh class A (Supplementary Fig. 16–21).

In the Child–Pugh class B, PPG < 8 and 10 mmHg could not reduce rebleeding either before or after PSM (Supplementary Fig. 22–25). Although PPG < 14 mm Hg showed reduced rebleeding (41.33 vs 48.06%, *p* = 0.037, HR = 1.521, 95% CI 1.026–2.255), no improvement regarding OHE occurrence or mortality was noted (*p* = 0.064, HR = 0.706, 95% CI 0.489–1.020 and *p* = 0.239, HR = 1.225, 95% CI 0.874–1.718, respectively). After a 1:6 PSM, there were no significant differences in rebleeding, OHE, or mortality. (Fig. 3 and Supplementary Fig. 26) Similarly, competing risk analysis showed no significant difference in any outcome (Supplementary Fig. 27).

In Child–Pugh class C, no significant difference was observed in rebleeding, OHE, or mortality between groups when stratified by 8 mmHg or 10 mmHg (Supplementary Fig. 28–31). PPG < 14 mmHg could reduce rebleeding (40.92 vs 73.04%, *p* = 0.017, HR = 2.380, 95%CI 1.168–4.851), but failed to show statistically different OHE incidence or mortality (*p* = 0.216, HR = 0.674, 95% CI 0.360–1.260 and *p* = 0.351, HR = 0.733, 95% CI 0.381–1.409, respectively). After 1:5 PSM, the significance of between-group differences regarding rebleeding remained robust (45.17 vs. 73.23%, *p* = 0.046, HR = 2.082, 95% CI 1.013–4.281), and the lack of significant differences in OHE and mortality also persisted (*p* = 0.423, HR = 0.760, 95% CI 0.388–1.487 and *p* = 0.678, HR = 0.868, 95%CI 0.445–1.694, respectively) (Fig. 4 and Supplementary Fig. 32). Competing risk analysis showed similar results for these outcomes (Supplementary Fig. 33).

**Fig.5:**
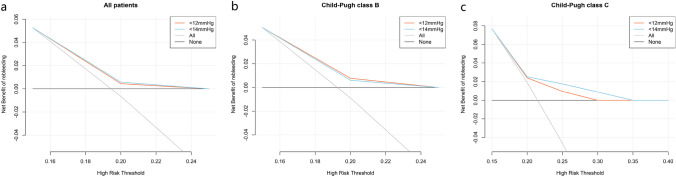
Decision curve analysis of rebleeding in Child–Pugh class B and C patients with 12 and 14 mmHg PPG thresholds. DCAs of rebleeding in **a** all patients, **b** Child–Pugh class B patients, and **c** Child–Pugh class C patients with PPG thresholds of 12 and 14 mmHg

Therefore, as both 12 and 14 mmHg could reduce rebleeding in Child–Pugh class B and C patients, we compared their net benefits in rebleeding by DCA to determine the optimal threshold. DCA showed that 12 mmHg was marginally better than 14 mmHg in patients graded Child–Pugh class B, while 14 mmHg was superior to 12 mmHg in patients graded Child–Pugh class C (Fig. 5).

### Alternative standards (PPG reduction rate)

We also dichotomized patients with PPG reductions of 30, 40, and 50%; none which were statistically discriminative of rebleeding, OHE, or mortality (Supplementary Fig. 34).

## Discussion

This study confirms the prognostic value of PPG on patient outcomes, including rebleeding and OHE, the study revealed the necessity of tailoring PPG thresholds according to liver function, and identifies the optimal PPG thresholds in patients with different Child–Pugh grades. The advantages of the current study are as follows: (1) being conducted on the largest real-world TIPS cohort to date; (2) updating TIPS threshold in the era of covered stents with lower shunt dysfunction rate and possibly more stable post-procedure PPG variation; (3) applying multiple statistical methods including PSM and competing risk to validate the robustness of our findings; and (4) all the patients enrolled in this study underwent TIPS for secondary prophylaxis of rebleeding under local anesthesia, which avoids the influence of emergency vasoconstrictive drugs and sedation on PPG measurement. Therefore, immediate PPG can represent the real situation of PV pressure in this study.

A target threshold of 12 mmHg was proposed and implemented over 30 years ago [4, 5, 8, 9, 13, 21–24], and has since been recommended by Baveno VII [1], derived from uncoated TIPS. However, this has not yet been confirmed for covered TIPS. Therefore, it is important to explore the PPG threshold suitable for covered-stent TIPS. Moreover, the optimal PPG threshold should be tailored according to Child–Pugh classes because patients with different liver function classes have different PPG reduction tolerances.

Bosch et al. hypothesized that higher PPGs, such as 14 mmHg, might be more appropriate for covered stents than lower PPGs since their well-maintained patency would reduce the need to immediately lower post-TIPS PPG, which is required to counter gradually reduced shunt diameter and increased PPG with bare stents [13]. A recent cohort study of 216 patients confirmed this hypothesis, the study found that post-procedure PPGs higher than 12 mmHg did not necessarily indicate higher variceal rebleeding rates after 12 months [25]. Therefore, we explored this issue using a larger TIPS cohort.

In this study, patients with PPGs > 12 mmHg after TIPS had a higher incidence of rebleeding than those with PPGs < 12 mmHg. However, this result was not robust after PSM. PPGs > 14 mmHg also had a higher incidence of rebleeding than PPGs < 14 mmHg, but provided a net benefit higher than that of > 12 mmHg. However, this result was not robust after PSM. Nevertheless, when the entire cohort was stratified according to Child–Pugh class, an indicator of liver function, we found that 12 mmHg did not affect rebleeding or OHE rates in Child–Pugh class A patients. However, 12 mmHg affected rebleeding in Child–Pugh class B and C patients. These results indicate that a one-size-fits-all strategy may not be suitable for setting a PPG threshold, with liver function being important in tailoring PPG targets for different patients [26, 27]. Therefore, we explored several PPG thresholds to propose an appropriate threshold for each Child–Pugh class.

Neither 10 nor 14 mmHg PPG affected rebleeding or OHE after TIPS in Child–Pugh class A patients, perhaps reflecting their relatively low cumulative incidence, increasing the difficulty in identifying significant differences. However, the results showed that patients had numerically lower rebleeding rates with PPGs < 10 mmHg than those with PPGs > 10 mmHg, which was not observed at PPG thresholds of 12 and 14 mmHg, indicating that 10 mmHg might be a more appropriate threshold than 12 and 14 mmHg. Attia et al. reported that patients with lower Child–Pugh scores could tolerate lower PPGs than those with higher Child–Pugh scores [27]. Therefore, we hypothesized that a PPG threshold of < 10 mmHg might be suitable for patients with Child–Pugh class A. Unfortunately, the small number of patients with PPG values in our cohort did not allow powered statistical tests.

A PPG threshold of 12 mmHg, but not 14 mmHg, affected rebleeding and OHE rates in Child–Pugh class B patients, indicating that a PPG threshold of 12 mmHg remains appropriate for them. In contrast, a PPG threshold of 14 mmHg appeared to be effective in reducing the rebleeding risk in Child–Pugh class C patients. These patients had a high incidence of OHE (> 50%), which we believe causes the lack of a significant OHE effect. Furthermore, a lower PPG threshold generally indicates more shunting, which might further reduce liver perfusion and exacerbate liver damage based on severely impaired function [6, 28–30]. Therefore, we hypothesized that a PPG of < 14 mmHg is better than a PPG of < 12 mmHg for Child–Pugh class C patients.

Although the percentage of pressure reduction is an important rebleeding indicator, several studies have explored its effective range [5–9, 31]. In this study, post-TIPS PPGs remained high in patients with high pre-TIPS PPGs (1065 had pre-TIPS PPGs ≥ 24 mmHg), even when the reduction exceeded a certain percentage (e.g., a 50% reduction of 40 mmHg was 20 mmHg). Consequently, these patients were still exposed to adverse outcomes, and meaningful results could not be obtained.

Our study has several limitations. First, it was a retrospective study with incomplete information on post-TIPS liver function and the causes of rebleeding or death. However, we included consecutive patients to avoid excessive bias. Second, to date, the PPG threshold of < 12 mmHg has been the goal of TIPS. Therefore, only approximately 16% of patients in this study had post-TIPS PPG ≥ 12 mmHg, increasing the difficulty of identifying significant differences in PSM analyses when the sample size was further reduced.

In conclusion, a PPG threshold < 12 mmHg after TIPS may not be optimal for every patient. Different PPG thresholds may be required in patients with different liver function categories. Moreover, although a PPG threshold of < 12 mmHg may be suitable for Child–Pugh class B patients, a PPG threshold of < 14 mmHg is more suitable for Child–Pugh class C patients. Given the retrospective nature of this study, these results should be confirmed in future prospective studies with larger cohorts.

## Supplementary Information

Below is the link to the electronic supplementary material.

Supplementary file1 (DOCX 15 KB)

Supplementary file2 (PDF 24672 KB)

## Data Availability

All data collection was approved by Research Ethics Committee of participating center. The data are not publicly available due to privacy and ethical restrictions.

## Abbreviations

CI: Confidence interval;
DCA: Decision curve analysis;
HE: Hepatic encephalopathy;
HR: Hazard ratios;
MELD: Model for end-stage liver disease;
NSBB: Non-selective beta-blockers;
OHE: Overt hepatic encephalopathy;
PSM: Propensity score matching;
PPG: Portal pressure gradient;
PT: Prothrombin time;
SMD: Standard mean difference;
TIPS: Transjugular intrahepatic portosystemic shunt
