# Supplementary material for: Individualized portal pressure gradient threshold based on liver function categories in preventing rebleeding after TIPS
Source: Hepatol Int. 2023 Feb 17;17(4):967–78. doi: 10.1007/s12072-023-10489-x (PMC10386972; doi:10.1007/s12072-023-10489-x)
Supplement: Supplementary file 1 — Supplementary file1 (DOCX 15 KB) [file 12072_2023_10489_MOESM1_ESM.docx]

|  | 6mm | 8mm | 10mm | P value |
| --- | --- | --- | --- | --- |
| Post-TIPS PPG (mmHg) | 10.0 (8.0, 13.0) | 8.6 (6.0, 11.0) | 8.1 (5.2, 10.3) | <0.001 |
| PPG decrease rate (%) | 52.2 (36.8, 62.5) | 64.1 (53.3, 73.0) | 70.1 (59.4, 77.6) | <0.001 |

Supplementary table 18 Portal pressure according to stent diameters.

PPG, portal pressure gradient.
