# Supplementary material for: Individualized portal pressure gradient threshold based on liver function categories in preventing rebleeding after TIPS
Source: Hepatol Int. 2023 Feb 17;17(4):967–78. doi: 10.1007/s12072-023-10489-x (PMC10386972; doi:10.1007/s12072-023-10489-x)
Supplement: Supplementary file 2 — Supplementary file2 (PDF 24672 KB) [file 12072_2023_10489_MOESM2_ESM.pdf]

Supplementary Tables and Figures

Title:

Individualized Portal Pressure Gradient Threshold Based on Liver Function Categories in Preventing Rebleeding After TIPS

Journal name:

Hepatology International

Authors:

Yifu Xia1, #, Jun Tie2, #, Guangchuan Wang3, 4, #, Yuzheng Zhuge5, Hao Wu6, Hui Xue7, Jiao Xu2, Feng Zhang5, Lianhui Zhao1, 3, Guangjun Huang3, Mingyan Zhang3, Bo Wei6, Peijie Li7, Wei Wu8, Chao Chen8, Chengwei Tang6, Chunqing Zhang1, 3

# Yifu Xia, Jun Tie and Guangchuan Wang have contributed equally as first authors.

Corresponding author:

Professor Chunqing Zhang, Department of Gastroenterology, Provincial Hospital Affiliated to Shandong First Medical University, 324 Jing Wu Wei Qi Road, Jinan 250021, Shandong Province, China;

Phone: +8653168773293;

Fax: +8653187906348;

Email: zhangchunqing_sdu@163.com.

And

Professor Chengwei Tang, Department of Gastroenterology and Hepatology, West China Hospital, No.37 Guoxue Alley Wuhou District, Chengdu 610041, Sichuan Province, China.

Phone: +8602885422114;

Fax: +8602885582944;

Email: shcqcdmed@163.com.

Supplementary Table 1 Univariable and multivariable analysis in all patients.

Characteristics	Univariable analysis				Multivariable analysis			
	β	HR	95%CI	P value	β	HR	95%CI	P value
**Rebleeding**								
Age (per year increase)	-0.003	0.997	(0.988,1.005)	0.475				
Sex (male vs female)	0.564	1.758	(1.402,2.204)	<0.001	0.589	1.801	(1.436,2.260)	<0.001
Etiology of cirrhosis (virus or not)	-0.119	0.888	(0.715,1.103)	0.282				
Platelet (per 10^9/L increase)	-0.001	0.999	(0.997,1.000)	0.120				
Hemoglobin (per g/L increase)	-0.010	0.990	(0.985,0.995)	<0.001	-0.009	0.991	(0.985,0.996)	<0.001
Albumin (per g/L increase)	-0.014	0.986	(0.970,1.003)	0.118				
Total bilirubin (per µmol/L increase)	0.003	1.003	(0.996,1.011)	0.357				
Creatinine (per µmol/L increase)	0.002	1.002	(0.997,1.006)	0.402				
Sodium (per mmol/L increase)	-0.010	0.990	(0.971,1.011)	0.351				
PT prolong (per second increase)	0.004	1.004	(0.982,1.027)	0.702				
International normalized ratio (per unit increase)	0.000	1.000	(0.974,1.027)	0.974				

Child-Pugh score (per point increase)	0.062	1.064	(1.004,1.127)	0.037				
Child-Pugh class				0.092				
A VS B	0.128	1.137	(0.924,1.399)	0.227				
A VS C	0.361	1.435	(1.030,2.000)	0.033				
B VS C	0.233	1.262	(0.914,1.743)	0.157				
MELD score (per point increase)	0.024	1.025	(0.999,1.051)	0.057				
MELD-Na score (per point increase)	0.011	1.011	(0.993,1.030)	0.236				
Ascites before TIPS (yes or no)	-0.070	0.932	(0.760,1.144)	0.502				
HE before TIPS (yes or no)	0.501	1.650	(0.411,6.623)	0.480				
Red-color sign (yes or no)	0.161	1.174	(0.918,1.502)	0.202				
Diameter (mm)	-0.249	0.780	(0.644,0.945)	0.011				
Stent diameter groups (6-7mm vs 8mm vs 10mm)				<0.001				<0.001
6-7mm vs 8mm	-0.936	0.392	(0.258,0.596)	<0.001	-0.938	0.391	(0.257,0.596)	<0.001
6-7mm vs 10mm	-0.970	0.379	(0.212,0.677)	0.001	-0.975	0.377	(0.211,0.675)	0.001

8mm vs 10mm	-0.034	0.966	(0.633,1.476)	0.875	-0.037	0.963	(0.630,1.472)	0.863
Pre-TIPS PPG (per mmHg increase)	-0.003	0.997	(0.980,1.014)	0.720				
After-TIPS PPG (per mmHg increase)	0.038	1.038	(1.013,1.064)	0.003				
PPG decrease rates (per percentage increase)	-0.762	0.467	(0.267,0.817)	0.008				
**OHE**								
Age (per year increase)	0.026	1.026	(1.019,1.033)	<0.001	0.026	1.027	(1.020,1.034)	<0.001
Sex (male vs female)	0.075	1.078	(0.923,1.260)	0.341				
Etiology of cirrhosis (virus or not)	-0.060	0.942	(0.803,1.105)	0.463				
Platelet (per 10^9/L increase)	0.000	1.000	(0.999,1.001)	0.816				
Hemoglobin (per g/L increase)	0.005	1.005	(1.001,1.008)	0.013	0.006	1.006	(1.002,1.010)	0.002
Albumin (per g/L increase)	-0.030	0.970	(0.957,0.983)	<0.001				
Total bilirubin (per µmol/L increase)	0.007	1.007	(1.002,1.012)	0.009				
Creatinine (per µmol/L increase)	0.008	1.008	(1.005,1.011)	<0.001	0.007	1.007	(1.004,1.010)	<0.001

Sodium (per mmol/L increase)	-0.004	0.996	(0.981,1.012)	0.633				
PT prolong (per second increase)	0.018	1.018	(1.010,1.027)	<0.001				
International normalized ratio (per unit increase)	0.007	1.007	(0.997,1.016)	0.185				
Child-Pugh score (per point increase)	0.147	1.158	(1.110,1.209)	<0.001				
Child-Pugh class				<0.001				<0.001
A VS B	0.239	1.270	(1.078,1.496)	0.004	0.271	1.311	(1.109,1.550)	0.002
A VS C	0.751	2.118	(1.680,2.671)	<0.001	0.833	2.300	(1.815,2.914)	<0.001
B VS C	0.512	1.669	(1.341,2.077)	<0.001	0.562	1.754	(1.407,2.187)	<0.001
MELD score (per point increase)	0.037	1.038	(1.022,1.055)	<0.001				
MELD-Na score (per point increase)	0.020	1.020	(1.007,1.034)	0.002				
Ascites before TIPS (yes or no)	-0.302	0.739	(0.627,0.871)	<0.001				
HE before TIPS (yes or no)	-0.699	0.497	(0.274,0.902)	0.022				
Red-color sign (yes or no)	0.042	1.043	(0.860,1.264)	0.670				
Diameter (mm)	0.211	1.235	(1.088,1.402)	0.001				

Stent diameter groups (6-7mm vs 8mm vs 10mm)				0.002				0.001
6-7mm vs 8mm	0.103	1.108	(0.748,1.642)	0.607	0.037	1.038	(0.694,1.553)	0.857
6-7mm vs 10mm	0.623	1.864	(1.159,2.998)	0.010	0.584	1.794	(1.097,2.934)	0.020
8mm vs 10mm	0.520	0.902	(0.609,1.336)	<0.001	0.547	1.728	(1.291,2.314)	<0.001
Pre-TIPS PPG (per mmHg increase)	0.006	1.006	(0.993,1.019)	0.342				
After-TIPS PPG (per mmHg increase)	-0.031	0.969	(0.950,0.989)	0.002				
PPG decrease rates (per percentage increase)	0.884	2.419	(1.515,3.864)	<0.001	0.645	1.905	(1.184,3.064)	0.008
**Mortality**								
Age (per year increase)	0.033	1.034	(1.027,1.041)	<0.001	0.034	1.035	(1.028,1.042)	<0.001
Sex (male vs female)	-0.107	0.899	(0.765,1.056)	0.195				
Etiology of cirrhosis (virus or not)	0.159	1.173	(0.993,1.385)	0.061				
Platelet (per 10^9/L increase)	-0.001	0.999	(0.998,1.000)	0.103				
Hemoglobin (per g/L increase)	0.001	1.001	(0.997,1.005)	0.606				

Albumin (per g/L increase)	-0.043	0.958	(0.945,0.971)	<0.001				
Total bilirubin (per μmol/L increase)	0.020	1.021	(1.016,1.026)	<0.001	0.010	1.010	(1.004,1.016)	0.001
Creatinine (per μmol/L increase)	0.004	1.004	(1.000,1.008)	0.027				
Sodium (per mmol/L increase)	-0.030	0.971	(0.956,0.985)	<0.001				
PT prolong (per second increase)	0.021	1.022	(1.011,1.032)	<0.001				
International normalized ratio (per unit increase)	0.010	1.010	(1.000,1.021)	0.061				
Child-Pugh score (per point increase)	0.205	1.228	(1.176,1.281)	<0.001				
Child-Pugh class				<0.001				<0.001
A VS B	0.503	1.654	(1.386,1.974)	<0.001	0.381	1.464	(1.219,1.758)	<0.001
A VS C	0.994	2.701	(2.123,3.436)	<0.001	0.651	1.918	(1.432,2.569)	<0.001
B VS C	0.490	1.633	(1.309,2.037)	<0.001	0.271	1.311	(1.019,1.666)	0.035
MELD score (per point increase)	0.056	1.058	(1.041,1.075)	<0.001	0.027	1.027	(1.007,1.048)	0.008
MELD-Na score (per point increase)	0.039	1.040	(1.027,1.054)	<0.001				

Ascites before TIPS (yes or no)	-0.468	0.626	(0.526,0.745)	<0.001				
HE before TIPS (yes or no)	-0.323	0.724	(0.344,1.525)	0.396				
Red-color sign (yes or no)	0.008	1.008	(0.823,1.235)	0.938				
Diameter (mm)	0.137	1.147	(1.008,1.305)	0.037				
stent diameter groups (6-7mm vs 8mm vs 10mm)				0.033				0.002
6-7mm vs 8mm	-0.220	0.803	(0.487,1.325)	0.390	-0.051	0.950	(0.574,1.572)	0.843
6-7mm vs 10mm	0.126	1.134	(0.648,1.985)	0.660	0.449	1.567	(0.891,2.756)	0.119
8mm vs 10mm	0.345	1.412	(1.077,1.852)	0.012	0.500	1.052	(0.636,1.741)	0.843
Pre-TIPS PPG (per mmHg increase)	0.011	1.011	(0.997,1.025)	0.119				
After-TIPS PPG (per mmHg increase)	-0.010	0.990	(0.969,1.011)	0.361				
PPG decrease rates (per percentage increase)	0.526	1.692	(1.020,2.806)	0.042				

Supplementary Table 2 PSM for 8mmHg in all patients.

Characteristics	Before PSM			After PSM			
	<8mmHg (n=821)	≥8mmHg (n=1279)	P value	<8mmHg (n=818)	≥8mmHg (n=818)	P value	SMD
Age (per year increase)	54(46,62)	51(43,59)	<0.001	54(46,62)	53(46,61.5)	0.824	0.001
Sex (n, male/female)	461/360	890/389	<0.001	459/359	479/339	0.317	0.049
Etiology of cirrhosis (n, %)			0.109			0.876	0.008
Virus	536(65.3)	878(68.6)		533(65.2)	536(65.5)		
Others	285(34.7)	401(31.4)		285(34.8)	282(34.5)		
Platelet (per 10^9/L increase)	65(44,99)	56(40,82)	<0.001	65(44,99)	65(44,91.5)	0.383	0.074
Hemoglobin	81(69,96)	81(70,94)	0.516	81(69,96)	82(70,95)	0.709	0.028
Albumin (per g/L increase)	33.9(30.3,37.5)	33.6(30.1,37.225)	0.675	33.9(30.3,37.55)	33.7(30.2,37.4)	0.78	0.018
Total bilirubin (per g/L increase)	20.05(13.7,29.5)	21.1(14.8,30.725)	0.015	20.1(13.7,29.55)	20.8(14.55,29.95)	0.137	0.053
Creatinine (per µmol/L increase)	75(61,90)	71(57.925,86)	<0.001	75(61,90)	74(59,90)	0.472	0.002
Sodium (per mmol/L increase)	139.7(137.3,142)	139.8(137.1,142)	0.572	139.7(137.3,142)	139.6(136.9,142)	0.3	0.075

PT prolong (per second increase)	1.3(0,3.2)	1.7(0.2,3.425)	0.001	1.3(0,3.2)	1.6(0.025,3.3)	0.056	0.065
International normalized ratio (per unit increase)	1.29(1.17,1.44)	1.31(1.2,1.48)	0.004	1.29(1.17,1.445)	1.31(1.19,1.47)	0.074	0.05
Child-Pugh score (per point increase)	7(6,8)	7(6,8)	0.048	7(6,8)	7(6,8)	0.087	0.077
Child-Pugh class (n, %)			0.334			0.449	0.063
class A	335(40.8)	483(37.8)		333(40.7)	308(37.7)		
class B	410(49.9)	663(51.8)		409(50.0)	430(52.6)		
class C	76(9.3)	133(10.4)		76(9.3)	80(9.8)		
MELD score (per point increase)	10(9,13)	11(9,13)	0.131	10(9,13)	11(9,13)	0.3	0.035
MELD-Na score (per point increase)	11(9,13)	11(9,14)	0.287	11(9,13)	11(9,14)	0.354	0.052
Ascites before TIPS (n, %)	527(64.2)	886(69.3)	0.015	526(64.3)	570(69.7)	0.021	0.115
HE before TIPS (n, %)	8(1.0)	14(1.1)	0.792	8(1.0)	8(1.0)	1	<0.001
Red-color sign (n, %)	537(76.6)	796(79.9)	0.101	536(65.5)	521(63.7)		0.049

Diameter (mm)	8(8,8)	8(8,8)	<0.001	8(8,8)	8(8,8)	0.528	0.021
Stent diameter groups			<0.001			0.141	0.098
6-7mm	18(2.2)	77(6.0)		18(2.2)	31(3.8)		
8mm	756(92.1)	1148(89.8)		754(92.2)	736(90.0)		
10mm	47(5.7)	54(4.2)		46(5.6)	51(6.2)		

PSM, propensity score match; PPG, portal pressure gradient; TIPS, transjugular intrahepatic portosystemic shunt.

1:1 PSM with age, sex, stent diameter, platelet, creatinine before TIPS. Caliper value was 0.4.

Supplementary Table 3 PSM for 10mmHg in all patients.

Characteristics	Before PSM <10mmHg (n=1303)	Before PSM ≥10mmHg (n=797)	P value	After PSM <10mmHg (n=796)	After PSM ≥10mmHg (n=796)	P value	SMD
Age (per year increase)	53(45,61)	50(42,58)	<0.001	50(43,59)	50(42,58)	0.81	0.01
Sex (n, male/female)	775/528	576/221	<0.001	585/211	575/221	0.573	0.03
Etiology of cirrhosis (n, %)			0.423			0.155	0.07
Virus	869(66.7)	545(68.4)		570(71.6)	544(68.3)		
Others	434(33.3)	252(31.6)		226(28.4)	252(31.7)		
Platelet (per 10^9/L increase)	60(42,90)	56(40,83)	0.004	60(42,88)	56(40,83)	0.062	0.09
Hemoglobin	82(70,96)	79(69,94)	0.026	81(69,95)	79(69,94)	0.241	0.05
Albumin (per g/L increase)	34(30.4,37.6)	33.4(30,37.1)	0.107	33.2(29.9,36.7)	33.4(29.975,37.1)	0.441	0.02
Total bilirubin (per g/L increase)	20.6(13.9,29.2)	21.3(15.3,31.6)	0.003	20.9(13.9,30.4)	21.35(15.3,31.625)	0.045	0.1
Creatinine (per µmol/L increase)	74(60,89)	71(57,86)	<0.001	72(59,87)	71(57,86)	0.036	0.07
Sodium (per mmol/L increase)	139.8(137.4,142)	139.7(137,142)	0.29	139.6(137.15,141.8)	139.7(137,142)	0.9413	0

PT prolong (per second increase)	1.4(0,3.2)	1.8(0.1,3.6)	0.005	1.6(0.2,3.5)	1.8(0.1,3.6)	0.448	0.06
International normalized ratio (per unit increase)	1.3(1.18,1.45)	1.32(1.2,1.48)	0.009	1.31(1.19,1.48)	1.32(1.2,1.48)	0.36	0.05
Child-Pugh score (per point increase)	7(6,8)	7(6,8)	<0.001	7(6,8)	7(6,8)	0.435	0.03
Child-Pugh class (n, %)			0.012			0.921	0.02
class A	539(41.4)	279(35.0)		282(35.4)	279(35.1)		
class B	644(49.4)	429(53.8)		430(54.0)	428(53.8)		
class C	120(9.2)	89(11.2)		84(10.6)	89(11.2)		
MELD score (per point increase)	10(9,13)	11(9,13)	0.021	10(9,13)	11(9,13)	0.096	0.03
MELD-Na score (per point increase)	11(9,13)	11(9,14)	0.018	11(9,13)	11(9,14)	0.136	0.03
Ascites before TIPS (n, %)	850(65.2)	563(70.6)	0.01	546(68.6)	562(70.6)	0.383	0.04
HE before TIPS (n, %)	11(0.8)	11(1.4)	0.242	7(0.9)	11(1.4)	0.343	0.05
Red-color sign (n, %)	849(77.6)	484(80.3)	0.201	516(64.8)	484(60.8)		0.03

Diameter (mm)	8(8,8)	8(8,8)	<0.001	8(8,8)	8(8,8)	0.254	0.04
Stent diameter groups			<0.001			0.208	0.09
6-7mm	41(3.1)	54(6.8)		37(4.6)	53(6.7)		
8mm	1191(91.4)	713(89.5)		731(91.8)	713(89.6)		
10mm	71(5.4)	30(3.8)		28(3.5)	30(3.8)		

PSM, propensity score match; PPG, portal pressure gradient; TIPS, transjugular intrahepatic portosystemic shunt.

1:1 PSM with age, sex, stent diameter, Child-Pugh score, MELD score, creatinine before TIPS. Caliper value was 0.4.

Supplementary Table 4 PSM for 12mmHg in all patients.

Characteristics	Before PSM <12mmHg (n=1682)	Before PSM ≥12mmHg (n=418)	P value	After PSM <12mmHg (n=830)	After PSM ≥12mmHg (n=418)	P value	SMD
Age (per year increase)	52(45,60.75)	50(43,58)	0.008	51(43,59)	50(43,58)	0.753	0.012
Sex (n, male/female)	1038/644	313/105	<0.001	612/218	313/105	0.663	0.026
Etiology of cirrhosis (n, %)			0.042			0.005	0.167
Virus	1150(68.4)	264(63.2)		589(71.0)	264(63.2)		
Others	532(31.6)	154(36.8)		241(29.0)	154(36.8)		
Platelet (per 10^9/L increase)	60(42,89)	56(40,83.25)	0.052	59(41,87.25)	56(40,83.25)	0.183	0.082
Hemoglobin	82(70,96)	78(68,93)	0.003	80(69,92)	78(68,93)	0.309	0.03
Albumin (per g/L increase)	33.9(30.3,37.5)	33.25(29.9,36.9)	0.097	33(29.6,36.925)	33.25(29.9,36.9)	0.592	0.026
Total bilirubin (per g/L increase)	20.9(14.125,29.9)	21(15.075,31.025)	0.166	21.45(14.1,31.3)	21(15.075,31.025)	0.623	0.022
Creatinine (per µmol/L increase)	74(60,89)	67(54.955,81.25)	<0.001	69(56,82)	67(54.955,81.25)	0.205	0.05
Sodium (per mmol/L increase)	139.7(137.3,142)	139.8(136.975,142)	0.755	139.65(137.2,141.9)	139.8(136.975,142)	0.964	0.029

PT prolong (per second increase)	1.5(0,3.3)	1.9(0.2,3.6)	0.051	1.6(0.05,3.6)	1.9(0.2,3.6)	0.477	0.062
International normalized ratio (per unit increase)	1.3(1.18,1.46)	1.32(1.2075,1.49)	0.03	1.31(1.19,1.47)	1.32(1.2075,1.49)	0.213	0.042
Child-Pugh score (per point increase)	7(6,8)	7(6,8)	0.002	7(6,9)	7(6,8)	0.597	0.044
Child-Pugh class (n, %)			0.033			0.793	0.041
class A	677(39.0)	141(33.7)		267(32.2)	141(33.7)		
class B	846(50.3)	227(543.3)		455(54.8)	227(54.3)		
class C	159(9.5)	50(12.0)		108(13.0)	50(12.0)		
MELD score (per point increase)	11(9,13)	10(9,13)	0.613	11(9,13)	10(9,13)	0.616	0.061
MELD-Na score (per point increase)	11(9,13)	11(9,14)	0.785	11(9,13)	11(9,14)	0.859	0.043
Ascites before TIPS (n, %)	1108(65.9)	305(73.0)	0.006	616(74.2)	305(73.0)	0.635	0.028
HE before TIPS (n, %)	15(0.9)	7(1.7)	0.159	10(1.2)	7(1.7)	0.499	0.039
Red-color sign (n, %)	1095(78.1)	238(80.7)	0.327	520(79.3)	238(80.7)	0.617	0.035

Diameter (mm)			0.845			0.918	
Stent diameter groups	259(16.5)	52(17.0)		127(16.7)	52(17.0)		
6-7mm	1307(83.5)	254(83.0)		632(83.3)	254(83.0)		
8mm	8(8,8)	8(8,8)	<0.001	8(8,8)	8(8,8)	0.529	0.01
10mm			<0.001			0.266	0.096

PSM, propensity score match; PPG, portal pressure gradient; TIPS, transjugular intrahepatic portosystemic shunt.

1:2 PSM with age, sex, stent diameter, Child-Pugh score, ascites, creatinine before TIPS. Caliper value was 0.4.

Supplementary Table 5 PSM for 14mmHg in all patients.

Characteristics	Before PSM <14mmHg (n=1873)	Before PSM ≥14mmHg (n=227)	P value	After PSM <14mmHg (n=864)	After PSM ≥14mmHg (n=227)	P value	SMD
Age (per year increase)	52(44,60)	50(42,59)	0.014	49(42,58)	50(42,59)	0.931	0.019
Sex (n, male/female)	1180/693	171/56	<0.001	655/209	171/56	0.881	0.011
Etiology of cirrhosis (n, %)			0.054			0.012	0.184
Virus	1274(68.0)	140(61.7)		608(70.4)	140(61.7)		
Others	599(32.0)	87(38.3)		256(29.6)	87(38.3)		
Platelet (per 10^9/L increase)	60(42,89)	55(39,76)	0.009	55(39,84)	55(39,76)	0.462	0.031
Hemoglobin	81(70,95)	76(68,93)	0.008	80(69,95)	76(68,93)	0.099	0.108
Albumin (per g/L increase)	33.8(30.2,37.5)	33.2(30.4,36.5)	0.173	33(29.875,36.8)	33.2(30.4,36.5)	0.803	0.014
Total bilirubin (per g/L increase)	20.9(14.3,30.1)	21(14.8,30.5)	0.424	22.45(15.1,32.2)	21(14.8,30.5)	0.394	0.039
Creatinine (per μmol/L increase)	74(59,88)	66(53.9,81)	<0.001	70(57,82)	66(53.9,81)	0.081	0.069
Sodium (per mmol/L increase)	139.7(137.3,142)	139.9(137,142)	0.734	139.3(136.9,141.625)	139.9(137,142)	0.139	0.133

PT prolong (per second increase)	1.5(0,3.3)	1.9(0.4,3.3)	0.14	1.7(0.1,3.8)	1.9(0.4,3.3)	0.847	0.083
International normalized ratio (per unit increase)	1.3(1.18,1.46)	1.35(1.23,1.5)	0.017	1.31(1.19,1.4825)	1.35(1.23,1.5)	0.202	0.05
Child-Pugh score (per point increase)	7(6,8)	7(6,8)	0.034	7(6,8)	7(6,8)	0.85	0.008
Child-Pugh class (n, %)			0.197			0.94	0.026
class A	739(39.5)	79(34.8)		294(34.0)	79(34.8)		
class B	954(50.9)	119(52.4)		464(53.7)	119(52.4)		
class C	180(9.6)	29(12.8)		106(12.3)	29(12.8)		
MELD score (per point increase)	10(9,13)	11(9,13)	0.524	11(9,13)	11(9,13)	0.942	0.021
MELD-Na score (per point increase)	11(9,13)	11(9,14)	0.721	11(9,14)	11(9,14)	0.963	0.044
Ascites before TIPS (n, %)	1245(66.5)	168(74.0)	0.022	589(68.2)	168(74.0)	0.089	0.129
HE before TIPS (n, %)	19(1.0)	3(1.3)	0.668	11(1.3)	3(1.3)	0.954	0.004
Red-color sign (n, %)	1212(78.1)	121(82.9)	0.183	535(61.9)	121(53.3)		0.118

Diameter (mm)	8(8,8)	8(8,8)	0.001	8(8,8)	8(8,8)	0.274	0.066
Stent diameter groups			0.001			0.382	0.099
6-7mm	74(4.0)	21(9.3)		57(6.6)	21(9.3)		
8mm	1705(91.0)	199(87.7)		781(90.4)	199(87.7)		
10mm	94(5.0)	7(3.1)		26(3.0)	7(3.1)		

PSM, propensity score match; PPG, portal pressure gradient; TIPS, transjugular intrahepatic portosystemic shunt.

1:4 PSM with age, sex, stent diameter, Child-Pugh score, creatinine before TIPS. Caliper value was 0.4.

Supplementary Table 6 PSM for 8mmHg in Child-Pugh class A patients.

Characteristics	Before PSM			After PSM			
	<8mmHg(n=335)	≥8mmHg(n=483)	P value	<8mmHg(n=327)	≥8mmHg(n=327)	P value	SMD
Age (per year increase)	53(45,61)	49(41.5,58)	0.002	52(43,61)	53(44,61)	0.568	0.04
Sex (n, male/female)	344/139	197/138	<0.001	208/119	196/131	0.334	0.076
Etiology of cirrhosis (n, %)			0.345			0.868	0.013
Virus	338(70.0)	224(66.9)		218(66.7)	220(67.3)		
Others	145(30.0)	111(33.1)		109(33.3)	107(32.7)		
Platelet (per 10^9/L increase)	70(51,114)	60(42,88.5)	<0.001	65(44,98)	70(50,114)	0.012	0.131
Hemoglobin	88(77,102)	85(73,100)	0.058	86(74,100.25)	87(77,102)	0.165	0.111
Albumin (per g/L increase)	37.5(35.3,40.6)	37.2(35.3,40.55)	0.719	37.1(34.9,40.33)	37.5(35.4,40.7)	0.236	0.054
Total bilirubin (per g/L increase)	16.6(12.7,23.0)	18.4(13.0,24.55)	0.106	18.05(12.78,24.25)	16.8(12.7,23.1)	0.431	0.071
Creatinine (per μmol/L increase)	77(63,91)	72(58,86)	0.006	76(59.42,89)	76(63,91)	0.412	0.044

Sodium (per mmol/L increase)	140.8(138.4,142.5)	140.9(138.8,142.4)	0.811	140.7(138.6,142.73)	140.6(138.4,142.5)	0.603	0.02
PT prolong (per second increase)	0.8(0,2.1)	0.9(0,2.2)	0.238	0.9(0,2.13)	0.7(0,2.1)	0.376	0.031
International normalized ratio (per unit increase)	1.23(1.13,1.36)	1.25(1.17,1.36)	0.069	1.25(1.17,1.35)	1.23(1.13,1.35)	0.122	0.045
MELD score (per point increase)	10(8,11)	10(8,11)	0.233	10(8,11)	10(8,11)	0.327	0.07
MELD-Na score (per point increase)	10(8,11)	10(8,11)	0.989	10(8,11)	10(8,11)	0.938	0.03
Ascites before TIPS (n, %)	172(35.6)	88(26.3)	0.005	90(27.5)	88(26.9)	0.861	0.014
HE before TIPS (n, %)	1(0.2)	0(0)	0.405	0(0)	0(0)	-	<0.001
Red-color sign (n, %)	305(79.4)	205(70.7)	0.009	210(64.2)	201(61.5)	0.034	0.182
Diameter (mm)	8(8,8)	8(8,8)	0.017	8(8,8)	8(8,8)	0.544	0.053
Stent diameter groups			0.032			0.808	0.051
6-7mm	16(3.3)	3(0.9)		3(0.9)	3(0.9)		

8mm	445(92.1)	309(92.2)	306(93.6)	302(92.4)
10mm	22(4.6)	23(6.9)	18(5.5)	22(6.7)

PSM, propensity score match; PT, prothrombin time; PPG, portal pressure gradient; TIPS, transjugular intrahepatic portosystemic shunt.
1:1 PSM with age, sex, stent diameter, ascites, platelet before TIPS, Caliper value was 0.4.

Supplementary Table 7 PSM for 10mmHg in Child-Pugh class A patients.

Characteristics	Before PSM			After PSM			
	<10mmHg(n=539)	≥10mmHg(n=279)	P value	<10mmHg(n=275)	≥10mmHg(n=275)	P value	SMD
Age (per year increase)	52(44,60)	49(41,60)	0.034	50(43,58)	49(41,60)	0.703	0.02
Sex (n, male/female)	335/204	206/73	0.001	213/62	202/73	0.276	0.093
Etiology of cirrhosis (n, %)			0.245			0.567	0.049
Virus	363(67.3)	199(71.3)		202(73.5)	196(71.3)		
Others	176(32.7)	80(28.7)		73(26.5)	79(28.7)		
Platelet (per 10^9/L increase)	65(47,99)	60(42,91)	0.013	65(47.5,101.5)	60(42,91)	0.031	0.144
Hemoglobin	87(76,102)	84(72,98)	0.021	87(75,104)	84(72,98)	0.06	0.166
Albumin (per g/L increase)	37.5(35.3,40.6)	37.2(35.3,40.4)	0.556	37.5(35.3,40.5)	37.2(35.3,40.4)	0.837	0.008
Total bilirubin (per g/L increase)	16.9(12.7,23.3)	18.5(13.5,24.7)	0.096	18.1(12.8,24.7)	18.5(13.5,24.7)	0.738	0.026
Creatinine (per μmol/L increase)	76(62,90)	71(57,85)	0.002	74(60.5,89)	71(58,85)	0.085	0.093

Sodium (per mmol/L increase)	140.9(138.55,142.45)	140.7(138.6,142.6)	0.677	140.7(138.5,142.3)	140.6(138.6,142.7)	0.917	0.018
PT prolong (per second increase)	0.8(0,2.1)	1(0,2.3)	0.222	0.9(0,2.15)	1(0,2.2)	0.431	0.026
International normalized ratio (per unit increase)	1.24(1.15,1.35)	1.25(1.17,1.37)	0.111	1.26(1.16,1.365)	1.25(1.17,1.37)	0.885	0.09
MELD score (per point increase)	10(8,11)	10(9,11)	0.114	10(8,11)	10(9,11)	0.658	0.045
MELD-Na score (per point increase)	10(8,11)	10(9,11)	0.237	10(8,11)	10(9,11)	0.615	0.052
Ascites before TIPS (n, %)	162(30.1)	98(35.1)	0.14	71(25.8)	96(34.9)	0.02	0.199
HE before TIPS (n, %)	1(0.2)	0(0)	0.472	1(0.4)	0(0)	0.317	0.085
Red-color sign (n, %)	335(73.0)	175(81.4)	0.018	171(73.1)	174(81.7)	0.03	0.207
Diameter (mm)	8(8,8)	8(8,8)	0.009	8(8,8)	8(8,8)	0.86	0.03
Stent diameter groups			0.022			0.67	0.076
6-7mm	8(1.5)	11(3.9)		8(2.9)	10(3.6)		

8mm	496(92.0)	258(92.5)	260(94.5)	255(92.7)
10mm	35(6.5)	10(3.6)	7(2.5)	10(3.6)

PSM, propensity score match; PT, prothrombin time; PPG, portal pressure gradient; TIPS, transjugular intrahepatic portosystemic shunt.
1:1 PSM with age, sex, MELD score, stent diameter, creatinine before TIPS, Caliper value was 0.4.

Supplementary Table 8 PSM for 12mmHg in Child-Pugh class A patients.

Characteristics	Before PSM				After PSM			
	<12mmHg(n=677)	≥12mmHg(n=141)	P value	SMD	<12mmHg(n=380)	≥12mmHg(n=141)	P value	SMD
Age (per year increase)	51(43,60)	50(42,60.5)	0.935	0.014	51(43,60)	50(42,60.5)	0.916	0.004
Sex (n, male/female)	438/239	103/38	0.057	0.181	271/109	103/38	0.696	0.039
Etiology of cirrhosis (n, %)			0.566	0.053			0.703	0.037
Virus	468(69.1)	47(33.3)			260(68.4)	94(66.7)		
Others	209(31.3)	94(66.7)			120(31.6)	47(33.3)		
Platelet (per 10^9/L increase)	65(46,98)	56(41.5,87)	0.013	0.146	63(45,92)	56(41.5,87)	0.133	0.055
Hemoglobin	87(76,101)	82(72,101.5)	0.179	0.073	87(75,101)	82(72,101.5)	0.312	0.059
Albumin (per g/L increase)	37.5(35.3,40.6)	37.2(35.15,40.4)	0.47	0.076	37.4(35.3,40.5)	37.2(35.15,40.4)	0.662	0.04
Total bilirubin (per g/L increase)	17.5(12.7,23.7)	18(13.5,24.5)	0.445	0.073	18.2(13.3,24.7)	18(13.5,24.5)	0.96	0.007
Creatinine (per μmol/L	76(61.4,90)	63(52.8,80)	<0.001	0.341	69(57,81)	63(52.8,80)	0.106	0.097

increase)								
Sodium (per mmol/L increase)	140.7(138.6,142.4)	141(138.45,143)	0.665	0.052	140.6(138.3,142)	141(138.45,143)	0.183	0.138
PT prolong (per second increase)	0.89(0,2.1)	0.9(0,2.2)	0.648	0.014	0.8(0,2.1)	0.9(0,2.2)	0.642	0.017
International normalized ratio (per unit increase)	1.25(1.15,1.35)	1.25(1.165,1.375)	0.471	0.116	1.24(1.14,1.35)	1.25(1.165,1.375)	0.229	0.152
MELD score (per point increase)	10(8,11)	10(8,11)	0.809	0.086	10(8,11)	10(8,11)	0.28	0.169
MELD-Na score (per point increase)	10(8,11)	10(8,11)	0.88	0.053	10(8,11)	10(8,11)	0.538	0.084
Ascites before TIPS (n, %)	209(30.9)	51(36.2)	0.219	0.112	104(27.4)	51(36.2)	0.051	0.19
HE before TIPS (n, %)	1(0.1)	0(0)	0.648	0.054	1(0.3)	0(0)	0.542	0.073
Red-color sign (n, %)	427(74.3)	83(83.8)	0.04	0.237	220(71.9)	83(83.8)	0.017	0.291
Diameter (mm)	8(8,8)	8(8,8)	0.134	0.146	8(8,8)	8(8,8)	0.221	0.125
Stent diameter groups			0.029	0.16			0.453	0.131

6-7mm	15(2.2)	4(2.8)	8(2.1)	4(2.8)
8mm	621(91.7)	133(94.3)	352(92.6)	133(94.3)
10mm	41(6.1)	4(2.8)	20(5.3)	4(2.8)

PSM, propensity score match; PT, prothrombin time; PPG, portal pressure gradient; TIPS, transjugular intrahepatic portosystemic shunt.
1:2 PSM with sex, creatinine, platelet before TIPS, Caliper value was 0.4.

Supplementary Table 9 PSM for 14mmHg in Child-Pugh class A patients.

Characteristics	Before PSM			After PSM			
	<14mmHg(n=739)	≥14mmHg(n=79)	P value	<14mmHg(n=547)	≥14mmHg(n=79)	P value	SMD
Age (per year increase)	51(43,60)	50(41,61)	0.9	51(43,60)	50(41,61)	0.905	0.03
Sex (n, male/female)	485/254	56/23	0.348	339/208	56/23	0.125	0.19
Etiology of cirrhosis (n, %)			0.403			0.751	0.038
Virus	511(69.1)	51(64.6)		363(66.4)	51(64.6)		
Others	228(30.9)	28(35.4)		184(33.6)	28(35.4)		
Platelet (per 10^9/L increase)	65(45,98)	53(42,87)	0.063	65(45,98)	53(42,87)	0.069	0.122
Hemoglobin	87(75,101)	82(72,104)	0.463	86(75,100)	82(72,104)	0.648	0.007
Albumin (per g/L increase)	37.4(35.3,40.6)	37.1(34.7,39.6)	0.104	37.4(35.3,40.5)	37.1(34.7,39.6)	0.172	0.198
Total bilirubin (per g/L increase)	17.6(12.7,23.7)	17.8(13.5,24.8)	0.682	18.1(12.9,23.7)	17.8(13.5,24.8)	0.859	0.034
Creatinine (per μmol/L increase)	75(60.5,89)	63(52.6,80)	<0.001	68.1(57,81)	63(52.6,80)	0.22	0.043

Sodium (per mmol/L increase)	140.8(138.6,142.5)	141(138.5,142.8)	0.903	140.9(138.6,142.5)	141(138.5,142.8)	0.912	0.112
PT prolong (per second increase)	0.8(0,2.1)	1.3(0.2,2.4)	0.06	0.8(0,2.1)	1.3(0.2,2.4)	0.041	0.175
International normalized ratio (per unit increase)	1.25(1.155,1.36)	1.24(1.16,1.37)	0.594	1.24(1.15,1.36)	1.24(1.16,1.37)	0.413	0.018
MELD score (per point increase)	10(8,11)	10(9,12)	0.413	10(8,11)	10(9,12)	0.094	0.213
MELD-Na score (per point increase)	10(8,11)	10(9,12)	0.629	10(8,11)	10(9,12)	0.206	0.055
Ascites before TIPS (n, %)	229(31.0)	31(39.2)	0.134	168(30.7)	31(39.2)	0.128	0.18
HE before TIPS (n, %)	1(0.1)	0(0)	0.744	1(0.2)	0(0)	0.704	0.061
Red-color sign (n, %)	470(75.1)	40(83.3)	0.199	338(76.1)	40(83.3)	0.261	0.18
Diameter (mm)	8(8,8)	8(8,8)	0.136	8(8,8)	8(8,8)	0.285	0.121
Stent diameter groups			0.326			0.56	0.136
6-7mm	16(2.2)	3(3.8)		14(2.6)	3(3.8)		

8mm	680(92.0)	74(93.7)	507(92.7)	74(93.7)
10mm	43(5.8)	2(2.5)	26(4.8)	2(2.5)

PSM, propensity score match; PT, prothrombin time; PPG, portal pressure gradient; TIPS, transjugular intrahepatic portosystemic shunt.
1:7 PSM with age, creatinine before TIPS, Caliper value was 0.4.

Supplementary Table 10 PSM for 8mmHg in Child-Pugh class B patients.

Characteristics	Before PSM			After PSM			
	<8mmHg(n=410)	≥8mmHg(n=663)	P value	<8mmHg(n=410)	≥8mmHg(n=410)	P value	SMD
Age (per year increase)	55(47,63)	51(44,59)	<0.001	55(47,63)	55(47.25,62)	0.803	0.019
Sex (n, male/female)	214/196	449/214	<0.001	214/196	227/183	0.363	0.064
Etiology of cirrhosis (n, %)			0.188			0.827	0.015
Virus	261(63.7)	448(67.6)		261(63.7)	264(64.4)		
Others	149(36.3)	215(32.4)		149(36.3)	146(35.6)		
Platelet (per 10^9/L increase)	60(41,96.5)	56(40,81)	0.008	60(41,96.5)	56.5(41,83)	0.034	0.219
Hemoglobin	78(68,89)	79(67,92)	0.32	78(68,89)	79(69,91.75)	0.219	0.099
Albumin (per g/L increase)	31.7(29.1,34.5)	32.15(29.4,34.5)	0.315	31.7(29.1,34.5)	32.35(29.5,34.5)	0.167	0.039
Total bilirubin (per g/L increase)	21(14.15,30.45)	21.78(15.63,31.8)	0.084	21(14.15,30.45)	21.6(15.225,32.075)	0.982	0.076
Creatinine (per μmol/L increase)	74(60,88)	70.5(57,86)	0.063	74(60,88)	72.5(58,91.675)	0.75	0.036

Sodium (per mmol/L increase)	139.2(136.7,141.6)	139.2(136.3,141.8)	0.935	139.2(136.7,141.6)	139.25(136.65,141.8)	0.073	0.008
PT prolong (per second increase)	1.5(0,3.4)	1.9(0.4,3.6)	0.016	1.5(0,3.4)	1.9(0.325,3.4)	0.276	0.128
International normalized ratio (per unit increase)	1.31(1.18,1.48)	1.33(1.22,1.48)	0.051	1.31(1.18,1.48)	1.32(1.22,1.46)	0.447	0.034
MELD score (per point increase)	11(9,13)	11(9,13)	0.418	11(9,13)	11(9,13)	0.447	0.047
MELD-Na score (per point increase)	11(9,14)	11(9,14)	0.381	11(9,14)	11(9,13.75)	0.747	0.005
Ascites before TIPS (n, %)	365(89)	581(87.6)	0.493	365(89)	356(86.8)	0.335	0.067
HE before TIPS (n, %)	6(1.5)	9(1.4)	0.886	6(1.5)	4(1)	0.525	0.044
Red-color sign (n, %)	285(81.4)	408(79.7)	0.527	285(81.4)	260(80.5)	0.758	0.024
Diameter (mm)	8(8,8)	8(8,8)	0.003	8(8,8)	8(8,8)	0.922	0.013
Stent diameter groups			0.005			0.983	0.013
6-7mm	15(3.7)	57(8.6)		15(3.7)	16(3.9)		

8mm	376(91.7)	583(87.9)	376(91.7)	375(91.5)	
10mm	19(4.6)	23(3.5)	19(4.6)	19(4.6)	

PSM, propensity score match; PT, prothrombin time; PPG, portal pressure gradient; TIPS, transjugular intrahepatic portosystemic shunt.
1:1 PSM with age, sex, creatinine, stent diameter before TIPS, Caliper value was 0.4.

Supplementary Table 11 PSM for 10mmHg in Child-Pugh class B patients.

Characteristics	Before PSM			After PSM			
	<10mmHg(n=644)	≥10mmHg(n=429)	P value	<10mmHg(n=426)	≥10mmHg(n=426)	P value	SMD
Age (per year increase)	54(47,62)	51(43,58)	<0.001	50(44,59)	51(43.25,58)	0.633	0.03
Sex (n, male/female)	362/282	301/128	<0.001	301/125	299/127	0.881	0.01
Etiology of cirrhosis (n, %)			0.84			0.088	0.117
Virus	424(65.8)	285(66.4)		306(71.8)	283(66.4)		
Others	220(34.2)	144(33.6)		120(28.2)	143(33.6)		
Platelet (per 10^9/L increase)	58.5(41,87.25)	57(40,82)	0.116	60(40,88)	57(40,81.75)	0.148	0.109
Hemoglobin	79(68,91)	78(67,93)	0.765	78(67,89)	78(67,92)	0.521	0.068
Albumin (per g/L increase)	31.8(29.2,34.4)	32.2(29.3,34.7)	0.294	31.75(29.225,34.4)	32.2(29.3,34.675)	0.298	0.068
Total bilirubin (per g/L increase)	21.4(14.5,30.5)	22.2(15.5,32.7)	0.061	21.4(14.5,30.05)	22.195(15.525,32.7)	0.073	0.158
Creatinine (per μmol/L increase)	73(59,87)	69(56,86)	0.032	73(59,87)	69(56.125,86)	0.052	0.103

Sodium (per mmol/L increase)	139.2(136.78,141.53)	139.4(136,141.9)	0.894	139.1(136.8,141.5)	139.45(136,141.9)	0.658	0.025
PT prolong (per second increase)	1.6(0.1,3.425)	2(0.2,3.7)	0.068	1.7(0.2,3.3)	2(0.2,3.7)	0.137	0.086
International normalized ratio (per unit increase)	1.32(1.20,1.47)	1.34(1.23,1.48)	0.118	1.32(1.2,1.48)	1.34(1.23,1.48)	0.261	0.068
MELD score (per point increase)	11(9,13)	11(9,13)	0.155	11(9,13)	11(9,13)	0.143	0.054
MELD-Na score (per point increase)	11(9,13)	12(9,14)	0.112	11(9,13)	12(9,14)	0.065	0.092
Ascites before TIPS (n, %)	570(88.5)	376(87.6)	0.668	383(89.9)	374(87.8)	0.327	0.067
HE before TIPS (n, %)	7(1.1)	8(1.9)	0.288	5(1.2)	8(1.9)	0.402	0.057
Red-color sign (n, %)	436(81)	257(79.3)	0.538	284(81.1)	256(79.5)	0.593	0.041
Diameter (mm)	8(8,8)	8(8,8)	0.035	8(8,8)	8(8,8)	0.38	0.044
Stent diameter groups			0.039			0.595	0.07
6-7mm	33(9.1)	39(9.1)		30(7)	38(8.9)		

8mm	585(90.8)	374(87.2)	379(89)	372(87.3)
10mm	26(4)	16(3.7)	17(4)	16(3.8)

PSM, propensity score match; PT, prothrombin time; PPG, portal pressure gradient; TIPS, transjugular intrahepatic portosystemic shunt.
1:1 PSM with age, sex, creatinine, stent diameter before TIPS, Caliper value was 0.4.

Supplementary Table 12 PSM for 12mmHg in Child-Pugh class B patients.

Characteristics	Before PSM			After PSM			
	<12mmHg(n=846)	≥12mmHg(n=227)	P value	<12mmHg(n=438)	≥12mmHg(n=219)	P value	SMD
Age (per year increase)	53(46,61)	51(43,57)	0.001	50(44,59)	51(44,58)	0.843	0.017
Sex (n, male/female)	494/352	169/58	<0.001	327/111	161/58	0.825	0.026
Etiology of cirrhosis (n, %)			0.058			0.009	0.213
Virus	571(67.5)	138(60.8)		312(71.2)	134(61.2)		
Others	275(32.5)	89(39.2)		126(28.8)	85(38.8)		
Platelet (per 10^9/L increase)	58(40,86)	58(41,85)	0.915	56.5(39.75,87)	58(41,85)	0.796	0.022
Hemoglobin	79(68,91)	76(65,90)	0.087	79(68,91)	77(65,92)	0.311	0.083
Albumin (per g/L increase)	31.9(29.2,34.5)	32.3(29.5,34.6)	0.622	31.8(29.2,34.3)	32.3(29.6,34.6)	0.383	0.073
Total bilirubin (per g/L increase)	21.65(14.9,31.425)	21.5(15,31.2)	0.871	22.15(15.4,31.85)	21.6(15,31.1)	0.825	0.018
Creatinine (per μmol/L increase)	73(59,88)	67(55,81)	<0.001	69.5(57,83)	67(55,80)	0.198	0.107

Sodium (per mmol/L increase)	139.2(136.5,141.7)	139.4(136,142)	0.717	139.15(136.9,141.225)	139.4(136,141.9)	0.778	0.023
PT prolong (per second increase)	1.7(0.1,3.6)	1.9(0.4,3.4)	0.428	1.7(0.2,3.7)	1.9(0.2,3.3)	0.447	0.064
International normalized ratio (per unit increase)	1.325(1.2,1.48)	1.34(1.23,1.47)	0.526	1.32(1.22,1.5025)	1.33(1.22,1.47)	0.205	0.109
MELD score (per point increase)	11(9,13)	11(9,13)	0.066	11(9,13)	10(9,13)	0.013	0.203
MELD-Na score (per point increase)	11(9,14)	11(9,14)	0.208	11(9,14)	11(9,14)	0.26	0.093
Ascites before TIPS (n, %)	742(87.7)	204(89.9)	0.371	382(87.2)	200(91.3)	0.152	0.133
HE before TIPS (n, %)	9(1.1)	6(2.6)	0.072	5(1.1)	5(2.3)	0.43	0.088
Red-color sign (n, %)	568(81.0)	125(77.6)	0.329	270(76.9)	118(77.1)	1	0.005
Diameter (mm)	8(8,8)	8(8,8)	0.004	8(8,8)	8(8,8)	0.528	0.051
Stent diameter groups			0.005			0.19	0.147
6-7mm	46(5.4)	26(11.5)		35(8.0)	26(11.9)		

8mm	765(90.4)	194(85.5)	395(90.2)	187(85.4)
10mm	35(4.1)	7(3.1)	8(1.8)	6(2.7)

PSM, propensity score match; PT, prothrombin time; PPG, portal pressure gradient; TIPS, transjugular intrahepatic portosystemic shunt.
1:2 PSM with age, sex, creatinine, stent diameter before TIPS, Caliper value was 0.4.

Supplementary Table 13 PSM for 14mmHg in Child-Pugh class B patients.

Characteristics	Before PSM			After PSM			
	<14mmHg(n=954)	≥14mmHg(n=119)	P value	<14mmHg(n=622)	≥14mmHg(n=119)	P value	SMD
Age (per year increase)	53(46,61)	50(42,57)	0.002	51(44,59)	50(42,57)	0.236	0.112
Sex (n, male/female)	573/381	90/29	0.001	450/172	90/29	0.461	0.075
Etiology of cirrhosis (n, %)			0.076			0.033	0.208
Virus	639(67.0)	70(58.8)		428(68.8)	70(58.8)		
Others	315(33.0)	49(41.2)		194(31.2)	49(41.2)		
Platelet (per 10^9/L increase)	58(41,87)	56(41,76)	0.261	57(40,88)	56(41,76)	0.398	0.145
Hemoglobin	79(68,92)	74(64,87)	0.025	78(67,89)	74(64,87)	0.189	0.169
Albumin (per g/L increase)	31.8(29.2,34.5)	32.5(29.5,34.6)	0.558	31.7(29.1,34.4)	32.5(29.5,34.6)	0.358	0.05
Total bilirubin (per g/L increase)	21.6(14.9,31.5)	21.5(15.4,30.5)	0.954	21.6(14.9,31.05)	21.5(15.4,30.5)	0.807	0.063
Creatinine (per μmol/L increase)	73(58,87)	66(53.9,80)	0.002	69(57,84.1)	66(53.9,80)	0.096	0.134

Sodium (per mmol/L increase)	139.2(136.4,141.6)	140(136.5,142)	0.293	139.1(136.4,141.525)	140(136.5,142)	0.215	0.119
PT prolong (per second increase)	1.8(0.1,3.625)	1.9(0.4,3.1)	0.866	1.8(0.1,3.8)	1.9(0.4,3.1)	0.674	0.112
International normalized ratio (per unit increase)	1.32(1.2,1.48)	1.37(1.26,1.47)	0.165	1.33(1.21,1.49)	1.37(1.26,1.47)	0.343	0.007
MELD score (per point increase)	11(9,13)	11(9,13)	0.45	11(9,13)	11(9,13)	0.777	0.055
MELD-Na score (per point increase)	11(9,14)	11(9,13)	0.456	11(9,14)	11(9,13)	0.687	0.056
Ascites before TIPS (n, %)	838(87.8)	108(90.8)	0.353	548(88.1)	108(90.8)	0.405	0.086
HE before TIPS (n, %)	13(1.4)	2(1.7)	0.781	10(1.6)	2(1.7)	0.954	0.006
Red-color sign (n, %)	627(80.2)	66(82.5)	0.618	383(77.2)	66(82.5)	0.29	0.132
Diameter (mm)	8(8,8)	8(8,8)	0.009	8(8,8)	8(8,8)	0.152	0.117
Stent diameter groups			0.008			0.113	0.194
6-7mm	56(5.9)	16(13.4)		49(7.9)	16(13.4)		

8mm	860(90.1)	99(83.2)	558(89.7)	99(83.2)
10mm	38(4.0)	4(3.4)	15(2.4)	4(3.4)

PSM, propensity score match; PT, prothrombin time; PPG, portal pressure gradient; TIPS, transjugular intrahepatic portosystemic shunt.
1:6 PSM with age, sex, creatinine, stent diameter before TIPS, Caliper value was 0.4.

Supplementary Table 14 PSM for 8mmHg in Child-Pugh class C patients.

Characteristics	Before PSM			After PSM			
	<8mmHg(n=76)	≥8mmHg(n=133)	P value	<8mmHg(n=72)	≥8mmHg(n=72)	P value	SMD
Age (per year increase)	53.5(47.25,62.75)	49(41,57)	0.001	53(45.5,61)	53(45.25,60.75)	0.659	0.057
Sex (n, male/female)	50/26	97/36	0.277	48/24	51/21	0.59	0.09
Etiology of cirrhosis (n, %)			0.757			0.379	0.146
Virus	51(67.1)	92(69.2)		50(69.4)	45(62.5)		
Others	25(32.9)	41(30.8)		22(30.6)	27(37.5)		
Platelet (per 10^9/L increase)	43(31,71.75)	48(32.5,69.5)	0.76	43.5(32,71.75)	48(31,73)	0.868	0.015
Hemoglobin	75.5(64,90.5)	76(68,87)	0.813	75.5(63.25,91)	78.5(69,86.75)	0.38	0.138
Albumin (per g/L increase)	28.1(25.93,31.48)	28.1(24.8,31.75)	0.601	28.2(25.75,31.475)	28.1(25.05,31.775)	0.725	0.033
Total bilirubin (per g/L increase)	37.65(30.28,57.7)	37.1(26.2,52.2)	0.468	38.35(30.05,57.7)	39.4(26.85,54.5375)	0.727	0.022
Creatinine (per μmol/L increase)	77(65,97.75)	71.06(58.5,85)	0.035	76(62,92)	75.5(63,91.75)	0.992	0.005

Sodium (per mmol/L increase)	138.3(135.13,140.98)	137.6(134.6,139.7)	0.202	138.3(135.125,140.975)	137.4(134.425,139.375)	0.088	0.276
PT prolong (per second increase)	6.5(3.5,8.88)	6.2(4.4,7.65)	0.776	6.5(3.4,8.45)	6(4.8,7.5)	0.826	0.093
International normalized ratio (per unit increase)	1.64(1.4,1.97)	1.62(1.44,1.80)	0.384	1.655(1.3925,1.9725)	1.645(1.4275,1.7775)	0.338	0.157
MELD score (per point increase)	15(13,18)	15(12,17)	0.156	15(13,17.75)	15(12,17)	0.489	0.008
MELD-Na score (per point increase)	16(13.25,19)	16(13,17)	0.274	16(13,19)	16(13,18)	0.869	0.083
Ascites before TIPS (n, %)	74(97.4)	133(100)	0.06	70(97.2)	72(100)	0.154	0.239
HE before TIPS (n, %)	2(2.6)	4(3.0)	0.876	2(2.8)	2(2.8)	1	<0.001
Red-color sign (n, %)	47(77)	83(83)	0.352	44(77.2)	44(80)	0.717	0.068
Diameter (mm)	8(8,8)	8(8,8)	0.517	8(8,8)	8(8,8)	0.616	0.048
Stent diameter groups			0.31			0.2	0.302
6-7mm	0(0)	4(3)		0(0)	3(4.2)		

8mm	71(93.4)	120(90.2)	67(93.1)	63(87.5)
10mm	5(6.6)	9(6.8)	5(6.9)	6(8.3)

PSM, propensity score match; PT, prothrombin time; PPG, portal pressure gradient; TIPS, transjugular intrahepatic portosystemic shunt.
1:1 PSM with age, creatinine before TIPS, Caliper value was 0.4.

Supplementary Table 15 PSM for 10mmHg in Child-Pugh class C patients.

Characteristics	Before PSM			After PSM			
	<10mmHg(n=120)	≥10mmHg(n=89)	P value	<10mmHg(n=85)	≥10mmHg(n=85)	P value	SMD
Age (per year increase)	52(44,61)	49(41.5,57)	0.091	49(43,55.5)	49(41.5,56.5)	0.916	0.111
Sex (n, male/female)	78/42	69/20	0.05	58/27	67/18	0.118	0.259
Etiology of cirrhosis (n, %)			0.975			0.867	<0.001
Virus	82(68.3)	61(68.5)		60(70.6)	59(69.4)		
Others	38(31.7)	28(31.5)		25(29.4)	26(30.6)		
Platelet (per 10^9/L increase)	43.5(32.25,71)	51(30,71)	0.576	43(35,72)	51(30,71)	0.836	0.01
Hemoglobin	77(65,88.75)	75(67,86.5)	0.67	75(64,89)	76(67.5,87.5)	0.935	0.048
Albumin (per g/L increase)	28.2(24.93,31.88)	28.1(25.2,31.15)	0.564	27.9(24.8,31.85)	28.1(24.9,31.05)	0.754	0.109
Total bilirubin (per g/L increase)	37.15(29.88,53.7)	39(25.35,53.05)	0.5	37.2(28.4,53.7)	39.8(25.35,53.05)	0.692	0.006
Creatinine (per μmol/L increase)	74.5(61,92)	72(58.5,86)	0.27	72(59,86.5)	73(59,86)	0.963	0.041

Sodium (per mmol/L increase)	138.2(134.83,140.48)	137.4(134.8,139.7)	0.282	138.2(134.65,140.45)	137.4(134.8,139.5)	0.303	0.088
PT prolong (per second increase)	6.5(4.43,8.88)	6(4.15,7.45)	0.103	6.2(4.35,8.4)	6.2(4.25,7.55)	0.479	0.133
International normalized ratio (per unit increase)	1.65(1.43,2.00)	1.61(1.41,1.78)	0..054	1.66(1.4,1.93)	1.61(1.42,1.78)	0.213	0.123
MELD score (per point increase)	16(13,18)	14(11.5,16)	0.008	15(13,17)	14(12,16)	0.138	0.116
MELD-Na score (per point increase)	16(14,19)	15(12,17)	0.019	16(13,19)	15(12,17)	0.171	0.132
Ascites before TIPS (n, %)	118(98.3)	89(100)	0.221	83(97.6)	85(100)	0.155	0.152
HE before TIPS (n, %)	3(2.5)	3(3.4)	0.709	2(2.4)	2(2.4)	1	<0.001
Red-color sign (n, %)	78(80.4)	52(81.3)	0.895	52(78.8)	51(81)	0.759	0.059
Diameter (mm)	8(8,8)	8(8,8)	0.043	8(8,8)	8(8,8)	0.407	0.06
Stent diameter groups			0.038			0.406	0.254
6-7mm	0(0)	4(4.5)		0(0)	0(0)		

8mm	110(91.7)	81(91)	83(97.6)	81(95.3)
10mm	10(8.3)	4(4.5)	2(2.4)	4(4.7)

PSM, propensity score match; PT, prothrombin time; PPG, portal pressure gradient; TIPS, transjugular intrahepatic portosystemic shunt.
1:1 PSM with age, MELD score, stent diameter before TIPS, Caliper value was 0.4.

Supplementary Table 16 PSM for 12mmHg in Child-Pugh class C patients.

Characteristics	Before PSM			After PSM			
	<12mmHg(n=159)	≥12mmHg(n=50)	P value	<12mmHg(n=125)	≥12mmHg(n=48)	P value	SMD
Age (per year increase)	51(43,60)	48.5(42.75,57)	0.304	49(41.5,57.5)	48(42.25,55)	0.62	0.127
Sex (n, male/female)	106/53	41/9	0.038	104/21	41/7	0.723	0.061
Etiology of cirrhosis (n, %)			0.441			0.46	0.123
Virus	111(69.8)	32(64)		88(70.4)	31(64.6)		
Others	48(30.2)	18(36)		37(29.6)	17(35.4)		
Platelet (per 10^9/L increase)	48(33,70)	45(29.75,73.5)	0.873	48(31.5,69)	44.5(29.25,72.75)	0.864	0.057
Hemoglobin	78(67,88)	72(66,86.25)	0.161	76(65,86.5)	73(66.25,86.75)	0.387	0.15
Albumin (per g/L increase)	28.1(24.8,31.8)	28.2(26.225,31.225)	0.724	27.9(24.8,31.65)	28.2(26.3,31.175)	0.666	0.052
Total bilirubin (per g/L increase)	36.7(28.2,53.3)	40.75(25.525,55.0375)	0.884	36.2(27.75,52.75)	41.45(26.65,55.4125)	0.526	0.116
Creatinine (per μmol/L increase)	74(61,89)	72(57.75,86)	0.341	76(63.5,92)	72.26(58.25,86)	0.282	0.151

Sodium (per mmol/L increase)	138(134.8,140.3)	137.25(134.875,139.8)	0.521	137.9(134.5,140.2)	137.25(135.05,139.75)	0.7	0.001
PT prolong (per second increase)	6.5(4.3,8.5)	5.55(4.1,7.425)	0.137	6.5(4.35,8.6)	5.55(4.2,7.475)	0.182	0.224
International normalized ratio (per unit increase)	1.62(1.4,1.9)	1.66(1.5075,1.8725)	0.766	1.62(1.42,1.89)	1.66(1.51,1.8775)	0.872	0.13
MELD score (per point increase)	15(13,17)	15(12,16.25)	0.636	15(12.5,17)	15(12.25,16.75)	0.92	0.17
MELD-Na score (per point increase)	16(13,19)	16(13,17)	0.461	16(13,18.5)	16(13,17)	0.453	0.203
Ascites before TIPS (n, %)	157(98.7)	50(100)	0.426	123(98.4)	48(100.0)	0.378	0.18
HE before TIPS (n, %)	5(3.1)	1(2.0)	0.672	5(4.0)	0(0)	0.16	0.289
Red-color sign (n, %)	100(79.4)	30(85.7)	0.399	77(79.4)	30(85.7)	0.412	0.167
Diameter (mm)	8(8,8)	8(8,8)	0.064	8(8,8)	8(8,8)	0.645	0.039
Stent diameter groups			0.04			0.769	0.111
6-7mm	1(0.6)	3(6.0)		1(0.8)	1(2.1)		

8mm	146(91.8)	45(90.0)		118(94.4)	45(93.8)
10mm	12(7.5)	2(4.0)		6(4.8)	2(4.2)

PSM, propensity score match; PT, prothrombin time; PPG, portal pressure gradient; TIPS, transjugular intrahepatic portosystemic shunt.
1:3 PSM with sex, stent diameter before TIPS, Caliper value was 0.4.

Supplementary Table 17 PSM for 14mmHg in Child-Pugh class C patients.

Characteristics	Before PSM			After PSM			
	<14mmHg (n=180)	≥14mmHg (n=29)	P value	<14mmHg (n=134)	≥14mmHg (n=28)	P value	SMD
Age (per year increase)	51(43,59)	48(41.5,56)	0.224	49(42,57)	48(41.5,56)	0.512	0.211
Sex (n, male/female)	122/58	25/4	0.044	118/16	25/3	0.855	0.039
Etiology of cirrhosis (n, %)			0.717			0.596	0.109
Virus	124(68.9)	19(65.5)		93(69.4)	18(64.3)		
Others	56(31.1)	10(34.5)		41(30.6)	10(35.7)		
Platelet (per 10^9/L increase)	49(33,71)	40(28,62)	0.207	49(31,71)	40(28,62)	0.186	0.253
Hemoglobin	76.5(66.25,88)	71(66.5,88)	0.394	75.5(65,86.25)	71(66.5,88)	0.778	0.002
Albumin (per g/L increase)	28.1(24.925,31.575)	28.3(25.55,32.25)	0.511	27.8(24.8,30.725)	28.3(25.55,32.25)	0.495	0.099
Total bilirubin (per g/L increase)	36.935(28.35,53.7)	40.6(21.95,51.7)	0.662	36.7(28.45,53.15)	40.6(21.95,51.7)	0.866	0.034
Creatinine (per μmol/L increase)	74(60.25,89)	67.2(59.5,86)	0.514	75(62.75,92)	67.2(59.5,86)	0.368	0.095

Sodium (per mmol/L increase)	137.95(134.625,140.3)	137.6(135.9,139.7)	0.979	137.45(134.475,140.15)	137.6(135.9,139.7)	0.863	0.123
PT prolong (per second increase)	6.4(4.3,8.275)	5.4(4.1,7.65)	0.162	6.4(4.375,8.125)	5.4(4.1,7.65)	0.199	0.259
International normalized ratio (per unit increase)	1.62(1.42,1.88)	1.69(1.455,1.88)	0.617	1.635(1.4375,1.8725)	1.69(1.455,1.88)	0.621	0.122
MELD score (per point increase)	15(12.25,17)	16(13,17)	0.679	15(12.75,17)	16(13,17)	0.355	0.034
MELD-Na score (per point increase)	16(13,19)	16(14,17)	0.714	16(13,19)	16(14,17)	0.889	0.133
Ascites before TIPS (n, %)	178(98.9)	29(100.0)	0.568	132(98.5)	28(100)	0.515	0.174
HE before TIPS (n, %)	5(2.8)	1(3.4)	0.841	5(3.7)	0(0)	0.299	0.278
Red-color sign (n, %)	115(80.4)	15(83.3)	0.768	84(80.8)	15(83.3)	0.321	0.067
Diameter (mm)	8(8,8)	8(8,8)	0.106	8(8,8)	8(8,8)	0.655	0.038
Stent diameter groups			0.086			0.759	0.133
6-7mm	2(1.1)	2(6.9)		2(1.5)	1(3.6)		

8mm	165(91.7)	26(89.7)	127(94.8)	26(92.9)
10mm	13(7.2)	1(3.4)	5(3.7)	1(3.6)

PSM, propensity score match; PT, prothrombin time; PPG, portal pressure gradient; TIPS, transjugular intrahepatic portosystemic shunt.
1:5 PSM with sex, stent diameter before TIPS, Caliper value was 0.4.

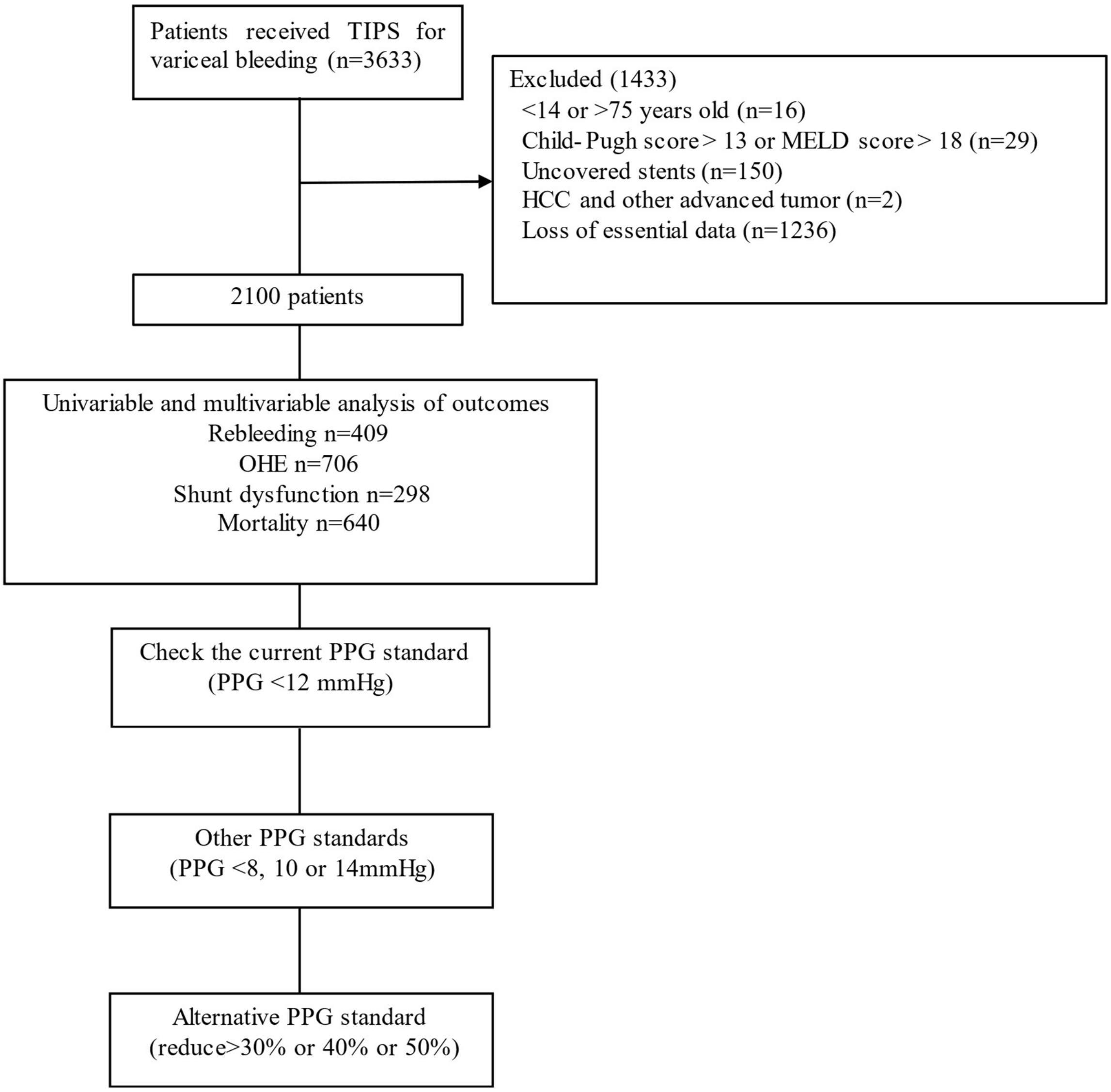

Supplementary Figure 1 Flow chart

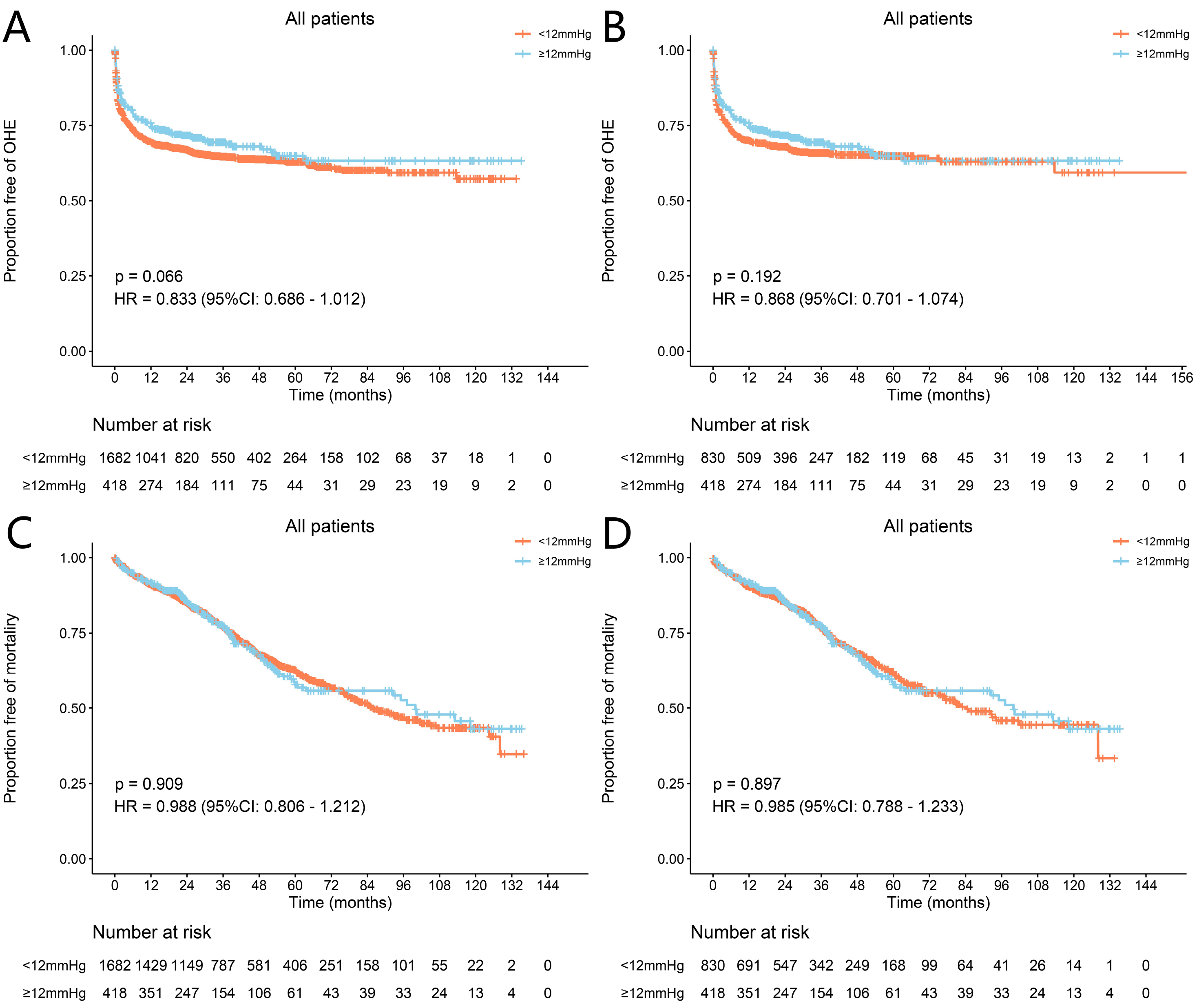

Supplementary Figure 2 Proportion free of OHE and mortality in all patients with 12mmHg post-TIPS PPG before and after PSM.

(A) Proportion free of OHE in all patients with 12mmHg threshold before PSM. (B) Proportion free of OHE in all patients with 12mmHg threshold after PSM. (C) Proportion free of mortality of all patients with 12mmHg threshold before PSM. (D) Proportion free of mortality of all patients with 12mmHg threshold after PSM.

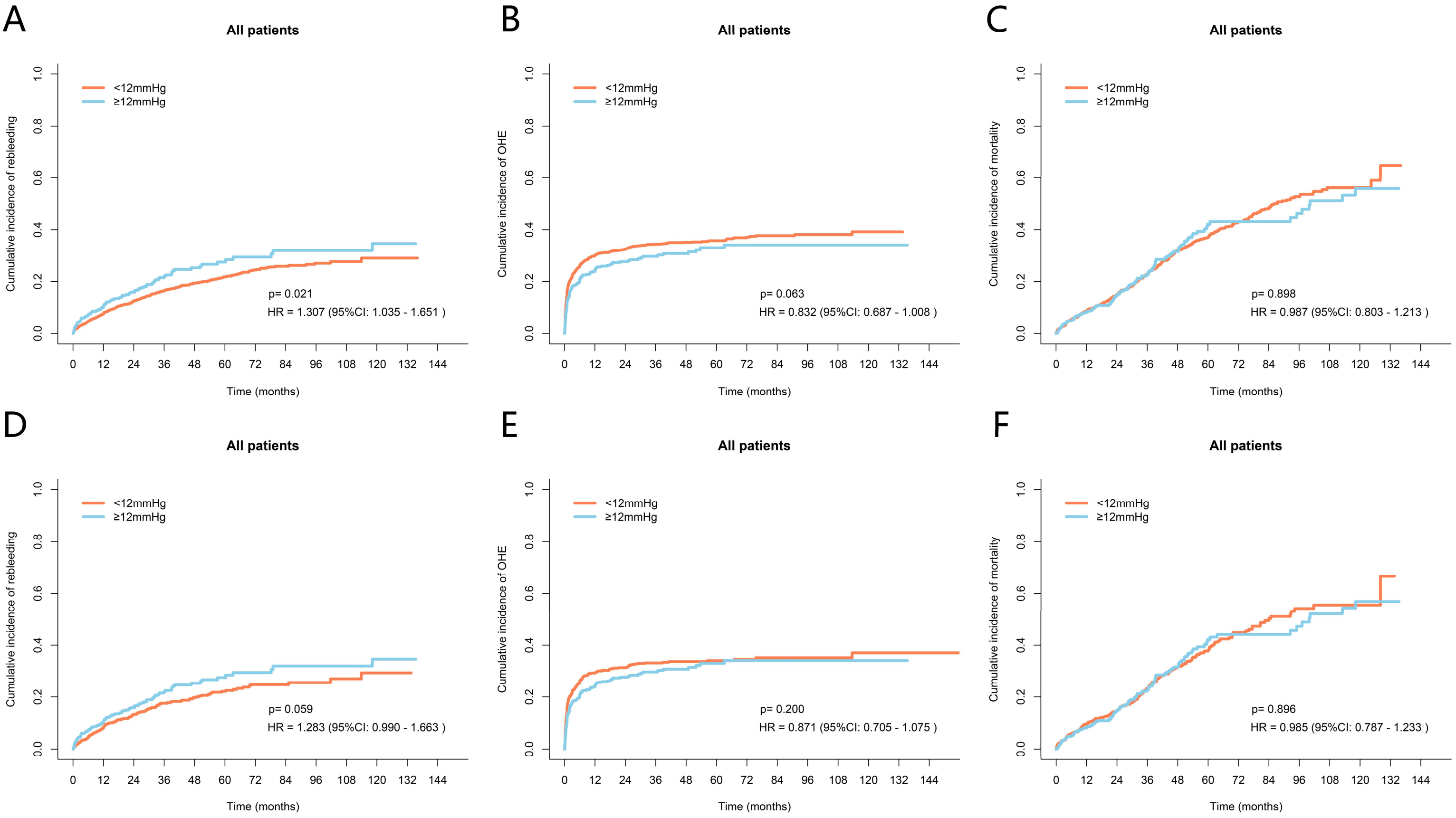

Supplementary Figure 3 Cumulative incidence of outcomes in all patients with 12mmHg post-TIPS PPG before and after PSM by competing risk analysis (Gray-Fine test).

(A) Cumulative incidence of rebleeding in all patients with 12mmHg threshold before PSM. (B) Cumulative incidence of OHE in all patients with 12mmHg threshold before PSM. (C) Cumulative incidence of mortality of all patients with 12mmHg threshold before PSM. (D) Cumulative incidence of rebleeding in all patients with 12mmHg threshold after PSM. (E) Cumulative incidence of OHE in all patients with 12mmHg threshold after PSM. (F) Cumulative incidence of mortality of all patients with 12mmHg threshold after PSM.

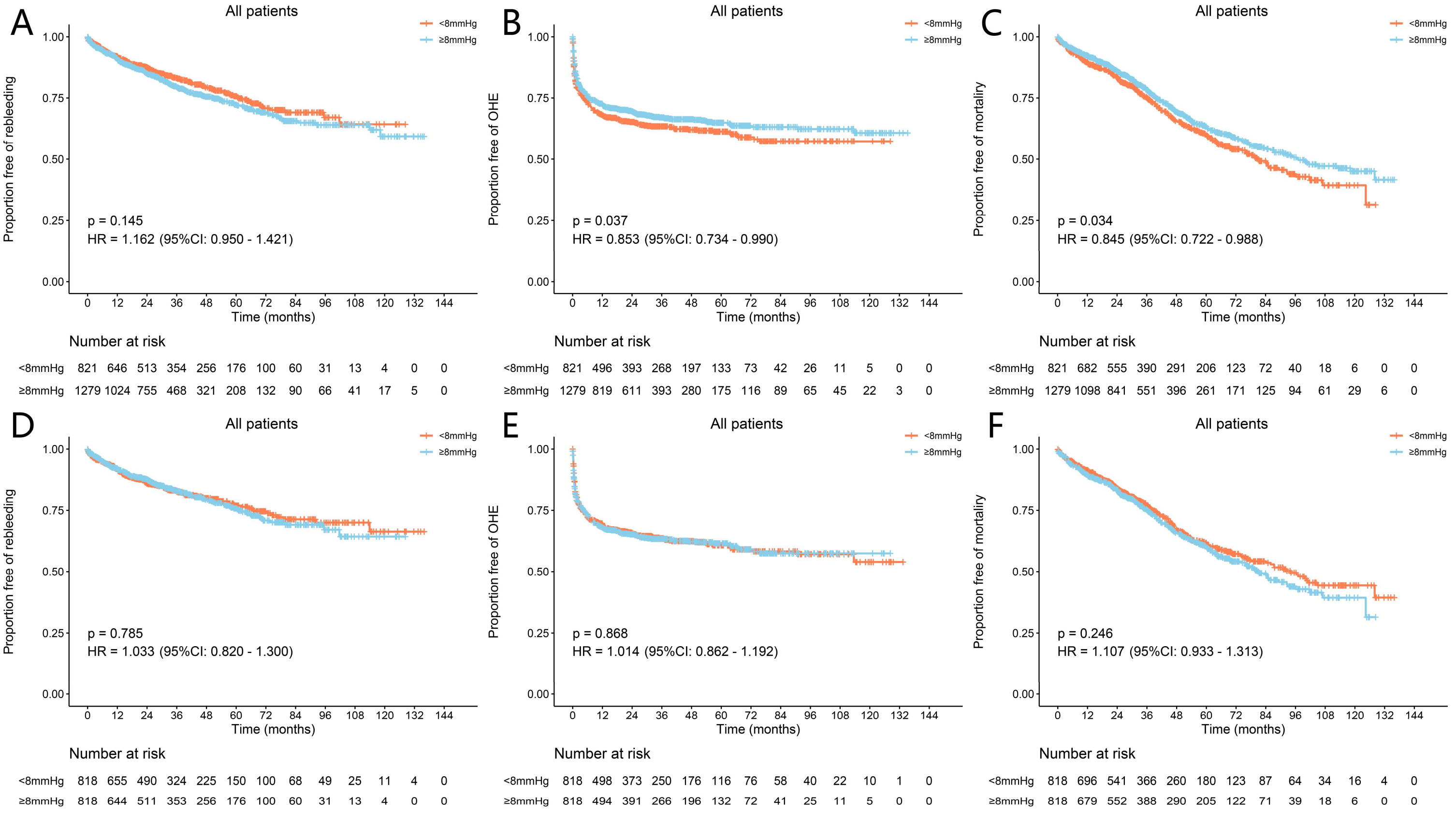

Supplementary Figure 4 Proportion free of outcomes in all patients with 8mmHg post-TIPS PPG before and after PSM.

(A) Proportion free of rebleeding in all patients with 8mmHg threshold before PSM. (B) Proportion free of OHE in all patients with 8mmHg threshold before PSM. (C) Proportion free of mortality of all patients with 8mmHg threshold before PSM. (D) Proportion free of rebleeding in all patients with 8mmHg threshold after PSM. (E) Proportion free of OHE in all patients with 8mmHg threshold after PSM. (F) Proportion free of mortality of all patients with 8mmHg threshold after PSM.

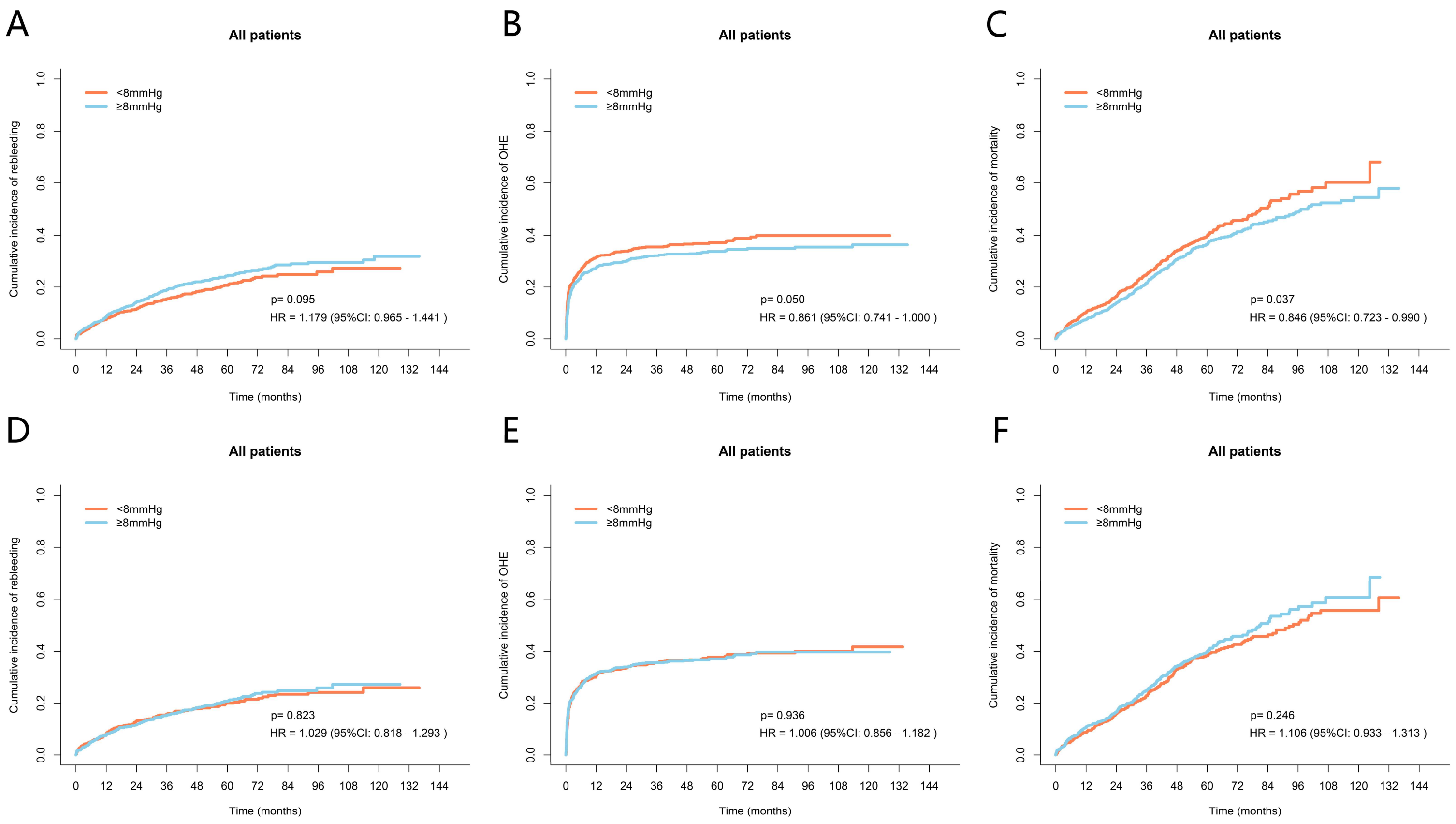

Supplementary Figure 5 Cumulative incidence of outcomes in all patients with 8mmHg post-TIPS PPG before and after PSM by competing risk analysis (Gray-Fine test).

(A) Cumulative incidence of rebleeding in all patients with 8mmHg threshold before PSM. (B) Cumulative incidence of OHE in all patients with 8mmHg threshold before PSM. (C) Cumulative incidence of mortality of all patients with 8mmHg threshold before PSM. (D) Cumulative incidence of rebleeding in all patients with 8mmHg threshold after PSM. (E) Cumulative incidence of OHE in all patients with 8mmHg threshold after PSM. (F) Cumulative incidence of mortality of all patients with 8mmHg threshold after PSM.

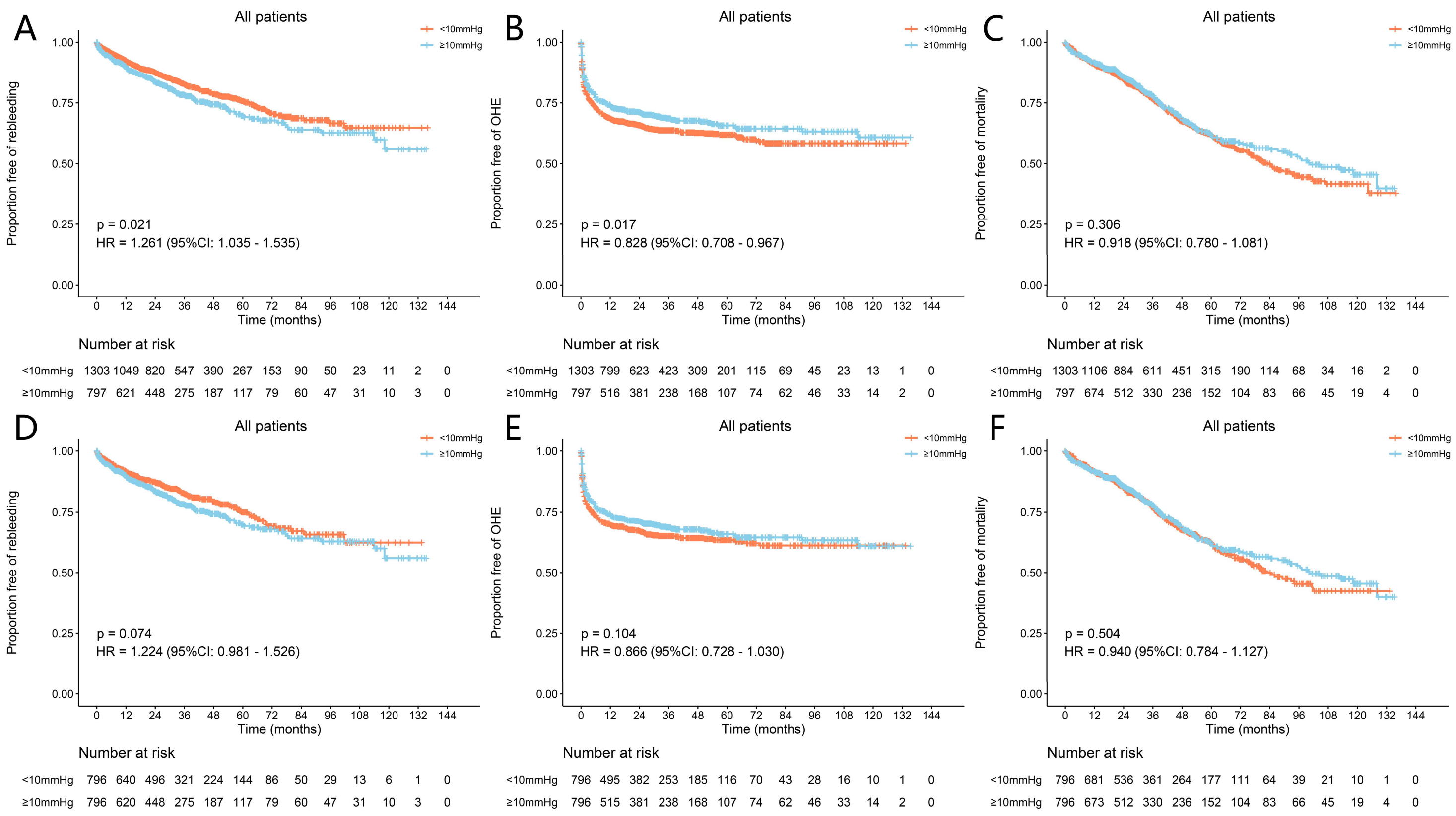

Supplementary Figure 6 Proportion free of outcomes in all patients with 10mmHg post-TIPS PPG before and after PSM.

(A) Proportion free of rebleeding in all patients with 10mmHg threshold before PSM. (B) Proportion free of OHE in all patients with 10mmHg threshold before PSM. (C) Proportion free of mortality of all patients with 10mmHg threshold before PSM. (D) Proportion free of rebleeding in all patients with 10mmHg threshold after PSM. (E) Proportion free of OHE in all patients with 10mmHg threshold after PSM. (F) Proportion free of mortality of all patients with 10mmHg threshold after PSM.

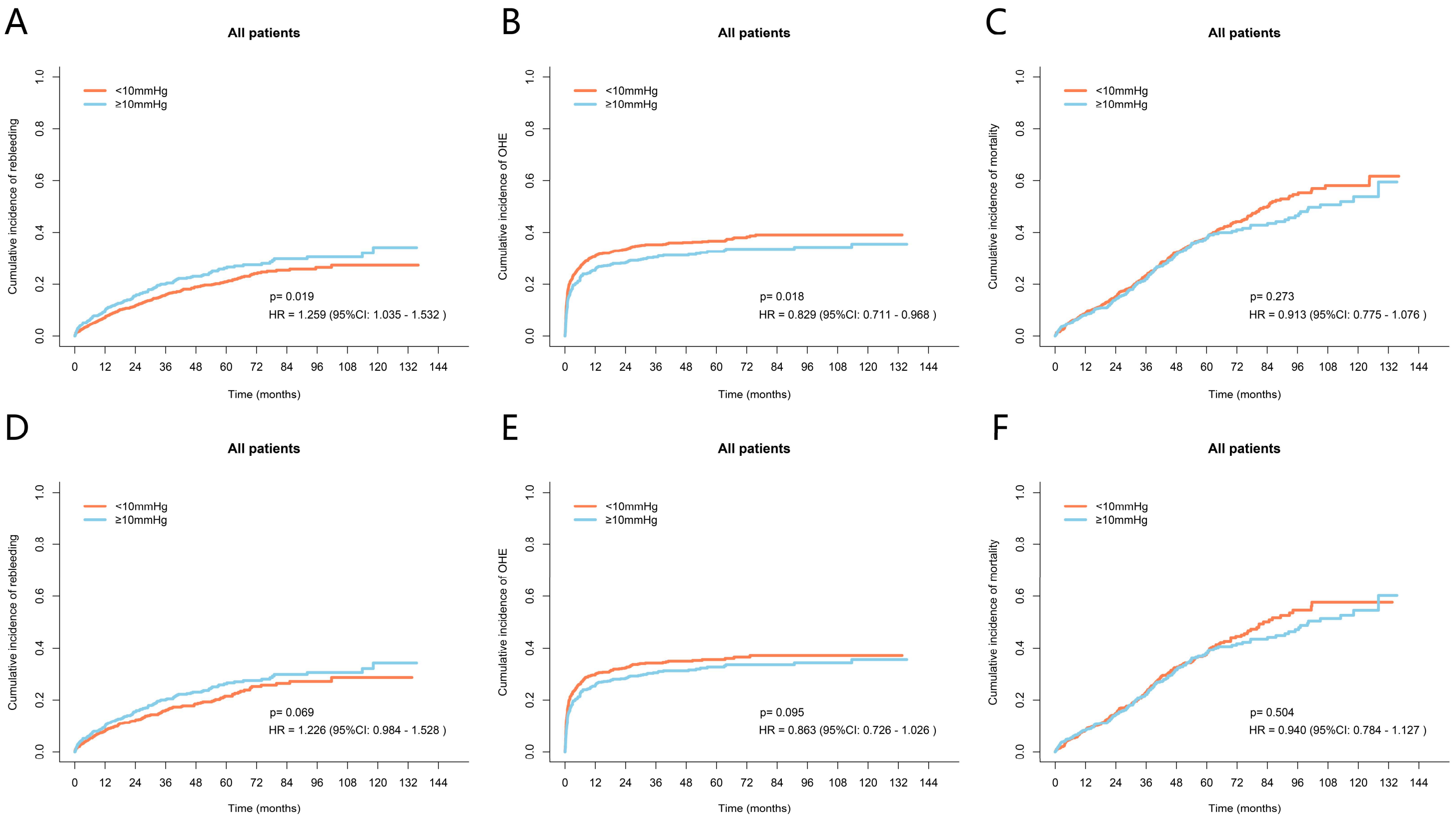

Supplementary Figure 7 Cumulative incidence of outcomes in all patients with 10mmHg post-TIPS PPG before and after PSM by competing risk analysis (Gray-Fine test).

(A) Cumulative incidence of rebleeding in all patients with 10mmHg threshold before PSM. (B) Cumulative incidence of OHE in all patients with 10mmHg threshold before PSM. (C) Cumulative incidence of mortality of all patients with 10mmHg threshold before PSM. (D) Cumulative incidence of rebleeding in all patients with 10mmHg threshold after PSM. (E) Cumulative incidence of OHE in all patients with 10mmHg threshold after PSM. (F) Cumulative incidence of mortality of all patients with 10mmHg threshold after PSM.

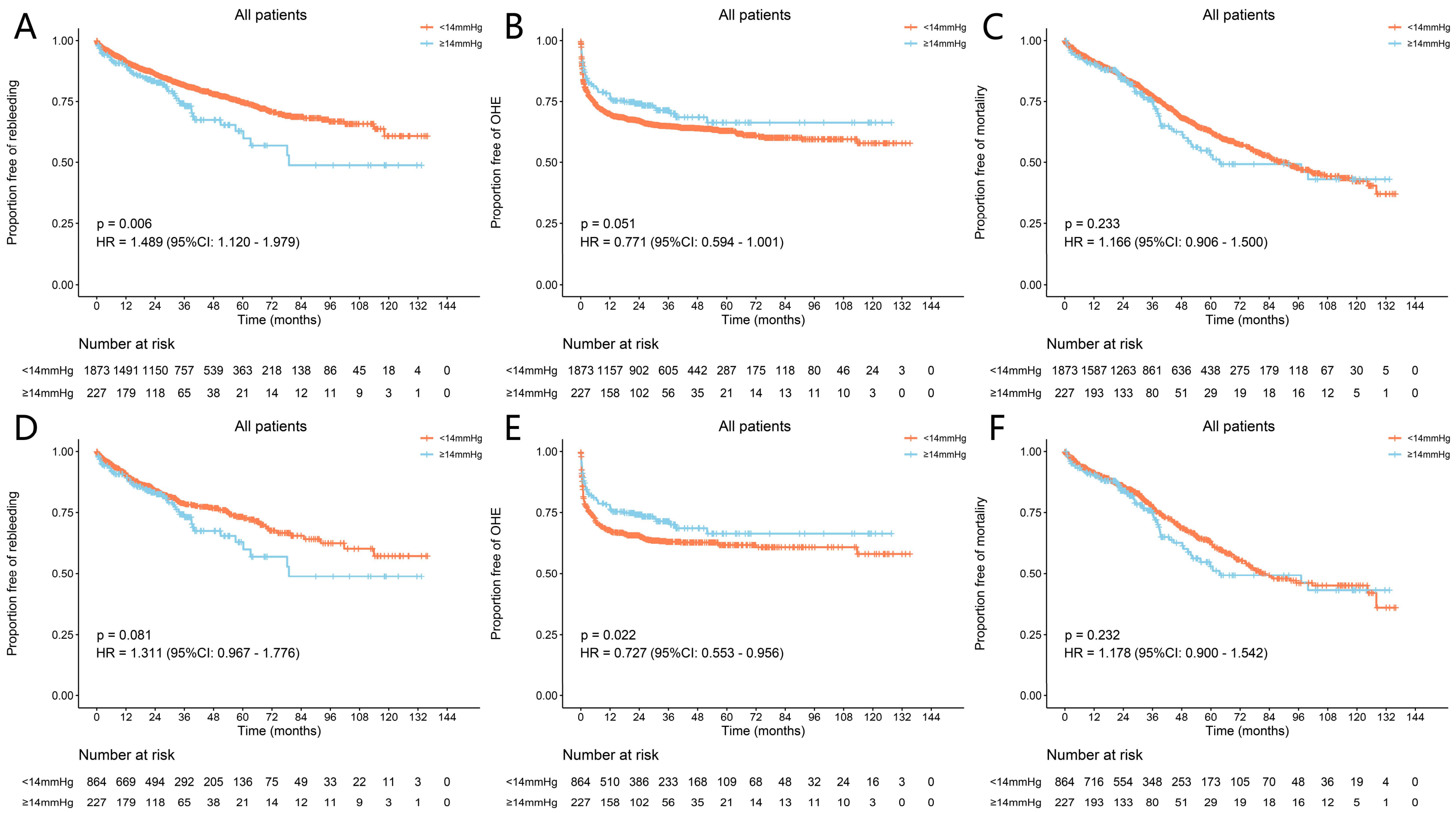

Supplementary Figure 8 Proportion free of outcomes in all patients with 14mmHg post-TIPS PPG before and after PSM.

(A) Proportion free of rebleeding in all patients with 14mmHg threshold before PSM. (B) Proportion free of OHE in all patients with 14mmHg threshold before PSM. (C) Proportion free of mortality of all patients with 14mmHg threshold before PSM. (D) Proportion free of rebleeding in all patients with 14mmHg threshold after PSM. (E) Proportion free of OHE in all patients with 14mmHg threshold after PSM. (F) Proportion free of mortality of all patients with 14mmHg threshold after PSM.

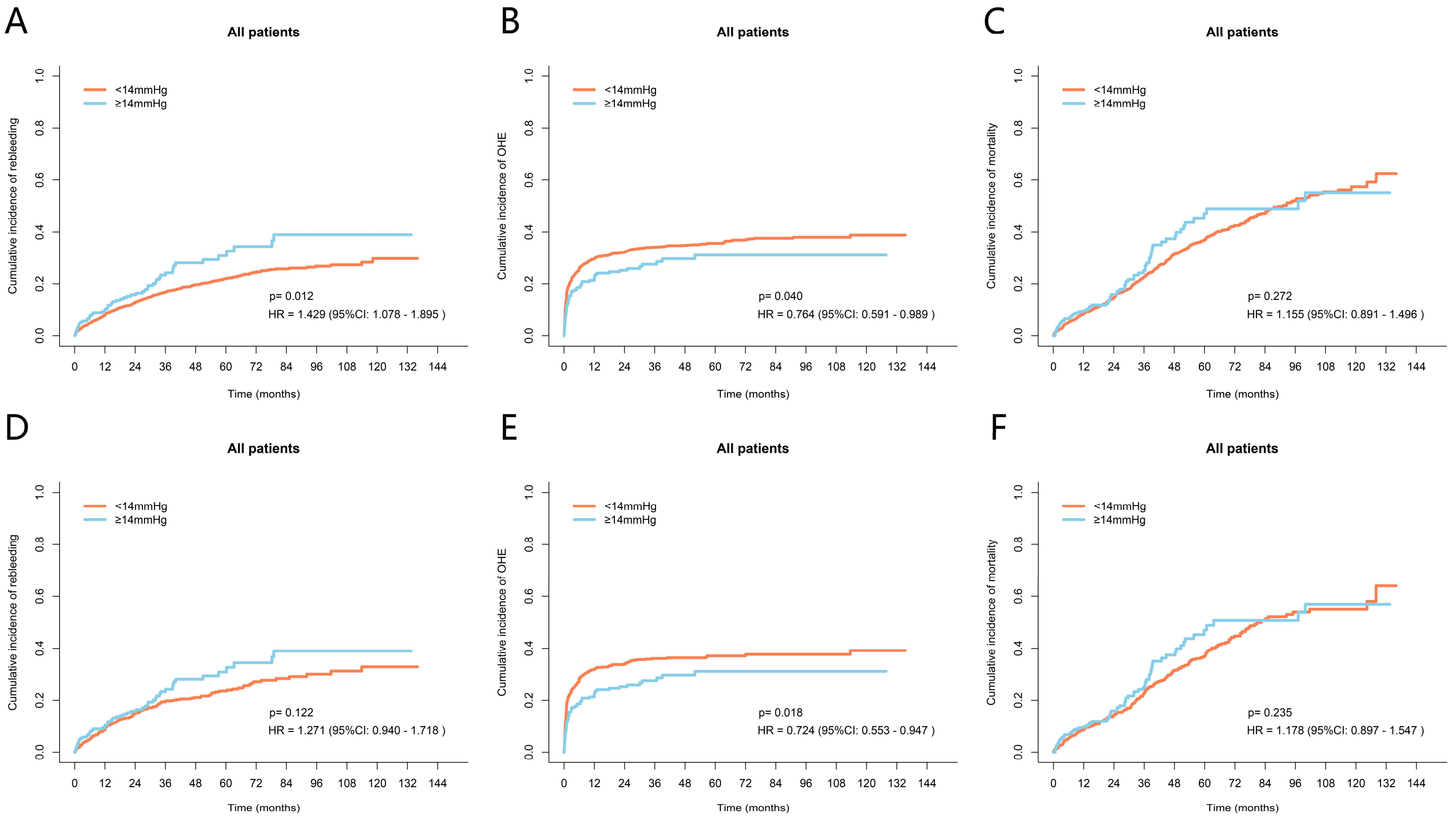

Supplementary Figure 9 Cumulative incidence of outcomes in all patients with 14mmHg post-TIPS PPG before and after PSM by competing risk analysis (Gray-Fine test).

(A) Cumulative incidence of rebleeding in all patients with 14mmHg threshold before PSM. (B) Cumulative incidence of OHE in all patients with 14mmHg threshold before PSM. (C) Cumulative incidence of mortality of all patients with 14mmHg threshold before PSM. (D) Cumulative incidence of rebleeding in all patients with 14mmHg threshold after PSM. (E) Cumulative incidence of OHE in all patients with 14mmHg threshold after PSM. (F) Cumulative incidence of mortality of all patients with 14mmHg threshold after PSM.

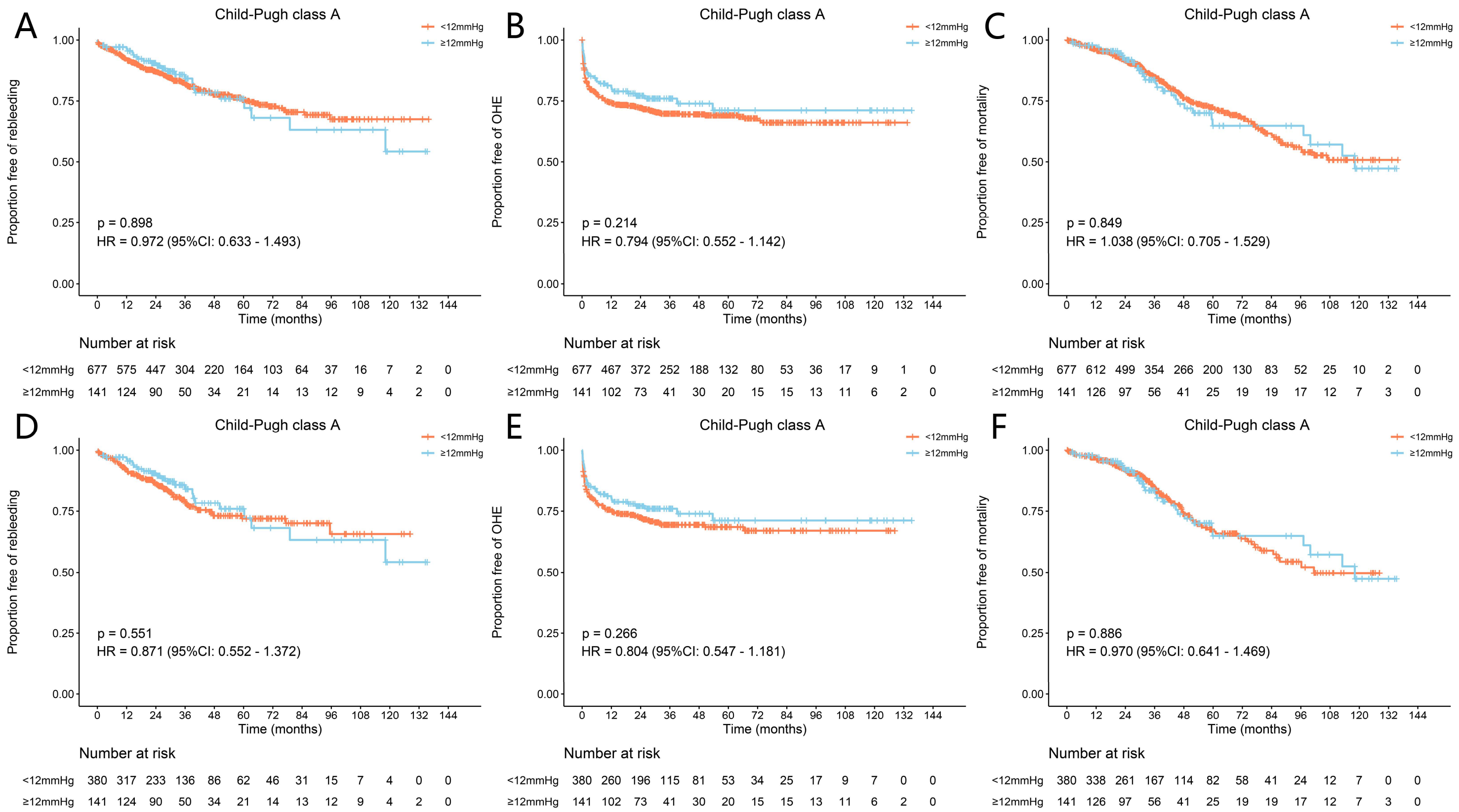

Supplementary Figure 10 Proportion free of outcomes in Child-Pugh class A with 12mmHg post-TIPS PPG before and after PSM.

(A) Proportion free of rebleeding in Child-Pugh class A with 12mmHg threshold before PSM. (B) Proportion free of OHE in Child-Pugh class A with 12mmHg threshold before PSM. (C) Proportion free of mortality of Child-Pugh class A with 12mmHg threshold before PSM. (D) Proportion free of rebleeding in Child-Pugh class A with 12mmHg threshold after PSM. (E) Proportion free of OHE in Child-Pugh class A with 12mmHg threshold after PSM. (F) Proportion free of mortality of Child-Pugh class A with 12mmHg threshold after PSM.

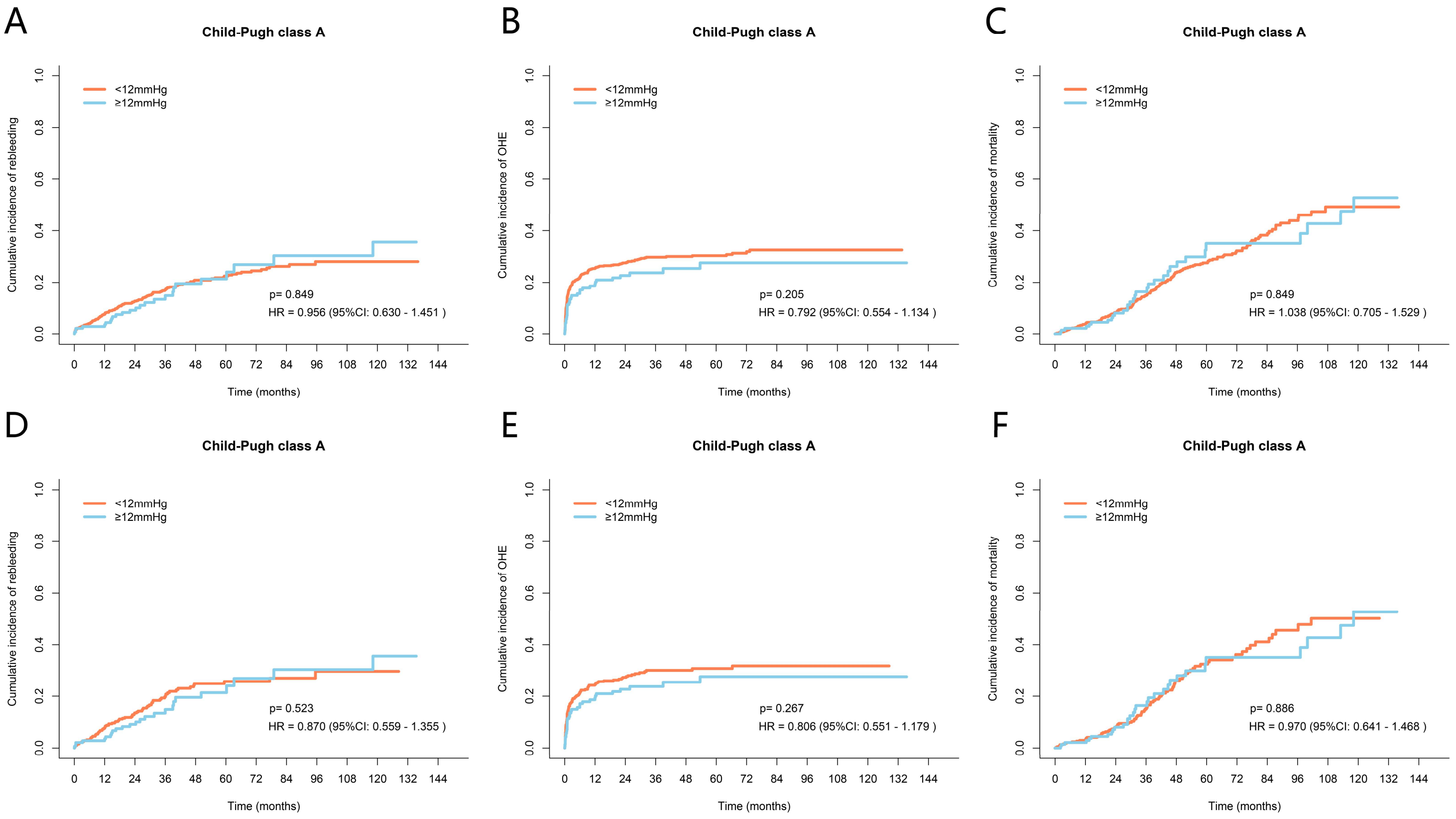

Supplementary Figure 11 Cumulative incidence of outcomes in Child-Pugh class A with 12mmHg post-TIPS PPG before and after PSM by competing risk analysis (Gray-Fine test).

(A) Cumulative incidence of rebleeding in Child-Pugh class A with 12mmHg threshold before PSM. (B) Cumulative incidence of OHE in Child-Pugh class A with 12mmHg threshold before PSM. (C) Cumulative incidence of mortality of Child-Pugh class A with 12mmHg threshold before PSM. (D) Cumulative incidence of rebleeding in Child-Pugh class A with 12mmHg threshold after PSM. (E) Cumulative incidence of OHE in Child-Pugh class A with 12mmHg threshold after PSM. (F) Cumulative incidence of mortality of Child-Pugh class A with 12mmHg threshold after PSM.

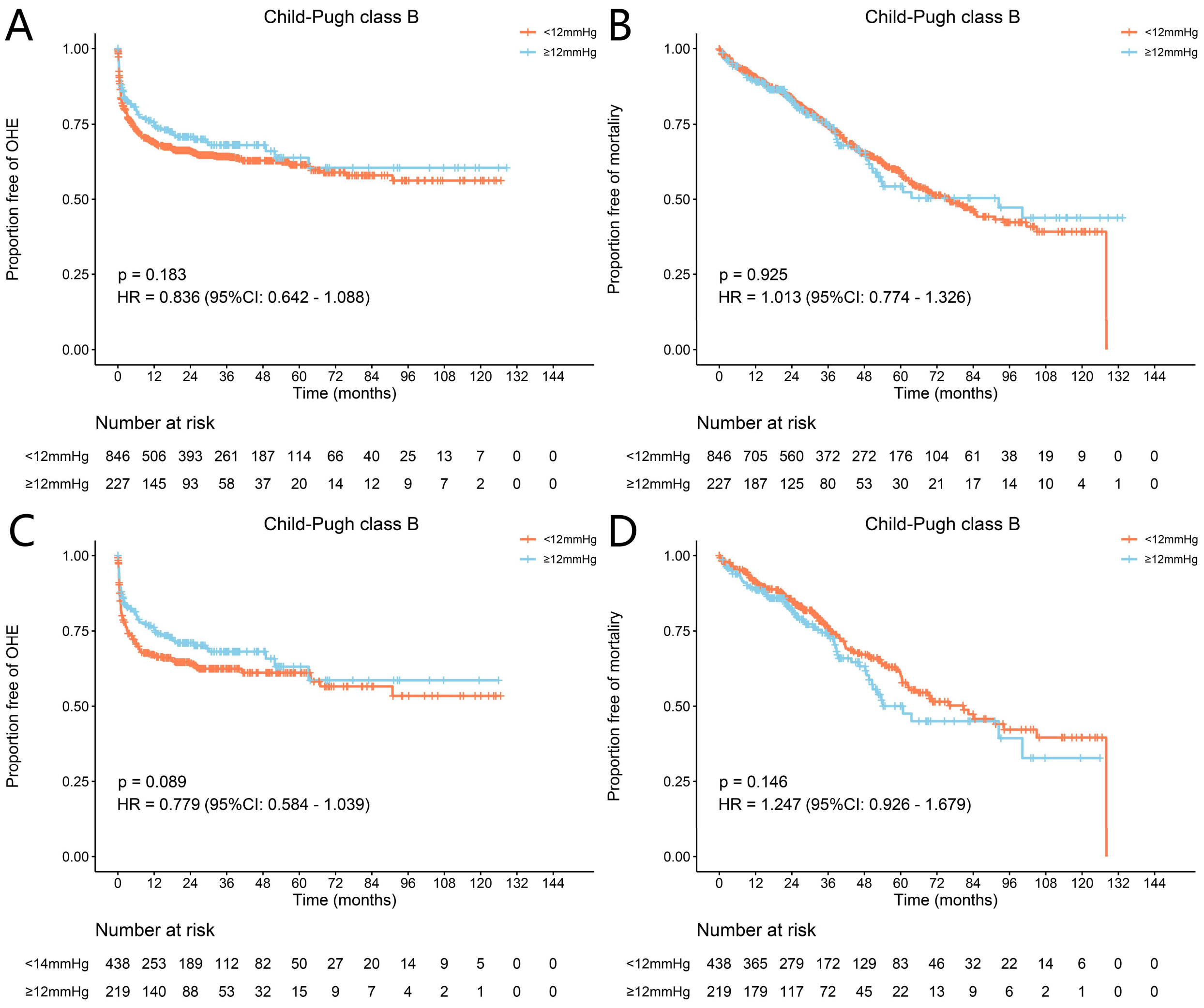

Supplementary Figure 12 Proportion free of OHE and mortality in Child-Pugh class B with 12mmHg post-TIPS PPG before and after PSM.

(A) Proportion free of OHE in Child-Pugh class B with 12mmHg threshold before PSM. (B) Proportion free of mortality of Child-Pugh class B with 12mmHg threshold before PSM. (C) Proportion free of OHE in Child-Pugh class B with 12mmHg threshold after PSM. (D) Proportion free of mortality of Child-Pugh class B with 12mmHg threshold after PSM.

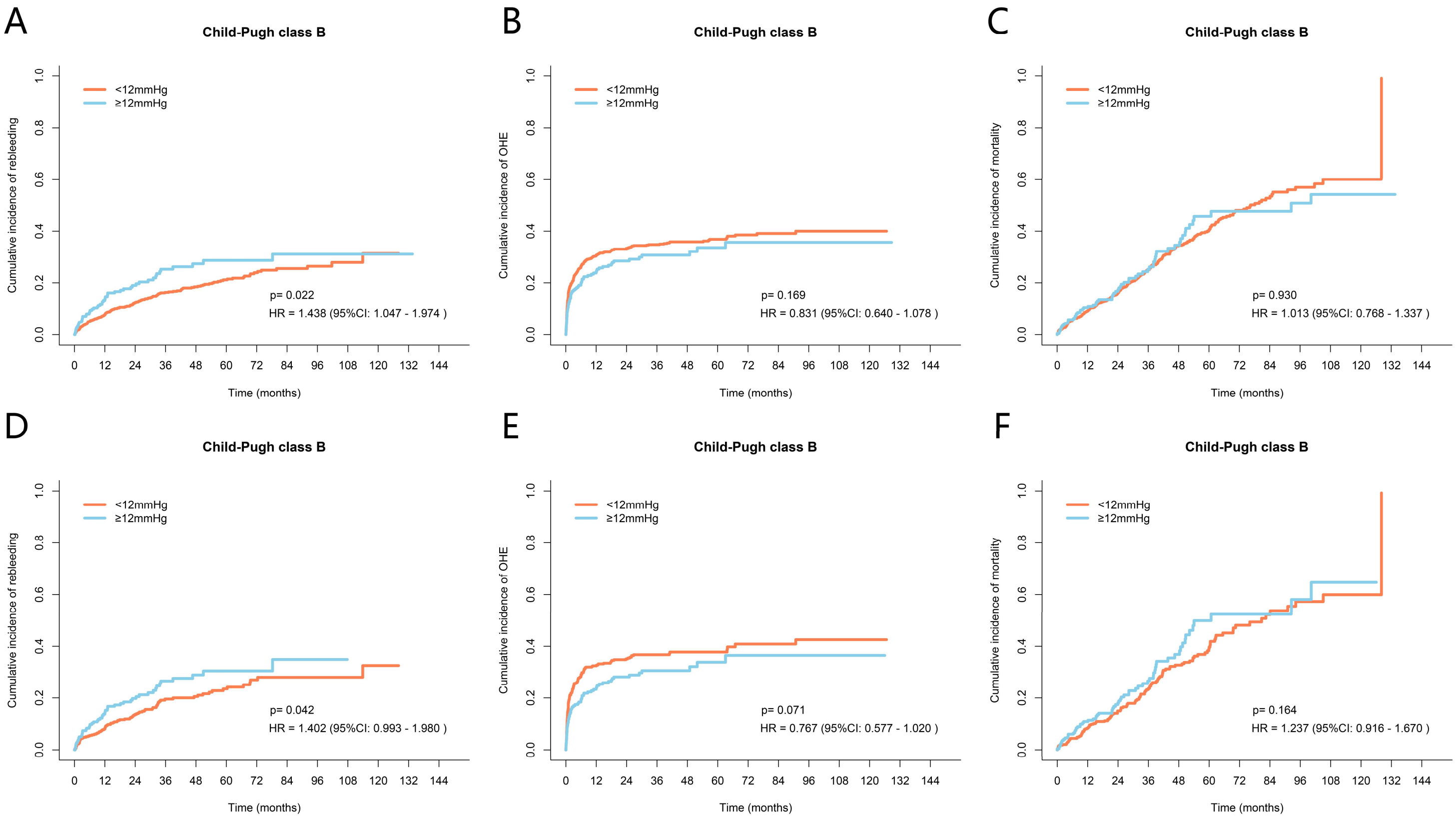

Supplementary Figure 13 Cumulative incidence of outcomes in Child-Pugh class B with 12mmHg post-TIPS PPG before and after PSM by competing risk analysis (Gray-Fine test).

(A) Cumulative incidence of rebleeding in Child-Pugh class B with 12mmHg threshold before PSM. (B) Cumulative incidence of OHE in Child-Pugh class B with 12mmHg threshold before PSM. (C) Cumulative incidence of mortality of Child-Pugh class B with 12mmHg threshold before PSM. (D) Cumulative incidence of rebleeding in Child-Pugh class B with 12mmHg threshold after PSM. (E) Cumulative incidence of OHE in Child-Pugh class B with 12mmHg threshold after PSM. (F) Cumulative incidence of mortality of Child-Pugh class B with 12mmHg threshold after PSM.

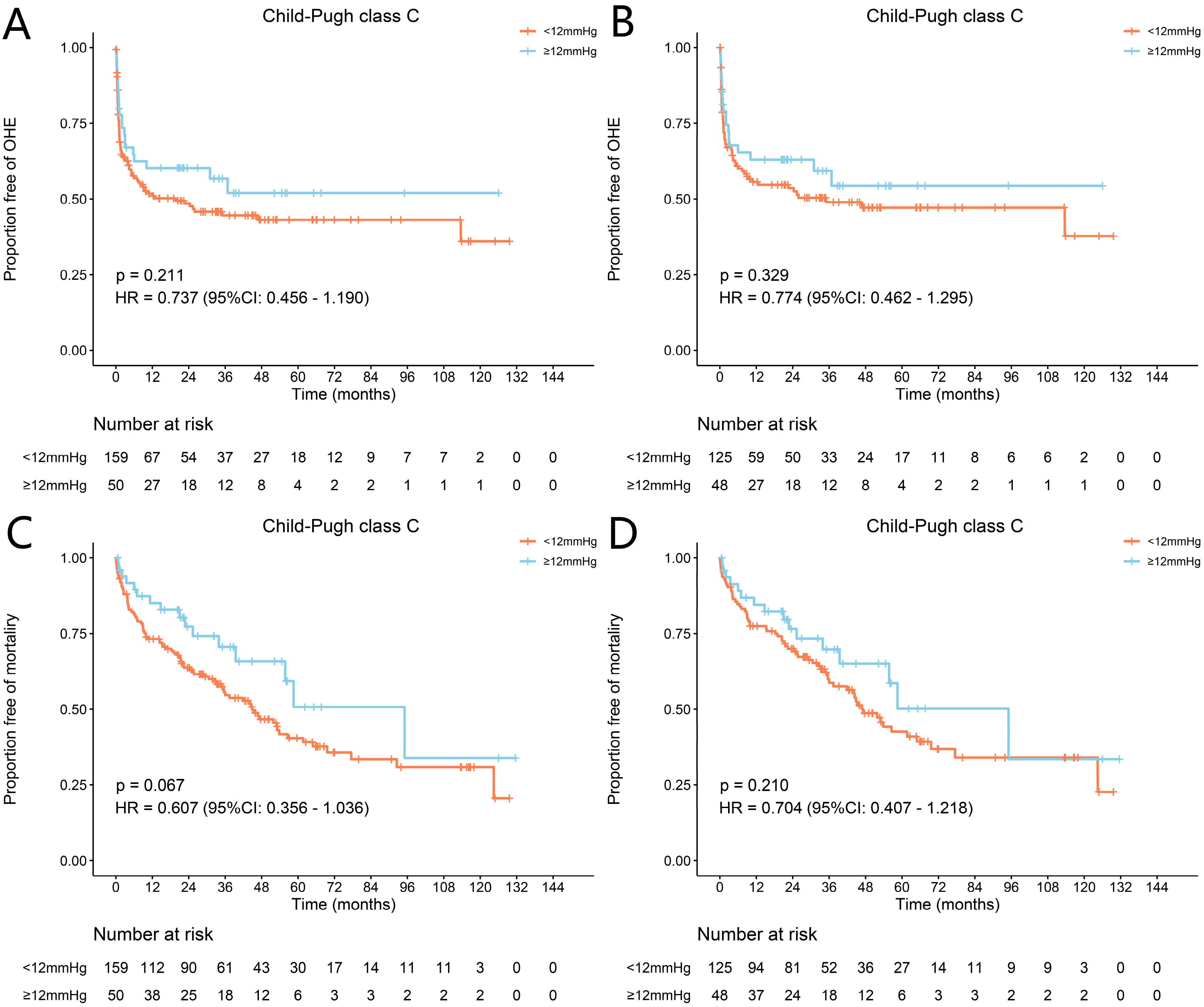

Supplementary Figure 14 Proportion free of OHE and mortality in Child-Pugh class C with 12mmHg post-TIPS PPG before and after PSM.

(A) Proportion free of OHE in Child-Pugh class C with 12mmHg threshold before PSM. (B) Proportion free of OHE in Child-Pugh class C with 12mmHg threshold after PSM. (C) Proportion free of mortality of Child-Pugh class C with 12mmHg threshold before PSM. (D) Proportion free of mortality of Child-Pugh class C with 12mmHg threshold after PSM.

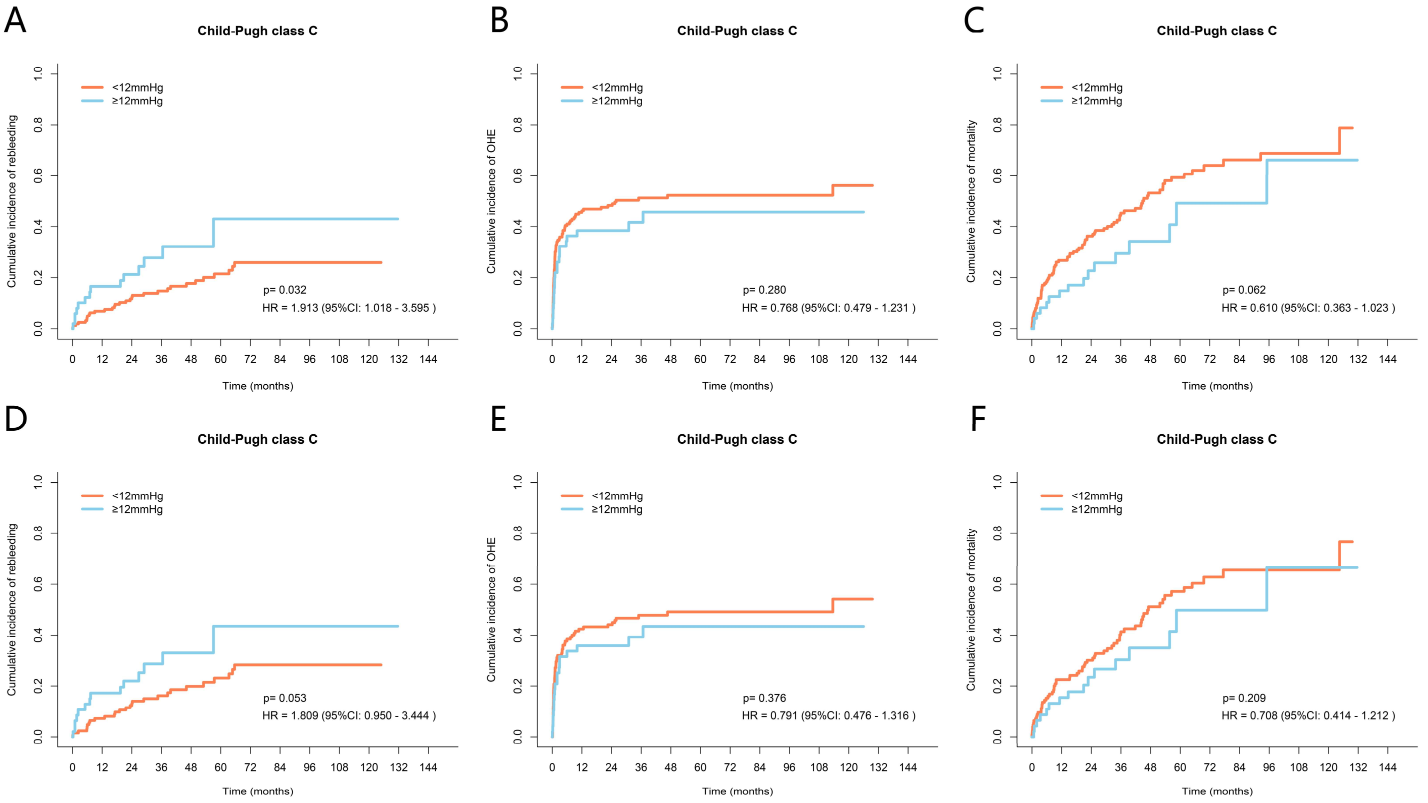

Supplementary Figure 15 Cumulative incidence of outcomes in Child-Pugh class C with 12mmHg post-TIPS PPG before and after PSM by competing risk analysis (Gray-Fine test).

(A) Cumulative incidence of rebleeding in Child-Pugh class C with 12mmHg threshold before PSM. (B) Cumulative incidence of OHE in Child-Pugh class C with 12mmHg threshold before PSM. (C) Cumulative incidence of mortality of Child-Pugh class C with 12mmHg threshold before PSM. (D) Cumulative incidence of rebleeding in Child-Pugh class C with 12mmHg threshold after PSM. (E) Cumulative incidence of OHE in Child-Pugh class C with 12mmHg threshold after PSM. (F) Cumulative incidence of mortality of Child-Pugh class C with 12mmHg threshold after PSM.

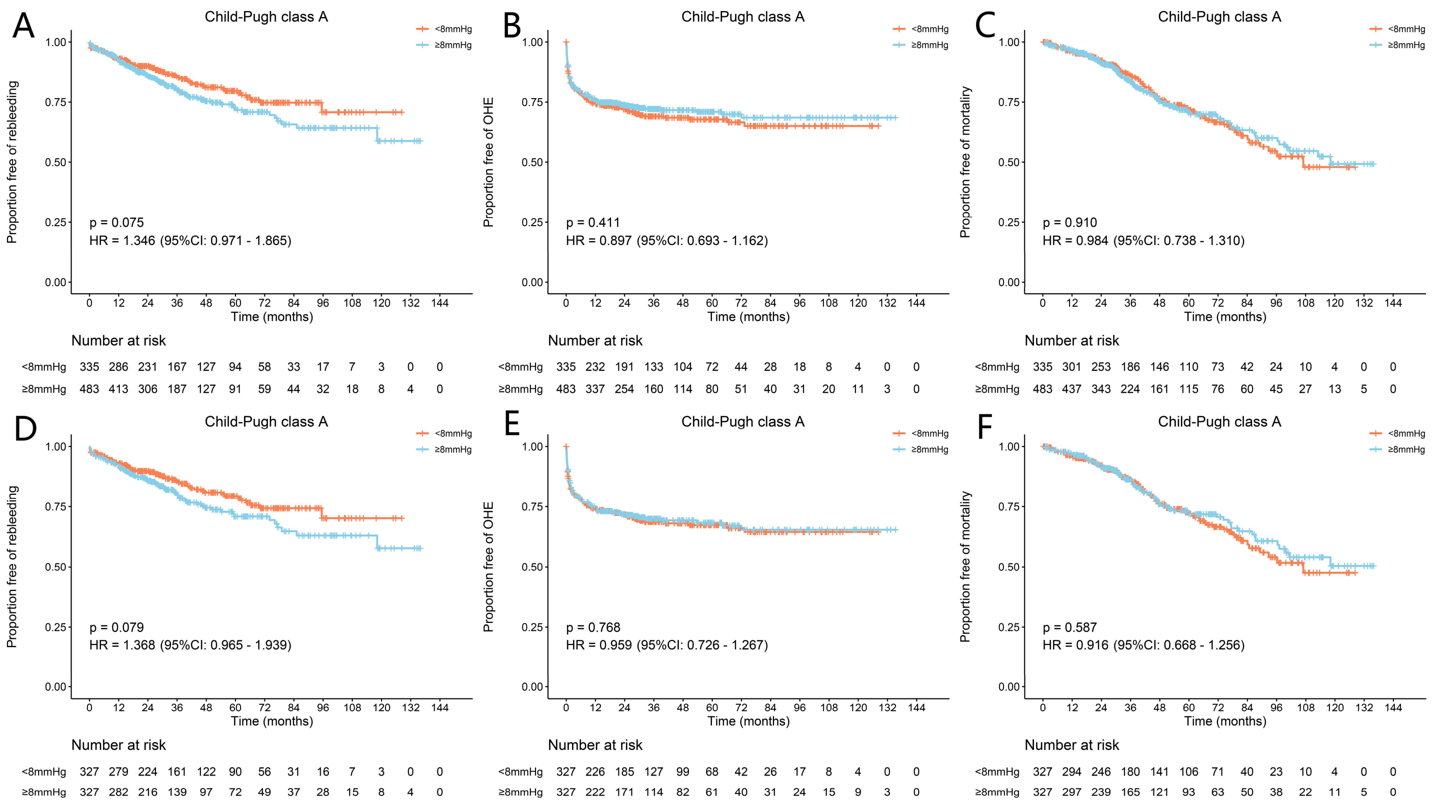

Supplementary Figure 16 Proportion free of outcomes in Child-Pugh class A with 8mmHg post-TIPS PPG before and after PSM.

(A) Proportion free of rebleeding in Child-Pugh class A with 8mmHg threshold before PSM. (B) Proportion free of OHE in Child-Pugh class A with 8mmHg threshold before PSM. (C) Proportion free of mortality of Child-Pugh class A with 8mmHg threshold before PSM. (D) Proportion free of rebleeding in Child-Pugh class A with 8mmHg threshold after PSM. (E) Proportion free of OHE in Child-Pugh class A with 8mmHg threshold after PSM. (F) Proportion free of mortality of Child-Pugh class A with 8mmHg threshold after PSM.

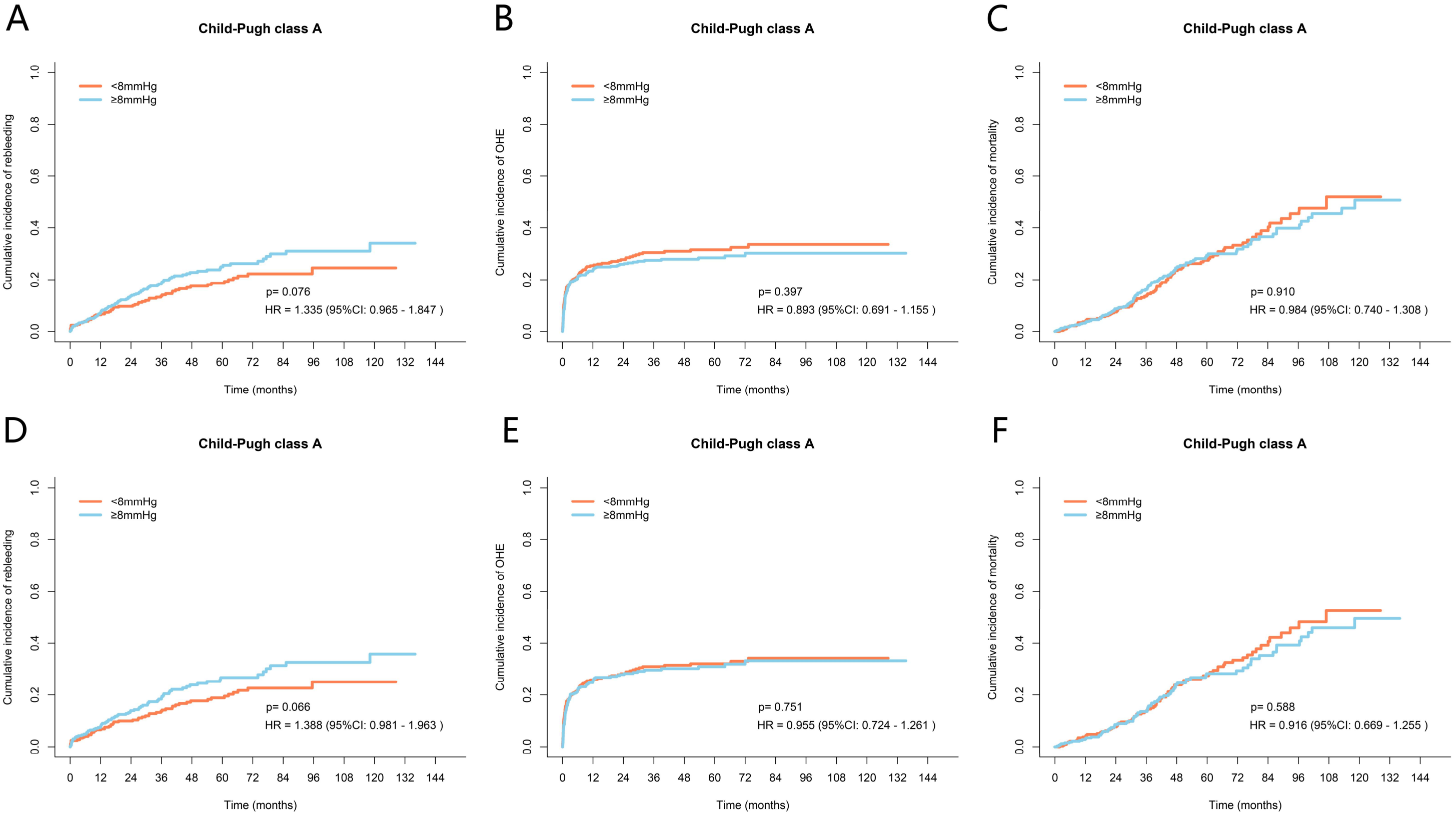

Supplementary Figure 17 Cumulative incidence of outcomes in Child-Pugh class A with 8mmHg post-TIPS PPG before and after PSM by competing risk analysis (Gray-Fine test).

(A) Cumulative incidence of rebleeding in Child-Pugh class A with 8mmHg threshold before PSM. (B) Cumulative incidence of OHE in Child-Pugh class A with 8mmHg threshold before PSM. (C) Cumulative incidence of mortality of Child-Pugh class A with 8mmHg threshold before PSM. (D) Cumulative incidence of rebleeding in Child-Pugh class A with 8mmHg threshold after PSM. (E) Cumulative incidence of OHE in Child-Pugh class A with 8mmHg threshold after PSM. (F) Cumulative incidence of mortality of Child-Pugh class A with 8mmHg threshold after PSM.

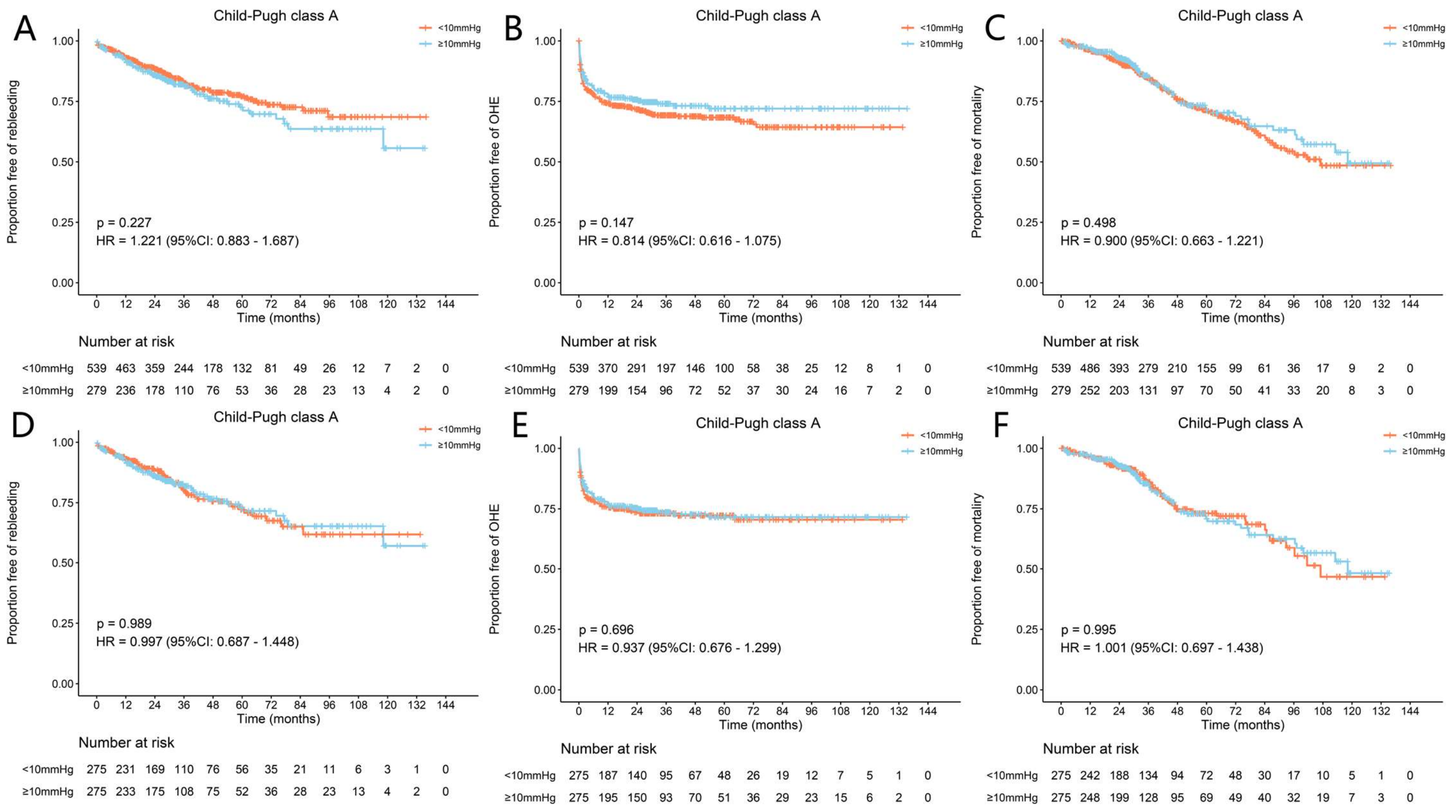

Supplementary Figure 18 Proportion free of outcomes in Child-Pugh class A with 10mmHg post-TIPS PPG before and after PSM.

(A) Proportion free of rebleeding in Child-Pugh class A with 10mmHg threshold before PSM. (B) Proportion free of OHE in Child-Pugh class A with 10mmHg threshold before PSM. (C) Proportion free of mortality of Child-Pugh class A with 10mmHg threshold before PSM. (D) Proportion free of rebleeding in Child-Pugh class A with 10mmHg threshold after PSM. (E) Proportion free of OHE in Child-Pugh class A with 10mmHg threshold after PSM. (F) Proportion free of mortality of Child-Pugh class A with 10mmHg threshold after PSM.

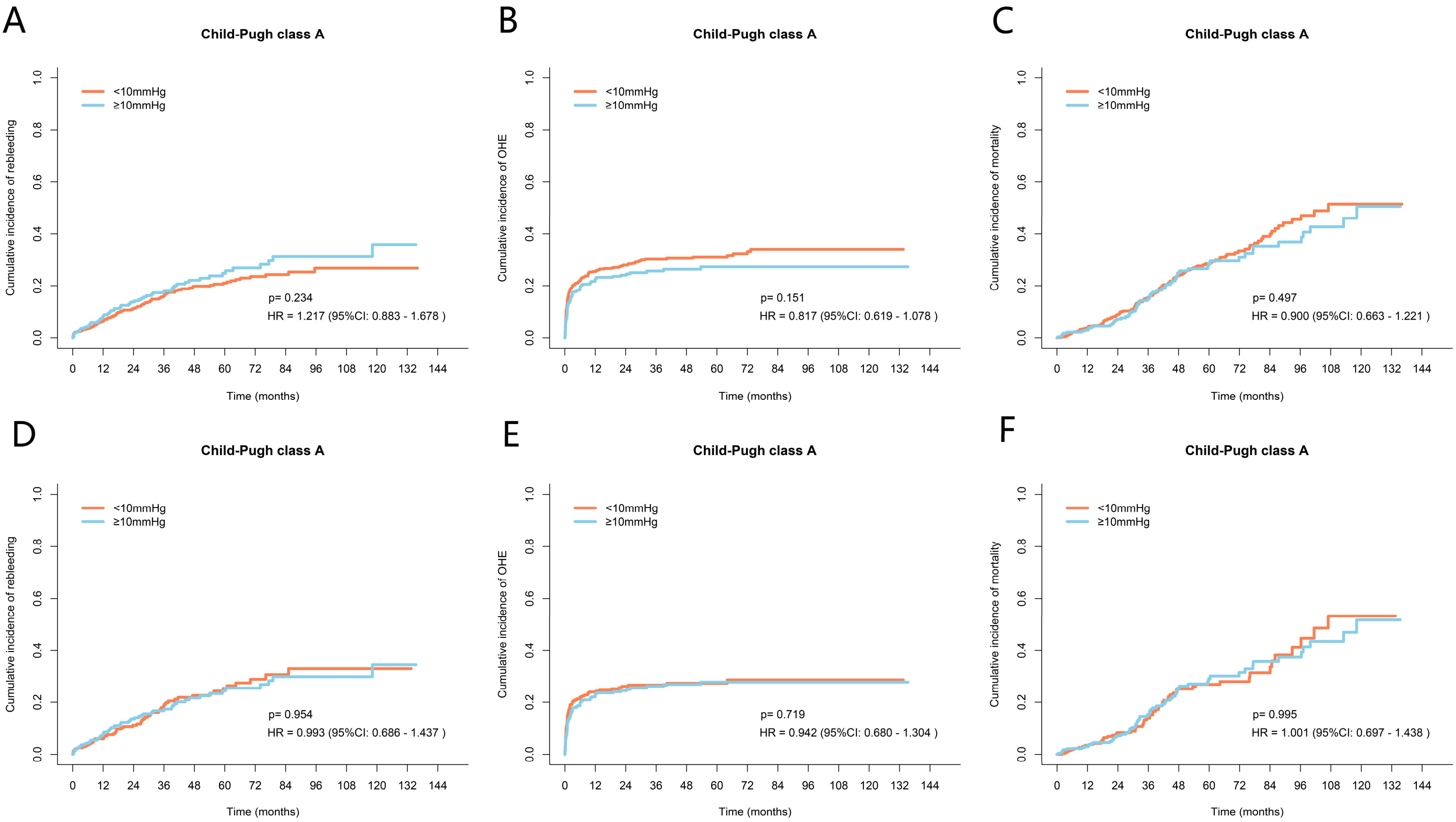

Supplementary Figure 19 Cumulative incidence of outcomes in Child-Pugh class A with 10mmHg post-TIPS PPG before and after PSM by competing risk analysis (Gray-Fine test).

(A) Cumulative incidence of rebleeding in Child-Pugh class A with 10mmHg threshold before PSM. (B) Cumulative incidence of OHE in Child-Pugh class A with 10mmHg threshold before PSM. (C) Cumulative incidence of mortality of Child-Pugh class A with 10mmHg threshold before PSM. (D) Cumulative incidence of rebleeding in Child-Pugh class A with 10mmHg threshold after PSM. (E) Cumulative incidence of OHE in Child-Pugh class A with 10mmHg threshold after PSM. (F) Cumulative incidence of mortality of Child-Pugh class A with 10mmHg threshold after PSM.

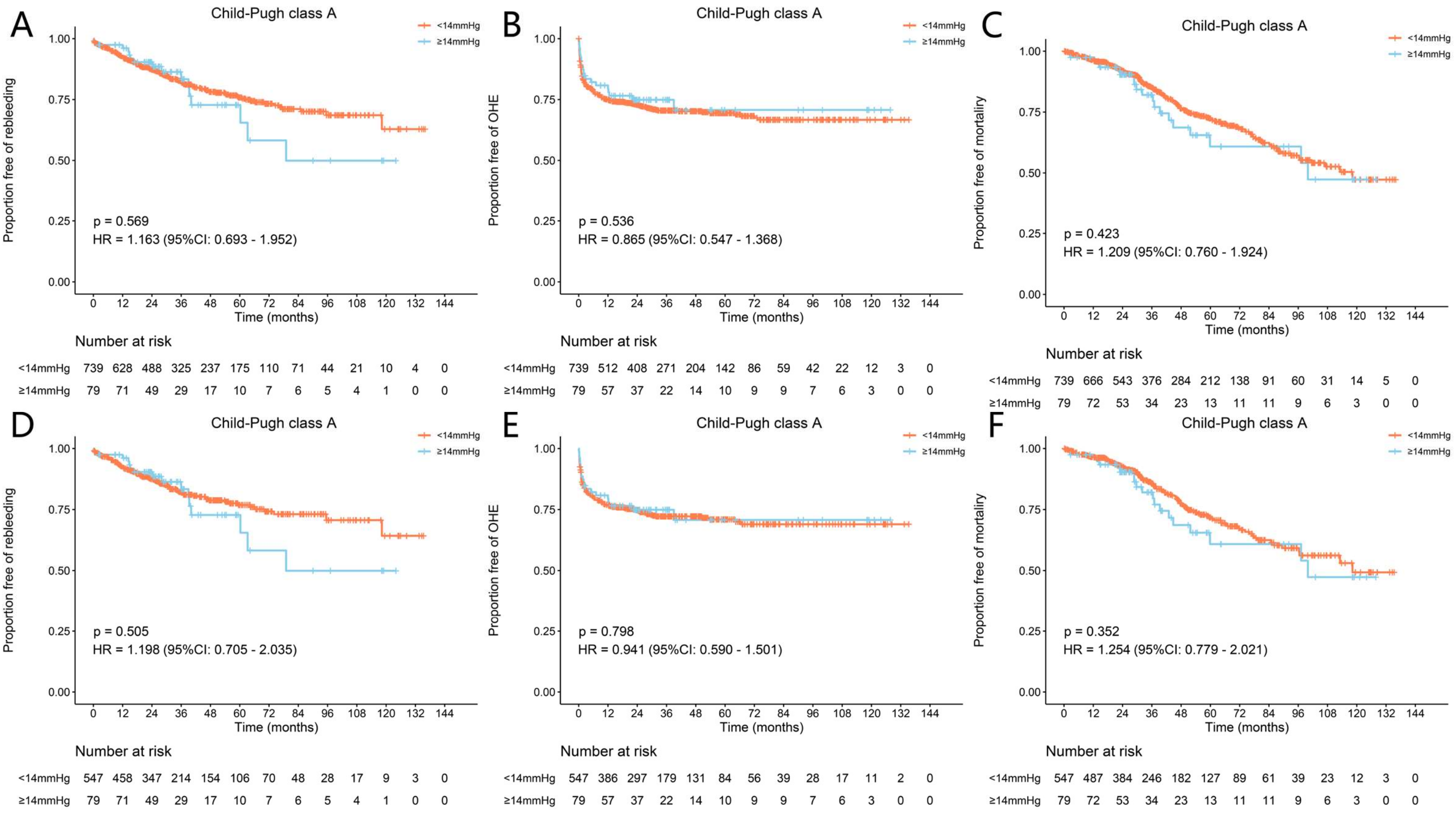

Supplementary Figure 20 Proportion free of outcomes in Child-Pugh class A with 14mmHg post-TIPS PPG before and after PSM.

(A) Proportion free of rebleeding in Child-Pugh class A with 14mmHg threshold before PSM. (B) Proportion free of OHE in Child-Pugh class A with 14mmHg threshold before PSM. (C) Proportion free of mortality of Child-Pugh class A with 14mmHg threshold before PSM. (D) Proportion free of rebleeding in Child-Pugh class A with 14mmHg threshold after PSM. (E) Proportion free of OHE in Child-Pugh class A with 14mmHg threshold after PSM. (F) Proportion free of mortality of Child-Pugh class A with 14mmHg threshold after PSM.

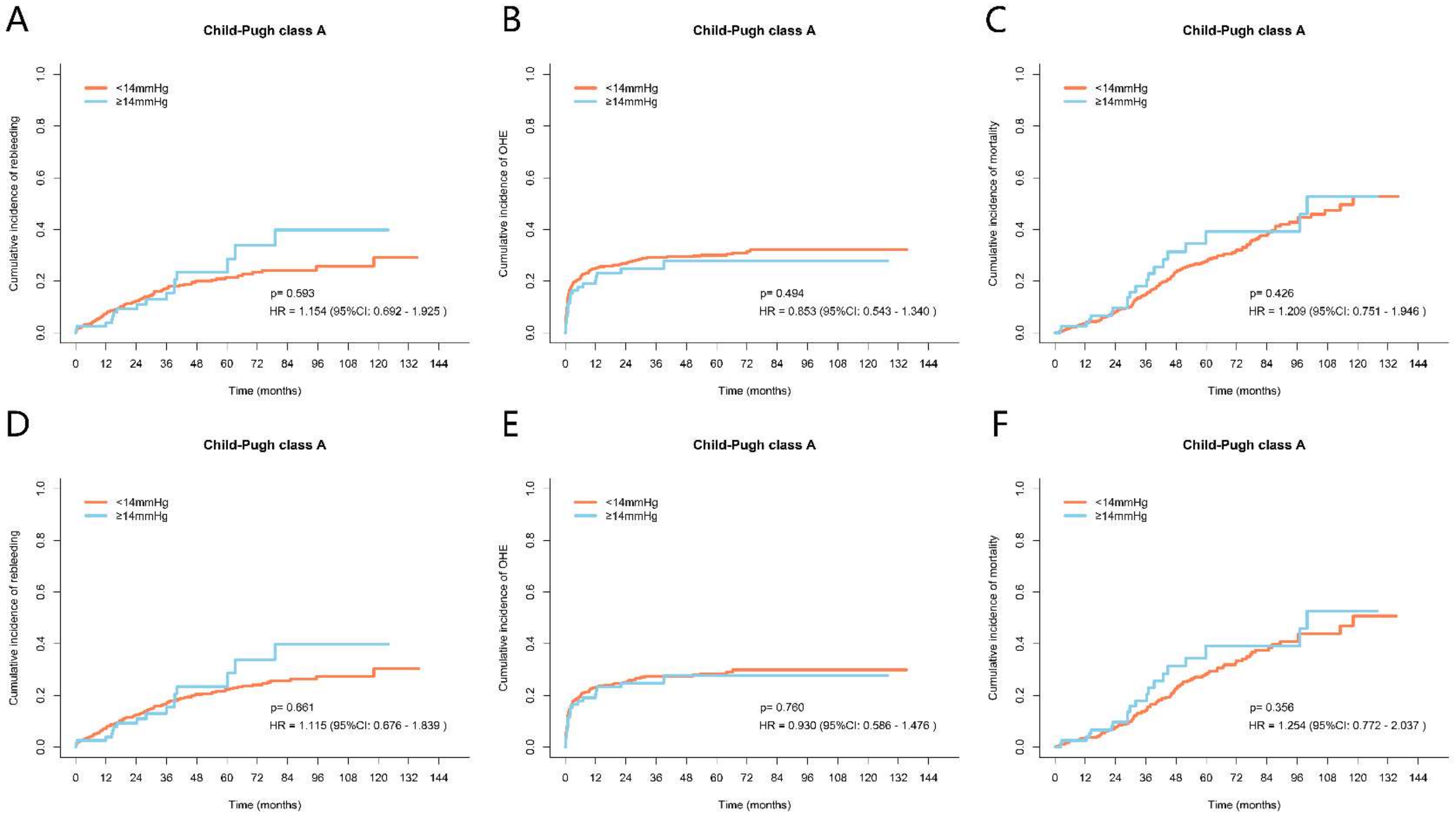

Supplementary Figure 21 Cumulative incidence of outcomes in Child-Pugh class A with 14mmHg post-TIPS PPG before and after PSM by competing risk analysis (Gray-Fine test).

(A) Cumulative incidence of rebleeding in Child-Pugh class A with 14mmHg threshold before PSM. (B) Cumulative incidence of OHE in Child-Pugh class A with 14mmHg threshold before PSM. (C) Cumulative incidence of mortality of Child-Pugh class A with 14mmHg threshold before PSM. (D) Cumulative incidence of rebleeding in Child-Pugh class A with 14mmHg threshold after PSM. (E) Cumulative incidence of OHE in Child-Pugh class A with 14mmHg threshold after PSM. (F) Cumulative incidence of mortality of Child-Pugh class A with 14mmHg threshold after PSM.

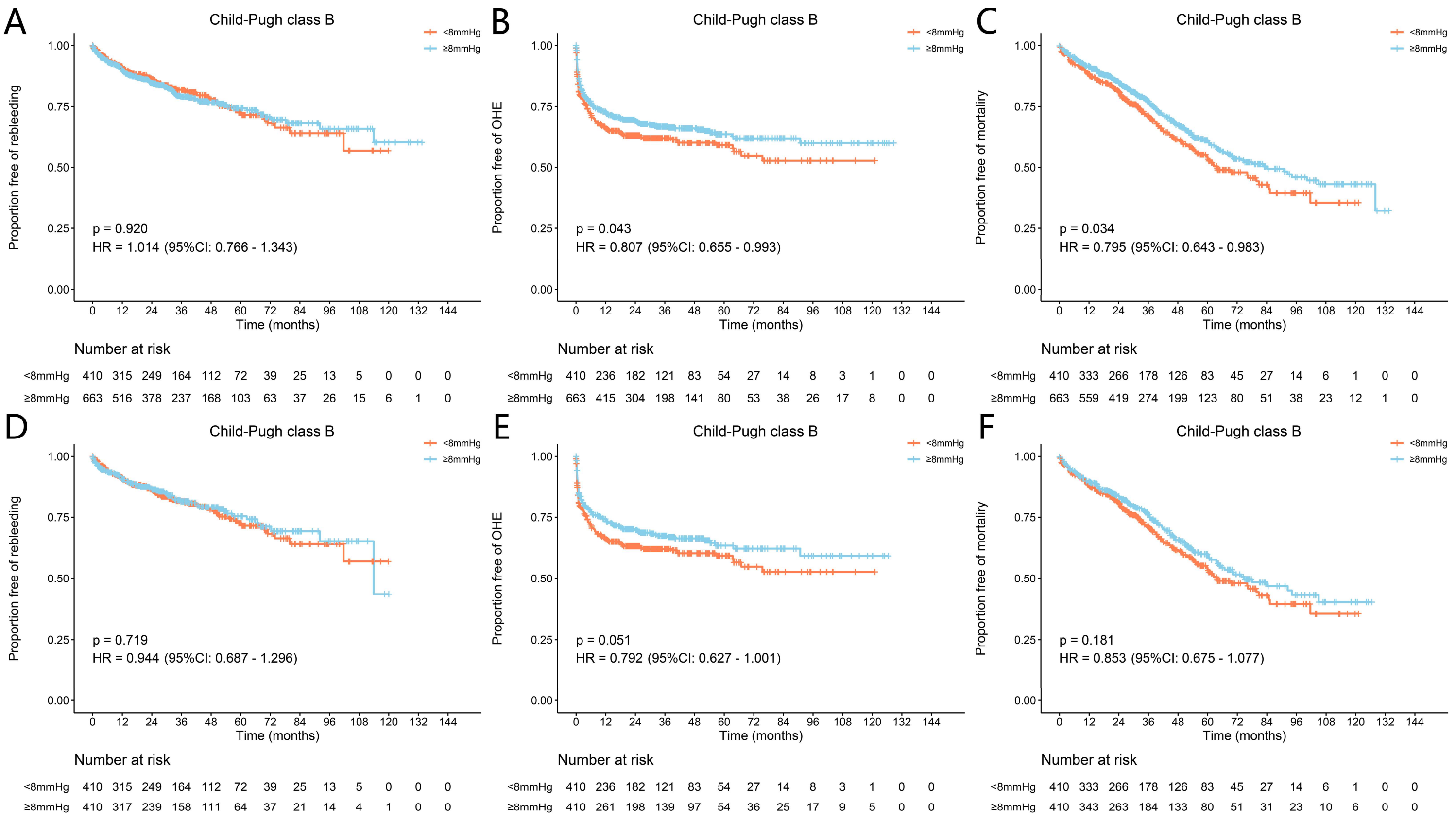

Supplementary Figure 22 Proportion free of outcomes in Child-Pugh class B with 8mmHg post-TIPS PPG before and after PSM.

(A) Proportion free of rebleeding in Child-Pugh class B with 8mmHg threshold before PSM. (B) Proportion free of OHE in Child-Pugh class B with 8mmHg threshold before PSM. (C) Proportion free of mortality of Child-Pugh class B with 8mmHg threshold before PSM. (D) Proportion free of rebleeding in Child-Pugh class B with 8mmHg threshold after PSM. (E) Proportion free of OHE in Child-Pugh class B with 8mmHg threshold after PSM. (F) Proportion free of mortality of Child-Pugh class B with 8mmHg threshold after PSM.

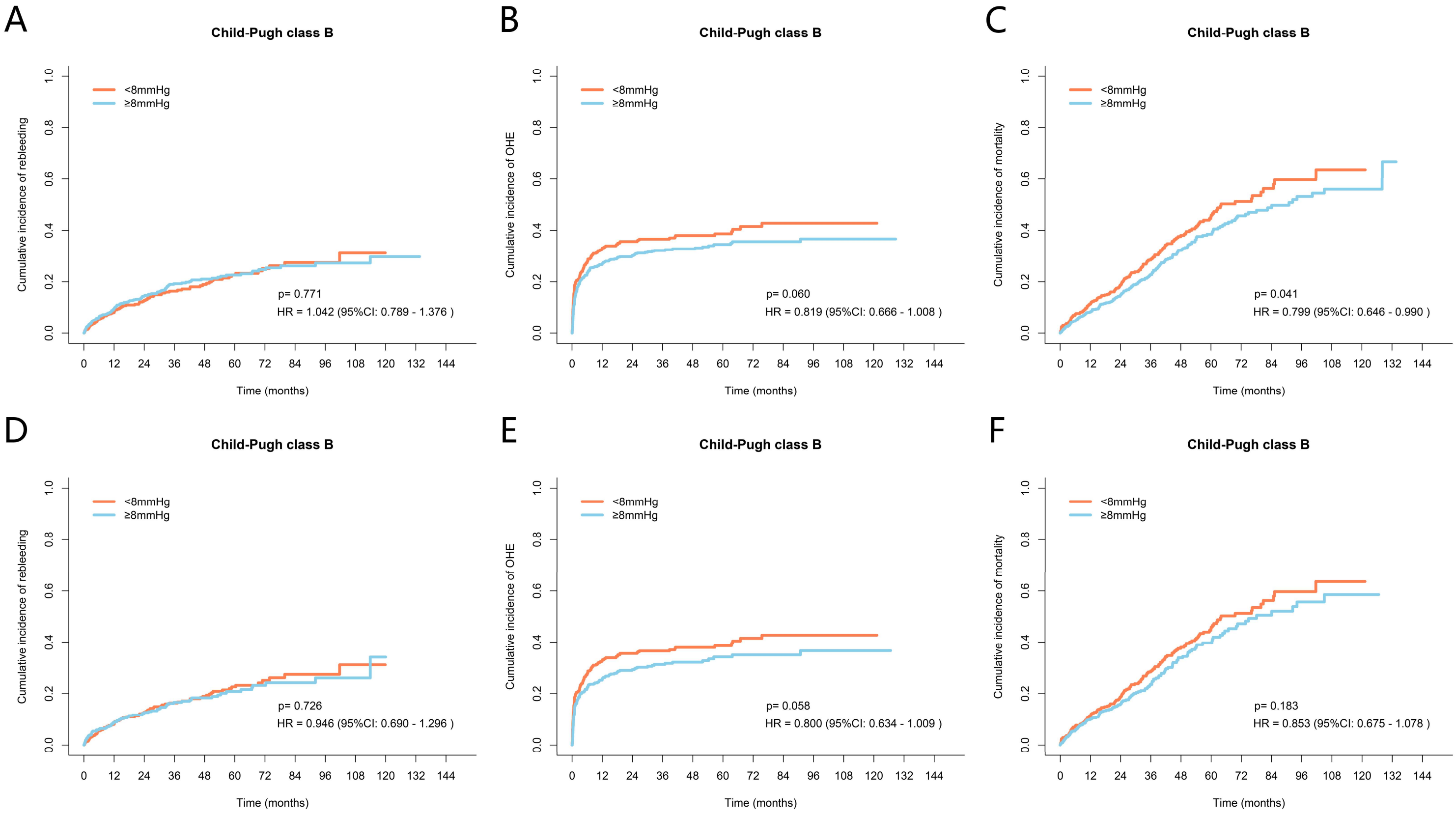

Supplementary Figure 23 Cumulative incidence of outcomes in Child-Pugh class B with 8mmHg post-TIPS PPG before and after PSM by competing risk analysis (Gray-Fine test).

(A) Cumulative incidence of rebleeding in Child-Pugh class B with 8mmHg threshold before PSM. (B) Cumulative incidence of OHE in Child-Pugh class B with 8mmHg threshold before PSM. (C) Cumulative incidence of mortality of Child-Pugh class B with 8mmHg threshold before PSM. (D) Cumulative incidence of rebleeding in Child-Pugh class B with 8mmHg threshold after PSM. (E) Cumulative incidence of OHE in Child-Pugh class B with 8mmHg threshold after PSM. (F) Cumulative incidence of mortality of Child-Pugh class B with 8mmHg threshold after PSM.

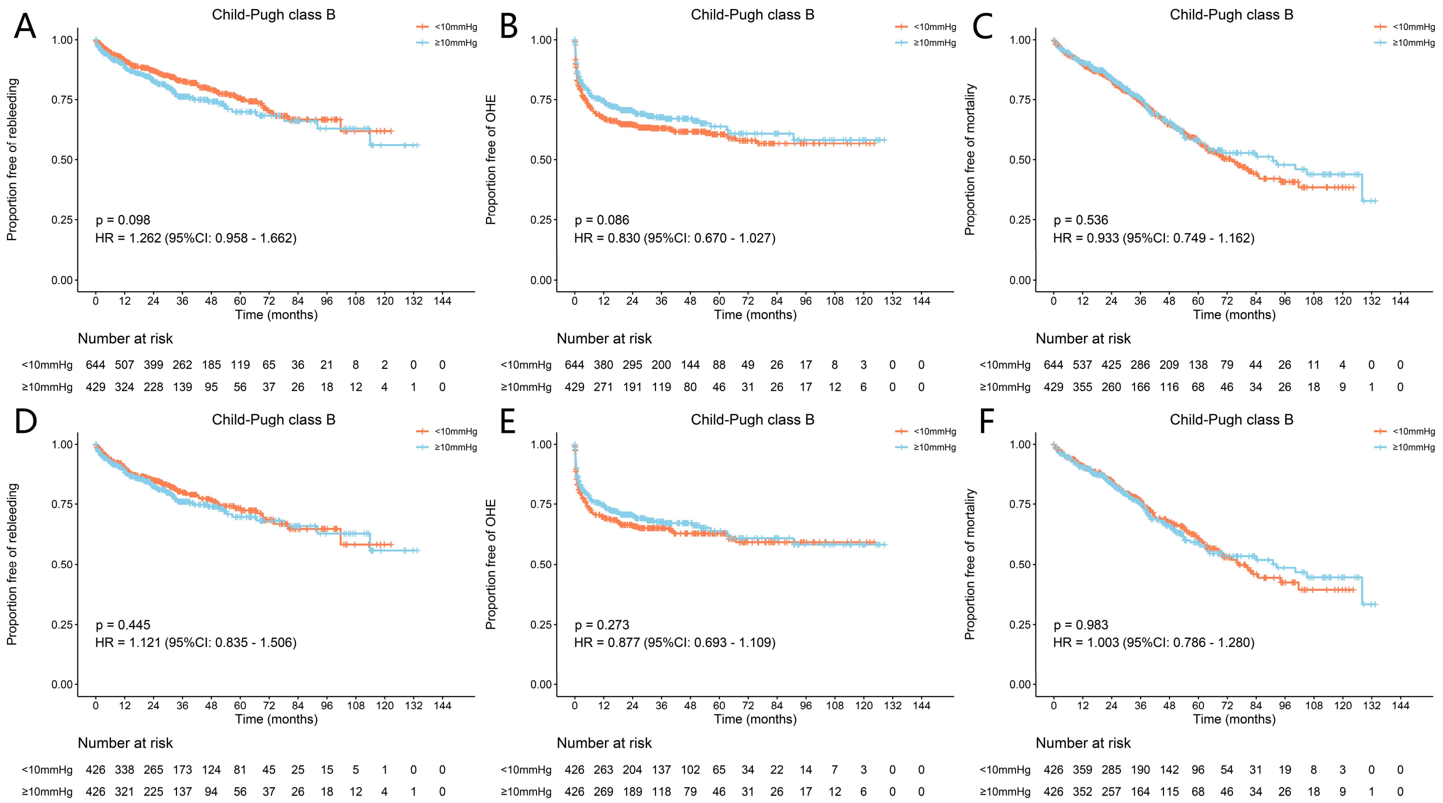

Supplementary Figure 24 Proportion free of outcomes in Child-Pugh class B with 10mmHg post-TIPS PPG before and after PSM.

(A) Proportion free of rebleeding in Child-Pugh class B with 10mmHg threshold before PSM. (B) Proportion free of OHE in Child-Pugh class B with 10mmHg threshold before PSM. (C) Proportion free of mortality of Child-Pugh class B with 10mmHg threshold before PSM. (D) Proportion free of rebleeding in Child-Pugh class B with 10mmHg threshold after PSM. (E) Proportion free of OHE in Child-Pugh class B with 10mmHg threshold after PSM. (F) Proportion free of mortality of Child-Pugh class B with 10mmHg threshold after PSM.

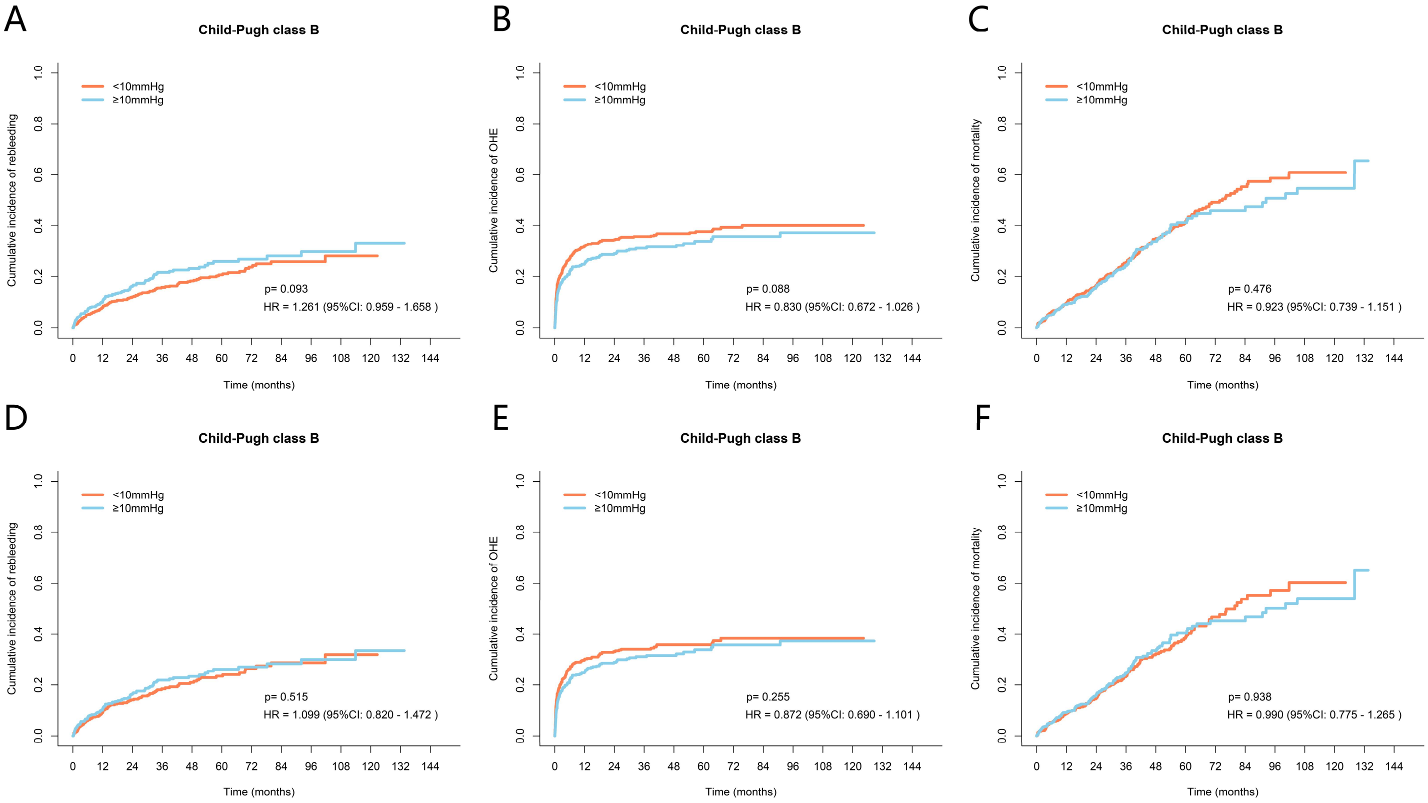

Supplementary Figure 25 Cumulative incidence of outcomes in Child-Pugh class B with 10mmHg post-TIPS PPG before and after PSM by competing risk analysis (Gray-Fine test).

(A) Cumulative incidence of rebleeding in Child-Pugh class B with 10mmHg threshold before PSM. (B) Cumulative incidence of OHE in Child-Pugh class B with 10mmHg threshold before PSM. (C) Cumulative incidence of mortality of Child-Pugh class B with 10mmHg threshold before PSM. (D) Cumulative incidence of rebleeding in Child-Pugh class B with 10mmHg threshold after PSM. (E) Cumulative incidence of OHE in Child-Pugh class B with 10mmHg threshold after PSM. (F) Cumulative incidence of mortality of Child-Pugh class B with 10mmHg threshold after PSM.

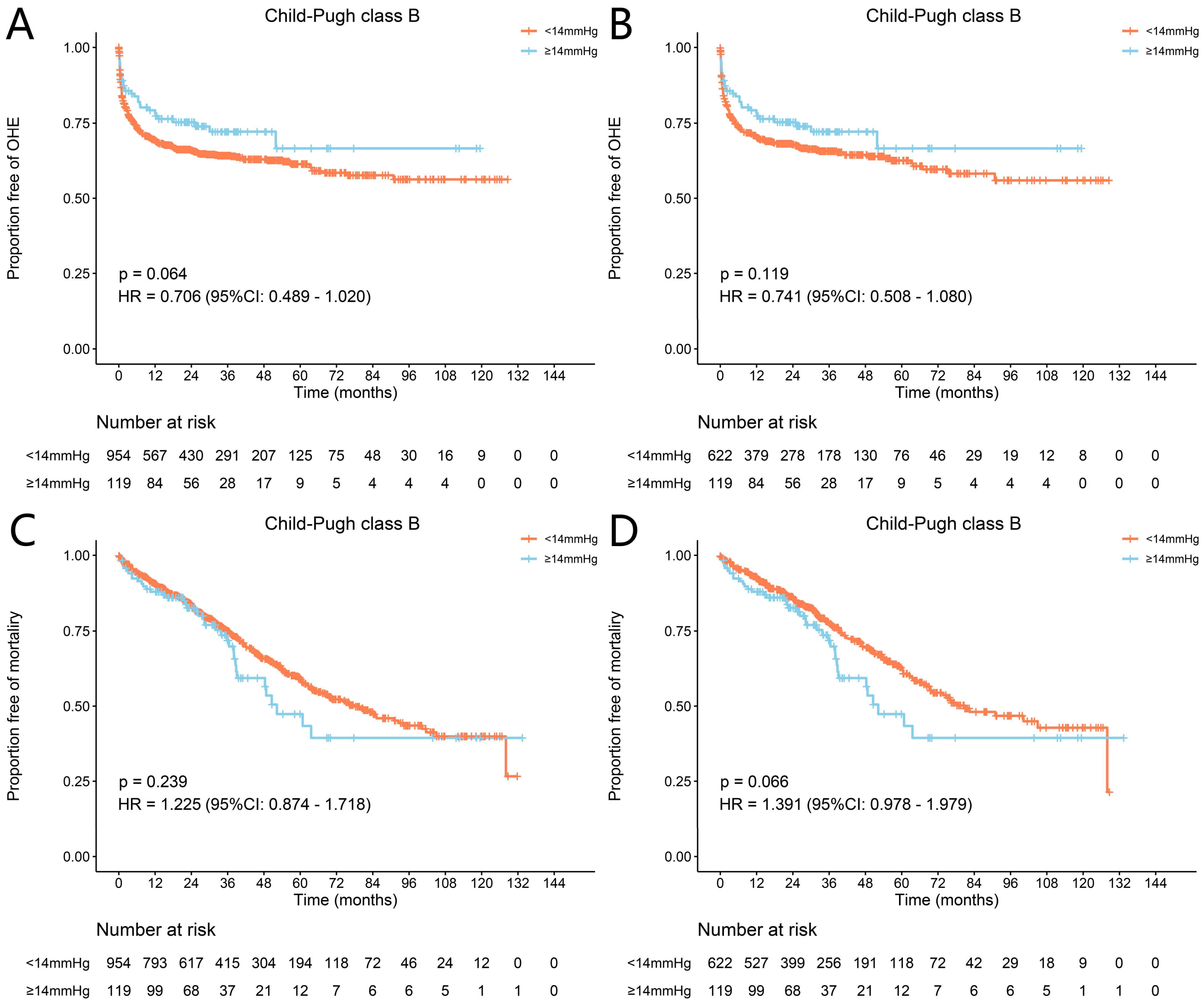

Supplementary Figure 26 Proportion free of OHE and mortality in Child-Pugh class B with 14mmHg post-TIPS PPG before and after PSM.

(A) Proportion free of OHE in Child-Pugh class B with 14mmHg threshold before PSM. (B) Proportion free of OHE in Child-Pugh class B with 14mmHg threshold after PSM. (C) Proportion free of mortality of Child-Pugh class B with 14mmHg threshold before PSM. (D) Proportion free of mortality of Child-Pugh class B with 14mmHg threshold after PSM.

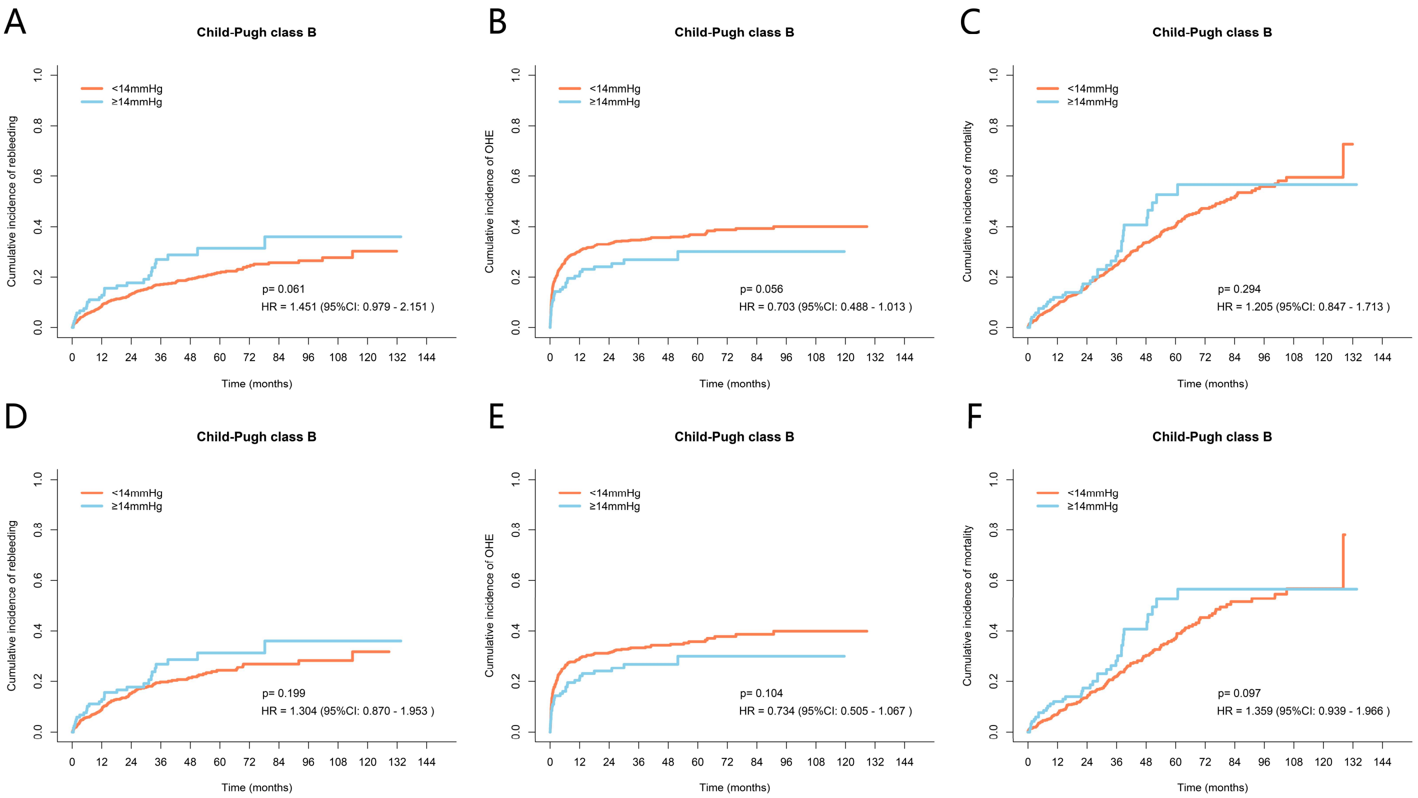

Supplementary Figure 27 Cumulative incidence of outcomes in Child-Pugh class B with 14mmHg post-TIPS PPG before and after PSM by competing risk analysis (Gray-Fine test).

(A) Cumulative incidence of rebleeding in Child-Pugh class B with 14mmHg threshold before PSM. (B) Cumulative incidence of OHE in Child-Pugh class B with 14mmHg threshold before PSM. (C) Cumulative incidence of mortality of Child-Pugh class B with 14mmHg threshold before PSM. (D) Cumulative incidence of rebleeding in Child-Pugh class B with 14mmHg threshold after PSM. (E) Cumulative incidence of OHE in Child-Pugh class B with 14mmHg threshold after PSM. (F) Cumulative incidence of mortality of Child-Pugh class B with 14mmHg threshold after PSM.

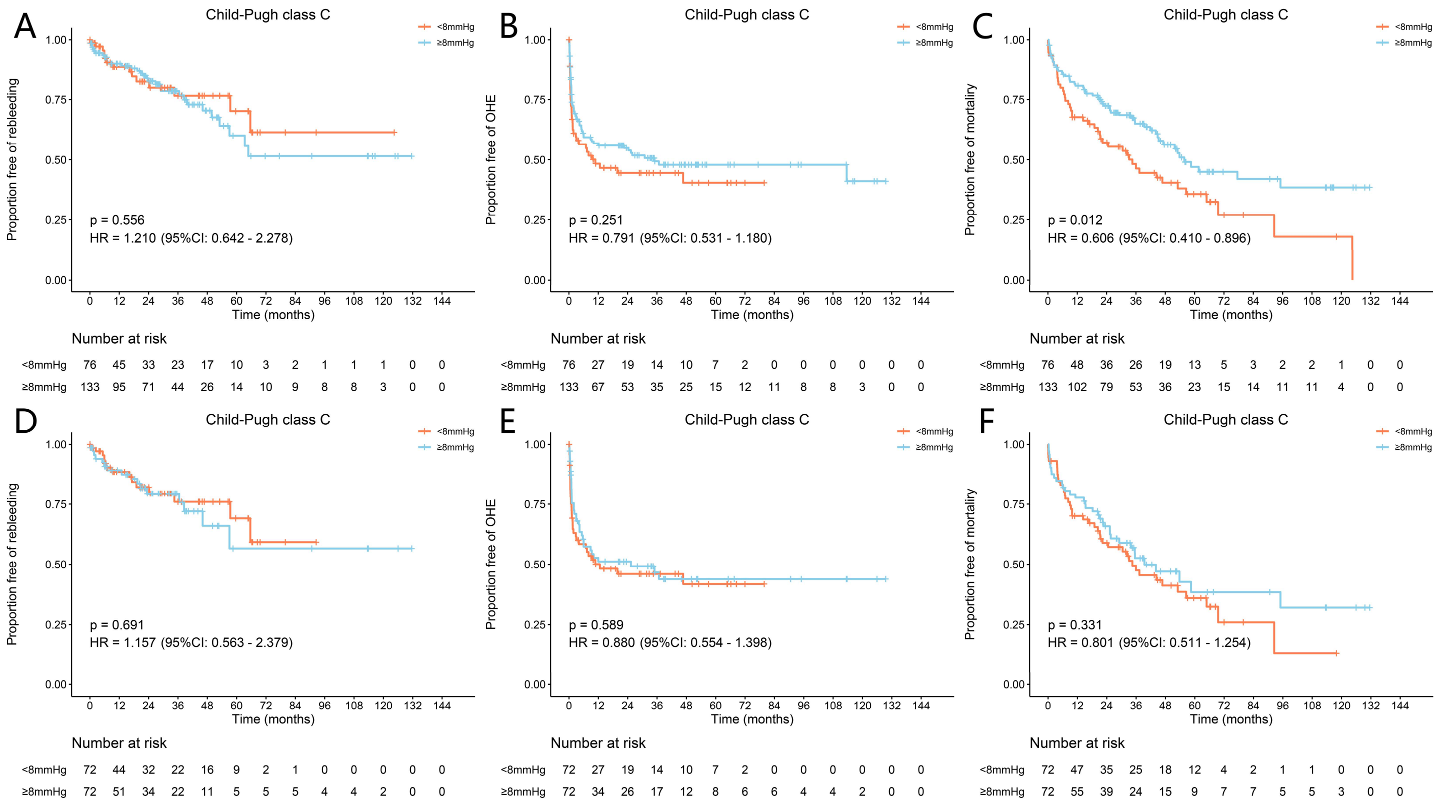

Supplementary Figure 28 Proportion free of outcomes in Child-Pugh class C with 8mmHg post-TIPS PPG before and after PSM.

(A) Proportion free of rebleeding in Child-Pugh class C with 8mmHg threshold before PSM. (B) Proportion free of OHE in Child-Pugh class C with 8mmHg threshold before PSM. (C) Proportion free of mortality of Child-Pugh class C with 8mmHg threshold before PSM. (D) Proportion free of rebleeding in Child-Pugh class C with 8mmHg threshold after PSM. (E) Proportion free of OHE in Child-Pugh class C with 8mmHg threshold after PSM. (F) Proportion free of mortality of Child-Pugh class C with 8mmHg threshold after PSM.

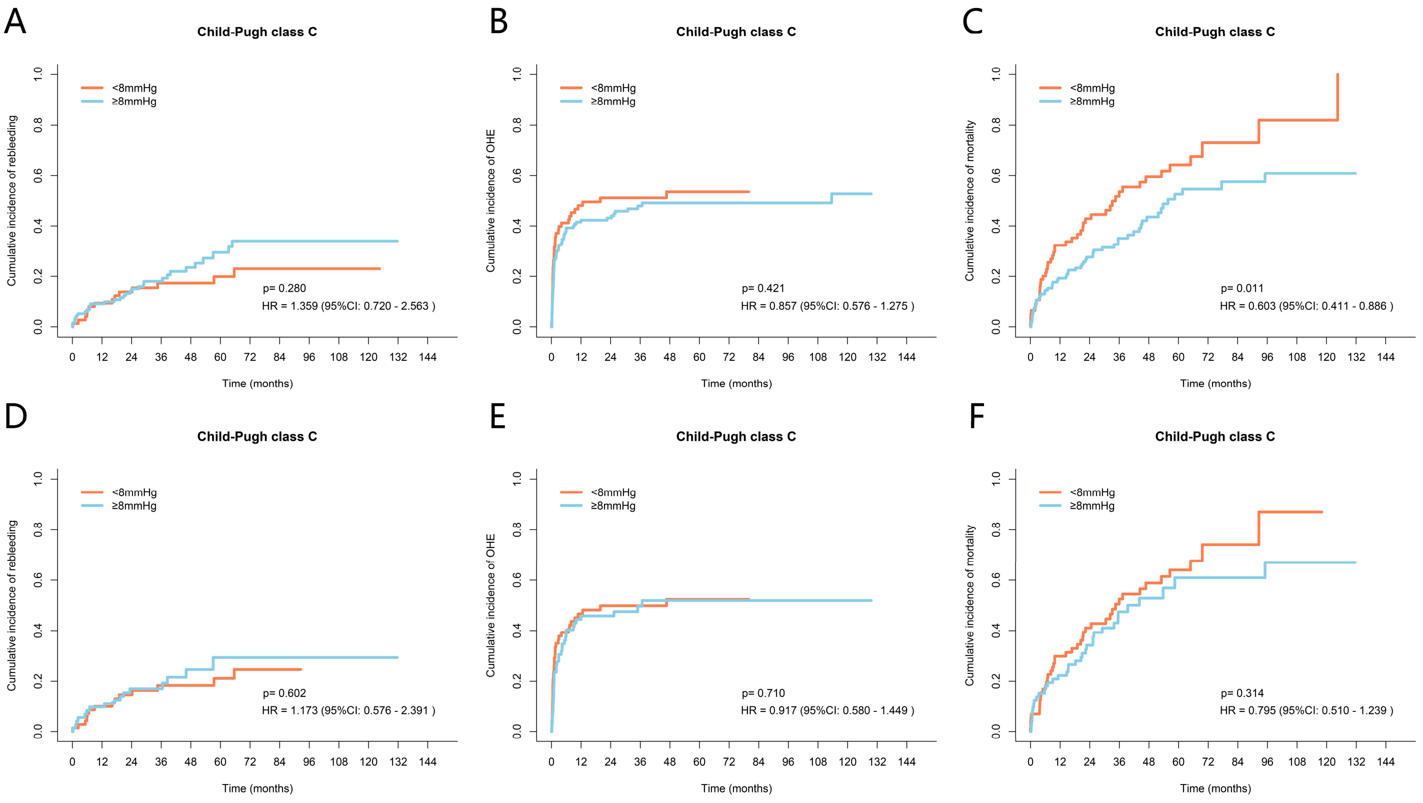

Supplementary Figure 29 Cumulative incidence of outcomes in Child-Pugh class C with 8mmHg post-TIPS PPG before and after PSM by competing risk analysis (Gray-Fine test).

(A) Cumulative incidence of rebleeding in Child-Pugh class C with 8mmHg threshold before PSM. (B) Cumulative incidence of OHE in Child-Pugh class C with 8mmHg threshold before PSM. (C) Cumulative incidence of mortality of Child-Pugh class C with 8mmHg threshold before PSM. (D) Cumulative incidence of rebleeding in Child-Pugh class C with 8mmHg threshold after PSM. (E) Cumulative incidence of OHE in Child-Pugh class C with 8mmHg threshold after PSM. (F) Cumulative incidence of mortality of Child-Pugh class C with 8mmHg threshold after PSM.

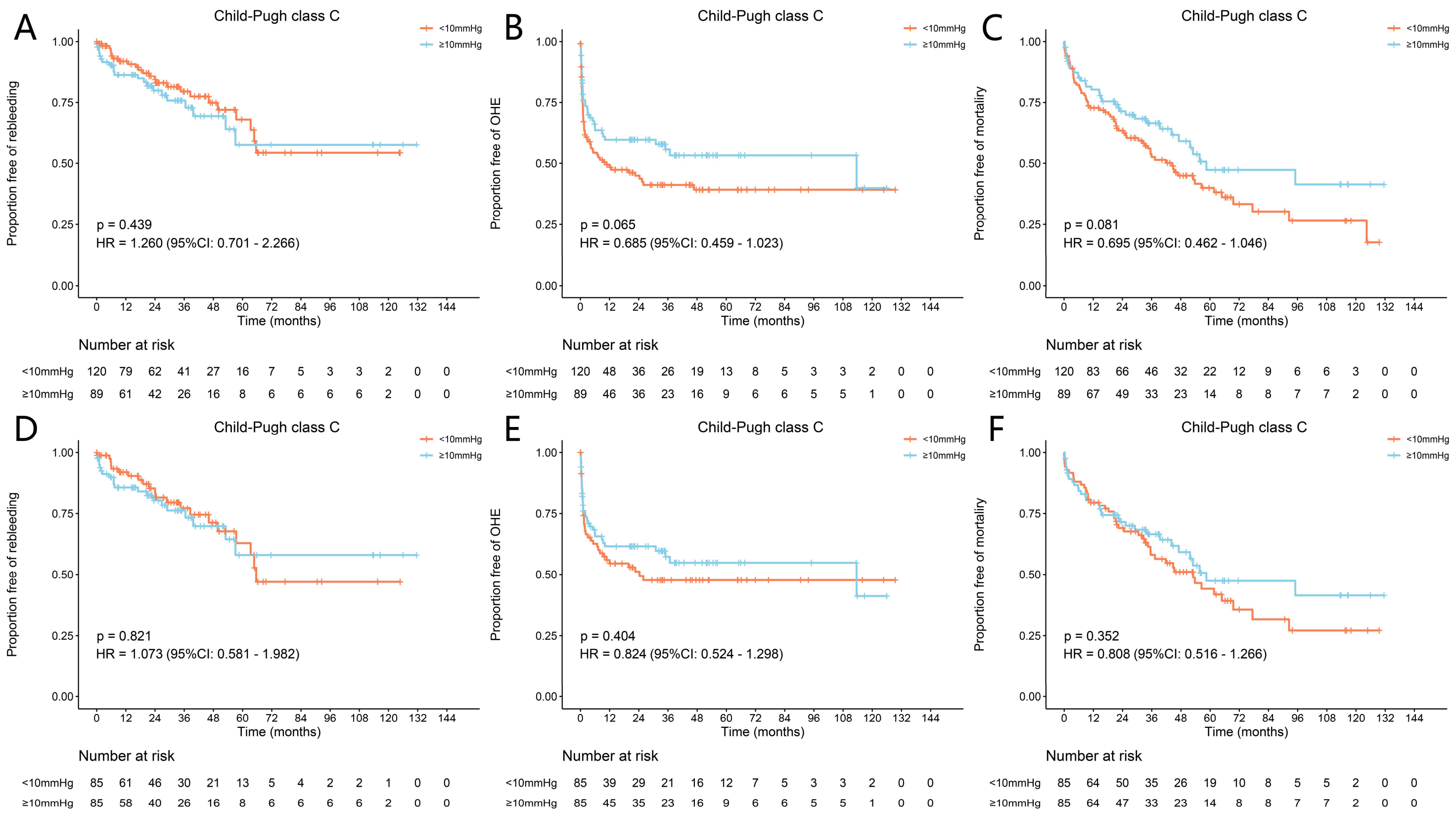

Supplementary Figure 30 Proportion free of outcomes in Child-Pugh class C with 10mmHg post-TIPS PPG before and after PSM.

(A) Proportion free of rebleeding in Child-Pugh class C with 10mmHg threshold before PSM. (B) Proportion free of OHE in Child-Pugh class C with 10mmHg threshold before PSM. (C) Proportion free of mortality of Child-Pugh class C with 10mmHg threshold before PSM. (D) Proportion free of rebleeding in Child-Pugh class C with 10mmHg threshold after PSM. (E) Proportion free of OHE in Child-Pugh class C with 10mmHg threshold after PSM. (F) Proportion free of mortality of Child-Pugh class C with 10mmHg threshold after PSM.

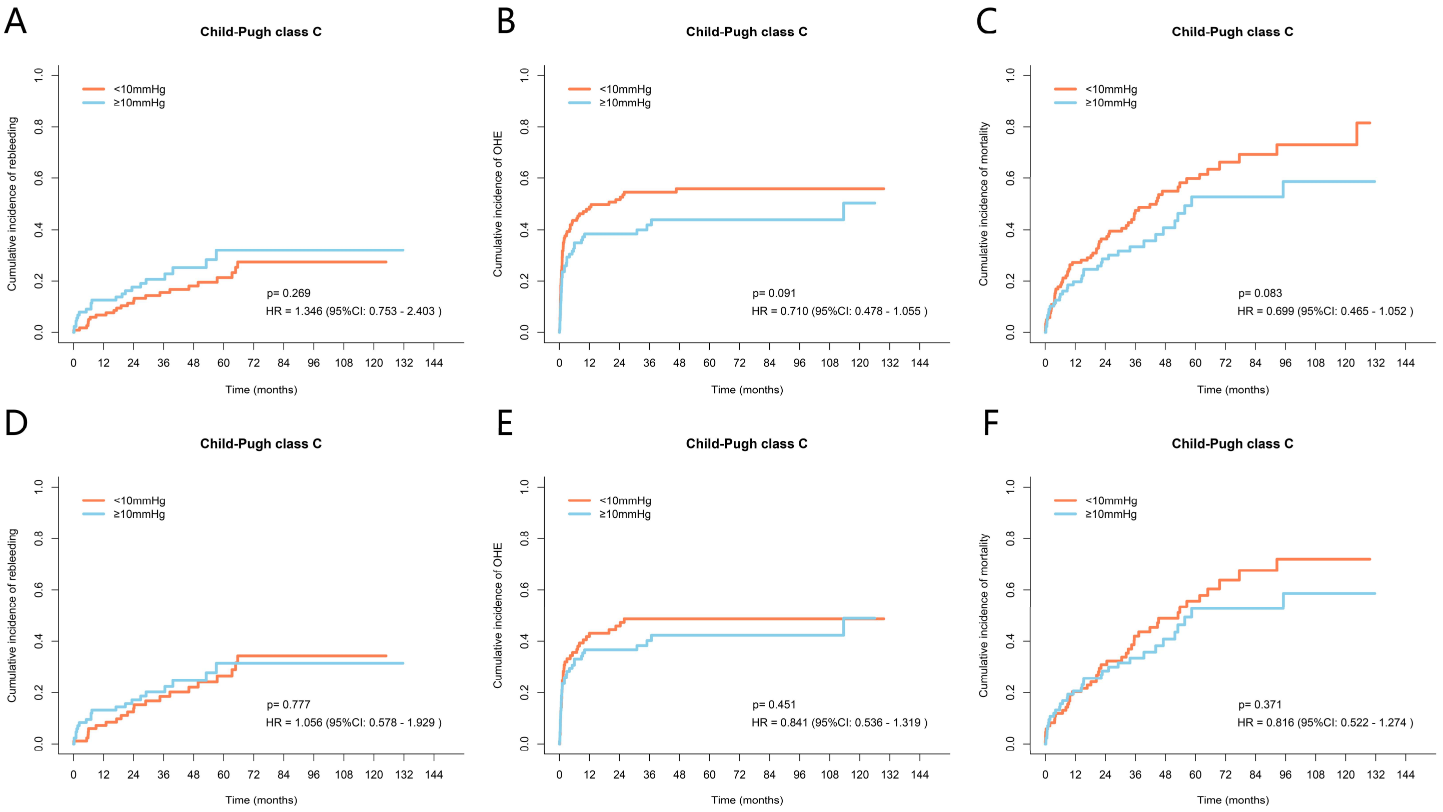

Supplementary Figure 31 Cumulative incidence of outcomes in Child-Pugh class C with 10mmHg post-TIPS PPG before and after PSM by competing risk analysis (Gray-Fine test).

(A) Cumulative incidence of rebleeding in Child-Pugh class C with 10mmHg threshold before PSM. (B) Cumulative incidence of OHE in Child-Pugh class C with 10mmHg threshold before PSM. (C) Cumulative incidence of mortality of Child-Pugh class C with 10mmHg threshold before PSM. (D) Cumulative incidence of rebleeding in Child-Pugh class C with 10mmHg threshold after PSM. (E) Cumulative incidence of OHE in Child-Pugh class C with 10mmHg threshold after PSM. (F) Cumulative incidence of mortality of Child-Pugh class C with 10mmHg threshold after PSM.

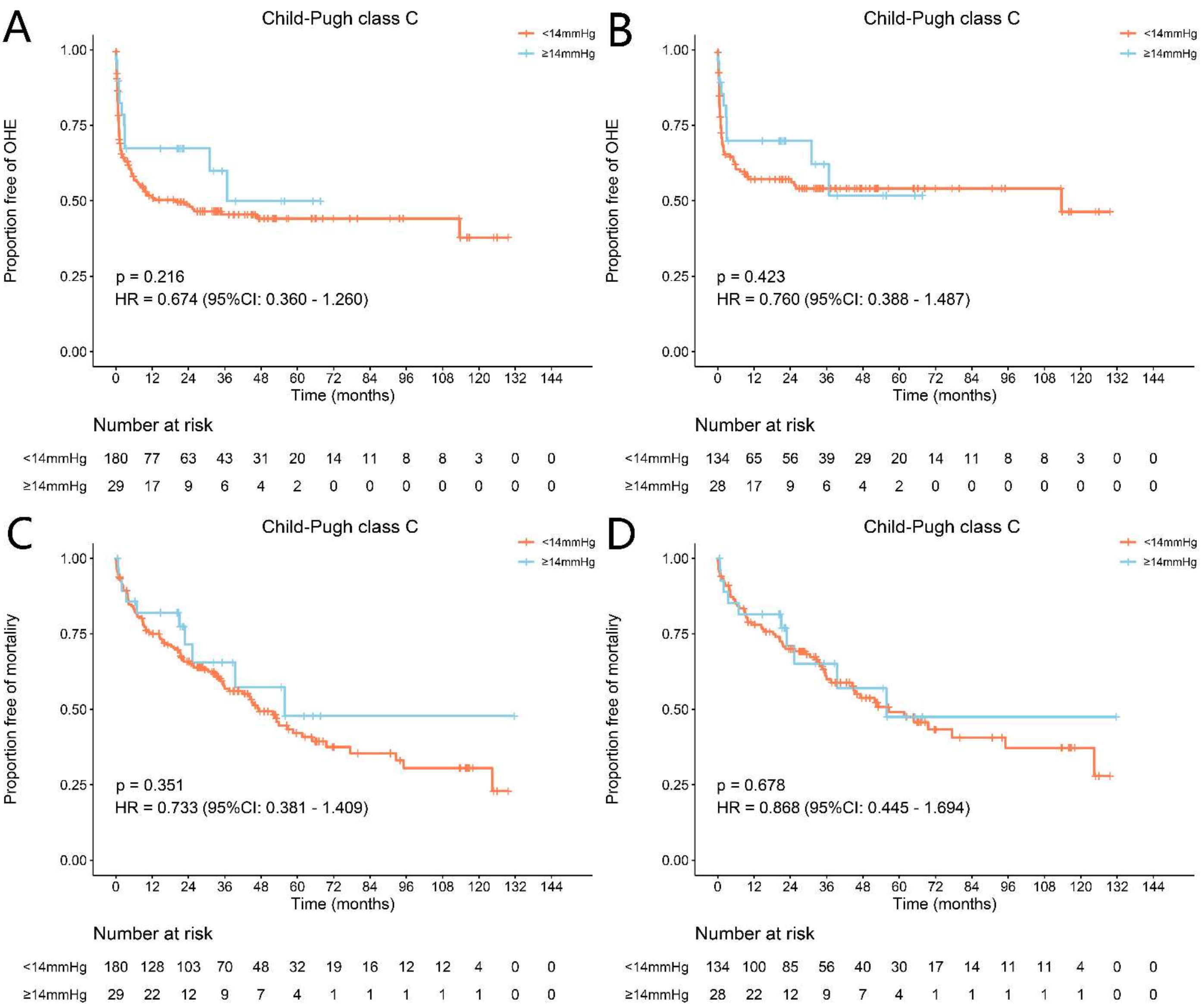

Supplementary Figure 32 Proportion free of OHE and mortality in Child-Pugh class C with 14mmHg post-TIPS PPG before and after PSM.

(A) Proportion free of OHE in Child-Pugh class C with 14mmHg threshold before PSM. (B) Proportion free of OHE in Child-Pugh class C with 14mmHg threshold after PSM. (C) Proportion free of mortality of Child-Pugh class C with 14mmHg threshold before PSM. (D) Proportion free of mortality of Child-Pugh class C with 14mmHg threshold after PSM.

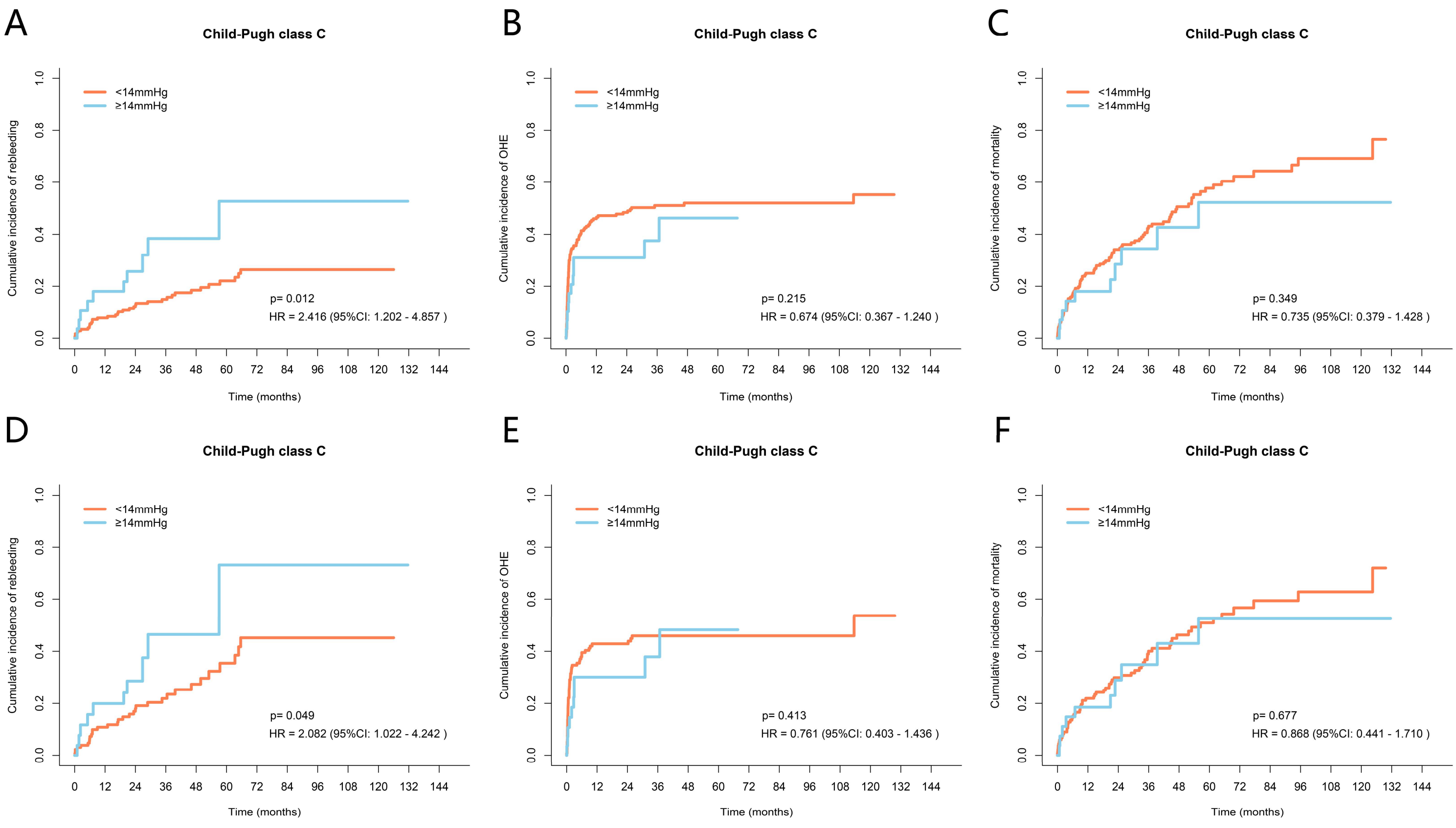

Supplementary Figure 33 Cumulative incidence of outcomes in Child-Pugh class C with 14mmHg post-TIPS PPG before and after PSM by competing risk analysis (Gray-Fine test).

(A) Cumulative incidence of rebleeding in Child-Pugh class C with 14mmHg threshold before PSM. (B) Cumulative incidence of OHE in Child-Pugh class C with 14mmHg threshold before PSM. (C) Cumulative incidence of mortality of Child-Pugh class C with 14mmHg threshold before PSM. (D) Cumulative incidence of rebleeding in Child-Pugh class C with 14mmHg threshold after PSM. (E) Cumulative incidence of OHE in Child-Pugh class C with 14mmHg threshold after PSM. (F) Cumulative incidence of mortality of Child-Pugh class C with 14mmHg threshold after PSM.

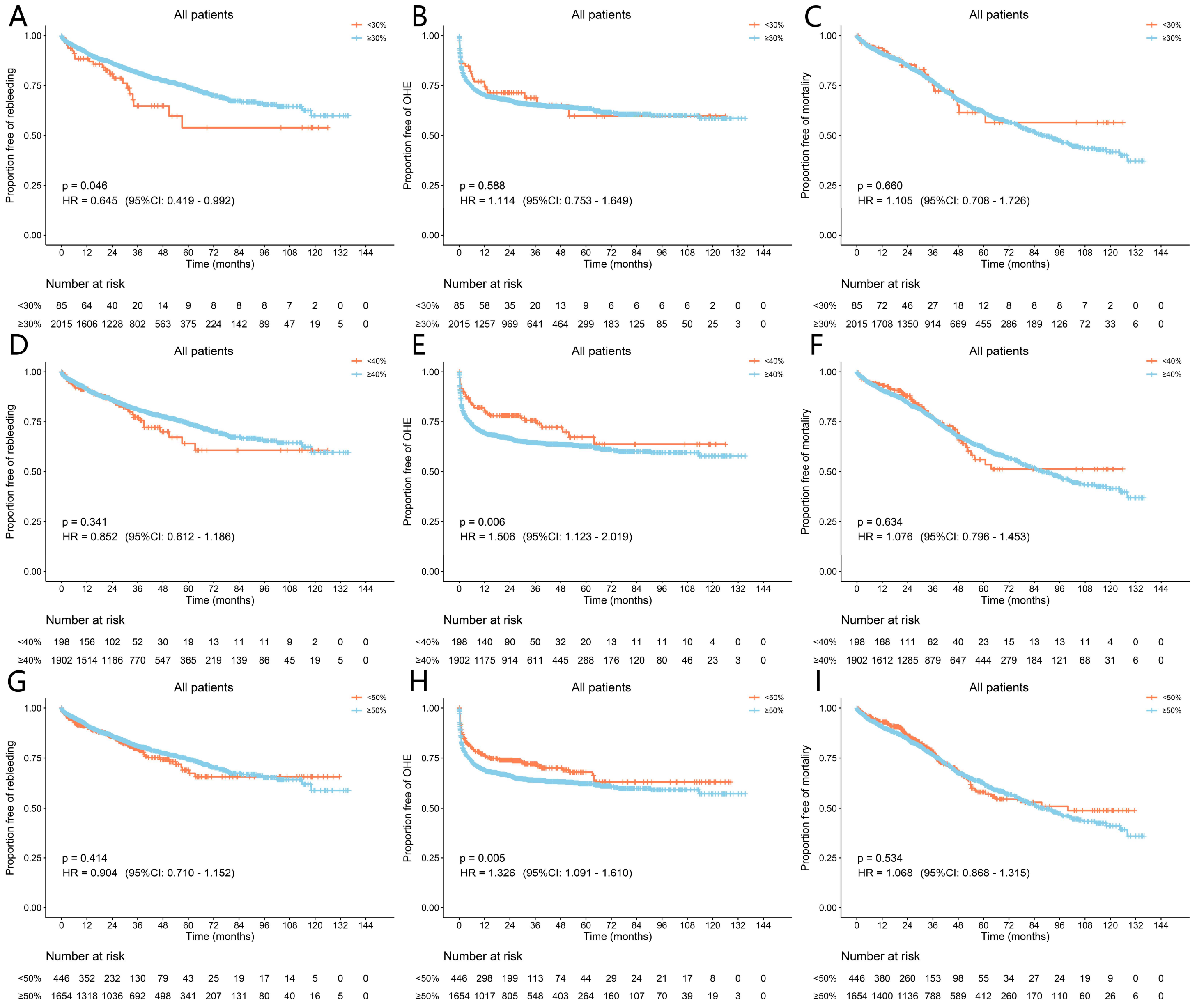

Supplementary Figure 34 Proportion free of outcomes in all patients with 30%, 40% and 50% decrease of PPG.

(A) Proportion free of rebleeding in all patients with 30% decrease of PPG. (B) Proportion free of OHE in all patients with 30% decrease of PPG. (C) Proportion free of death in all patients with 30% decrease of PPG. (D) Proportion free of rebleeding in all patients with 40% decrease of PPG. (E) Proportion free of OHE in all patients with 40% decrease of PPG. (F) Proportion free of death in all patients with 40% decrease of PPG. (G) Proportion free of rebleeding in all patients with 50% decrease of PPG. (H) Proportion free of OHE in all patients with 50% decrease of PPG. (I) Proportion free of

death in all patients with 50% decrease of PPG.
